# A Study of Intramolecular Hydrogen Bonding in Levoglucosan Derivatives

**DOI:** 10.3390/molecules22040518

**Published:** 2017-03-24

**Authors:** Lucas Quiquempoix, Elena Bogdan, Neil J. Wells, Jean-Yves Le Questel, Jérôme Graton, Bruno Linclau

**Affiliations:** 1 Chemistry, University of Southampton, Highfield, Southampton SO17 1BJ, UK, lq1d13@soton.ac.uk (L.Q.); n.j.wells@soton.ac.uk (N.J.W.); 2 CEISAM UMR CNRS 6230, Faculté des Sciences et des Techniques, Université de Nantes, 2 rue de la Houssinière—BP 92208, 44322 Nantes CEDEX 3, France; elena_bogdan@hotmail.fr

**Keywords:** hydrogen bond, intramolecular interaction, NMR coupling constants, quantum calculations, fluorination, levoglucosan

## Abstract

Organofluorine is a weak hydrogen-bond (HB) acceptor. Bernet et al. have demonstrated its capability to perturb OH···O intramolecular hydrogen bonds (IMHBs), using conformationally rigid carbohydrate scaffolds including levoglucosan derivatives. These investigations are supplemented here by experimental and theoretical studies involving six new levoglucosan derivatives, and complement the findings of Bernet et al. However, it is shown that conformational analysis is instrumental in interpreting the experimental data, due to the occurrence of non-intramolecular hydrogen-bonded populations which, although minor, cannot be neglected and appears surprisingly significant. The DFT conformational analysis, together with the computation of NMR parameters (coupling constants and chemical shifts) and wavefunction analyses (AIM, NBO), provides a full picture. Thus, for all compounds, the most stabilized structures show the OH groups in a conformation allowing IMHB with O5 and O6, when possible. Furthermore, the combined approach points out the occurrence of various IMHBs and the effect of the chemical modulations on their features. Thus, two-center or three-center IMHB interactions are observed in these compounds, depending on the presence or absence of additional HB acceptors, such as methoxy or fluorine.

## 1. Introduction

Hydrogen bonding (H-bonding) to organic fluorine (C-F) represents a very weak interaction [1,2]. Given bioactive compounds typically contain stronger oxygen and/or nitrogen (O/N) based hydrogen-bond (HB) acceptor atoms, intra- or intermolecular HB formation, in solution or in the solid phase, will preferentially involve these groups over a C-F group. The same applies for polar protic solvents (including water). This has been amply demonstrated by crystal structure analysis of fluorinated compounds, where O/N-based HB donors typically form intramolecular (IM) hydrogen bonds with O/N based HB acceptors instead of with fluorine, even when the molecule in question contains many fluorine atoms [3,4]. This effect can lead to interesting crystal packing structures, featuring different molecular conformations in the unit cell in order to maximize, say, OH···O, at the expense of OH···F interactions [5].

However, in the absence of competing HB acceptors, alcohol groups have shown to form intramolecular HBs with fluorine groups. First described by Biamonte and Vasella [6], many groups have now reported such OH···F IMHBs through the observation of an OH-F coupling constant (^1^H-NMR) [1,7]. The vast majority of these cases involve conformationally constrained systems, though the occurrence of OH···F IMHB have recently been demonstrated in acyclic fluorohydrins [8].

**Figure 1:**
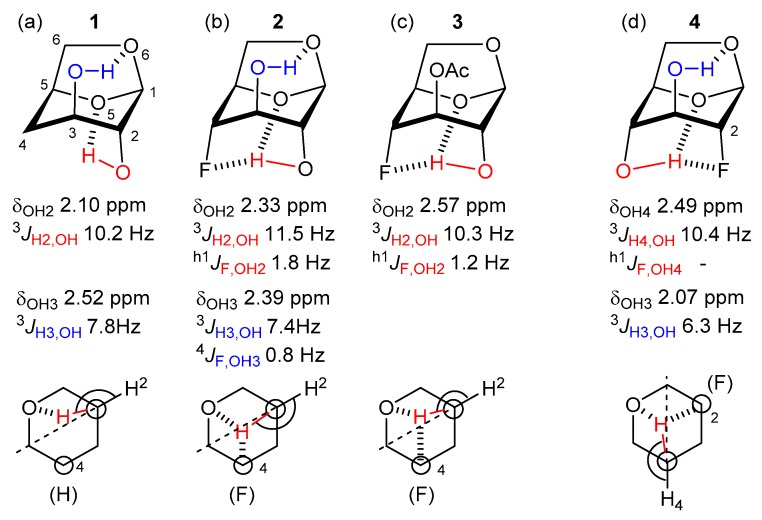
Selected precedence involving intramolecular OH···F hydrogen bonding.

In this framework, an interesting and relevant challenge is to determine whether existing hydrogen bonds present in a molecule can be influenced by the HB accepting properties of fluorine. This has been elegantly investigated by Bernet and Vasella through NMR-analysis of a set of rigid 4-fluorinated levoglucosan derivatives, by using the ^3^*J*_H-OH_ value to deduce the dihedral angle revealing positional information of the alcohol hydrogen (NMR spectra taken in CDCl_3_) [9]. For example (Figure 1a–c), in the 4-deoxy levoglucosan **1**, the ^3^*J*_H2-OH_ value of 10.2 Hz indicates that the OH is directed towards the O5 acetal oxygen because of IM H-bonding. When a fluorine atom is positioned at C4, as in **2**, this value increases to 11.5 Hz, indicating a larger H2-OH dihedral angle. This is explained by the axial fluorine group competing with O5 for hydrogen bonding to the OH, causing a shift in the OH position. The observed ^h1^*J*_F-OH_ value corroborates this explanation. Interestingly, in **3**, the OH···F distance is increased compared to **2**, as evidenced by smaller ^h1^*J*_F-OH_ value, which had been explained by the increased electron withdrawing effect of the acetate group (compared to an OH-group that acts as HB donor), resulting in the fluorine becoming a poorer HB acceptor. By using this methodology on 4-fluorinated and 4,4-difluorinated 4-deoxymannose derivatives, Bernet and Gouverneur could nicely demonstrate that fluorine as part of a CF_2_ group is a worse HB acceptor than when part of a CHF moiety (not shown) [10], in agreement with measurements of intermolecular OH···F interactions [11]. Interestingly, Widmalm et al. reported a study of 2-deoxy-2-fluorolevoglucosan **4** (Figure 1d), which has a similar IMHB arrangement as **2**/**3**, but for which no ^h1^*J*_F-OH_ coupling could be observed [12]. This was also explained by the decreased HB accepting capacity of the fluorine group due to the antiperiplanar C-O6 group. This is despite the ^3^*J*_H4,OH_ value in **4** is identical to the ^3^*J*_H2,OH_ value in **3**.

It has been shown that these weak interactions can affect properties such as intermolecular hydrogen bond donating capacity [13] and reactivity [6] of alcohols, and conformational properties of acyclic alcohols [8]. Given the importance of inter- and intramolecular hydrogen bonding and conformation in many areas [14,15], the study of these weak interactions, and the factors that influence them, is of interest.

**Figure 2:**
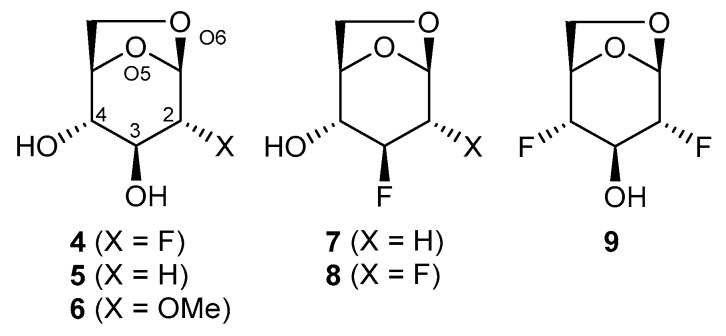
Structures of levoglucosan derivatives used in this study.

In the current work, we have selected levoglucosan derivatives **4**–**9** (Figure 2) to expand the study of intramolecular hydrogen bonding properties. The substrate pairs **4**/**5** and **7**/**8** allow studying the perturbation of the OH4···O5 IMHB by F, while **6** allows to compare the effect of F with OMe. For **4**–**6** we also noticed interesting substituent effects affecting the OH3···O6 IMHB, and substrate **9** was also included in this context. The computational calculations usefully complemented the experimental results by showing that structural analyses based on ^h1^*J*_OH···F_ and ^3^*J*_H,OH_ coupling constants have to be carefully interpreted in combination with theoretical studies, given the population of the IM H-bonded conformers is in some cases far from 100%. In addition, estimated IMHB distances and strengths were available through computational calculations.

## 2. Results

### 2.1. Synthesis of the Levoglucosan Derivatives

**Scheme 1:**
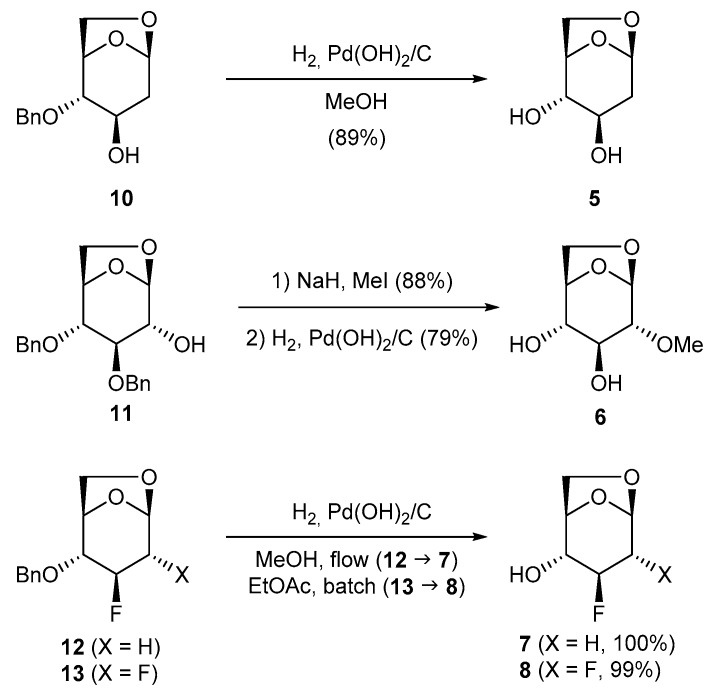
Synthesis of the novel levoglucosan derivatives.

Levoglucosan derivatives **5** [16], **7**, and **8** [17] were obtained by hydrogenolysis using Pearlman’s catalyst of the known **10** [18], **12** [18], and **13** [17,18] in excellent yields while **6** [19] was obtained in a two steps sequence methylation-hydrogenolysis of the known **11** [20] (Scheme 1). The known compounds **4** [12,21] and **9** [21,22] were obtained as described.

### 2.2. Experimental and Theoretical NMR Features

The measurement of the NMR spectra was carried out under carefully controlled anhydrous conditions in dilute chloroform solution (solute concentration between 7–19 mM) at 25 °C. The summary of the chemical shift and coupling constant values is given in Table 1. The data were collected such that the digital resolution was 0.05 Hz/point. Interestingly, a control experiment in which a 7.5 mM solution of **4** was spiked with 1 equiv of H_2_O showed no change in the ^3^*J*_OH-H_ and ^h1^*J*_OH-F_ values (see supporting information).

The theoretical coupling constant and chemical shift values involving the levoglucosan’s OH3 and OH4 hydroxyl groups have been computed at the B97-2/pcJ-2 level, on geometries optimized at the B97-D3^BJ^/6-311++G(2d,p) level in chloroform at 25 °C (discussed below). These computed parameters correspond to the weighting of the individual values calculated for each conformer by their respective relative populations according to Equation (1) in the Materials and Methods section, and are also shown in Table 1. It is important to note that the calculated ^3^*J* coupling constants are systematically overestimated compared to the experimental data. Nevertheless, the relative experimental and theoretical trends are on the whole consistent. Thus, regarding the chemical shift values order, a perfect agreement with the observed δ_OH4_ is obtained, with the ranking **5** < **7** < **4** < **8** < **6**. For the δ_OH3_ values, the theoretical results confirm the behavior **5** > **6** > **4**, but not for **9**, where a weaker theoretical value is computed.

### 2.3. Conformational Analysis of the Levoglucosans

**Figure 3:**
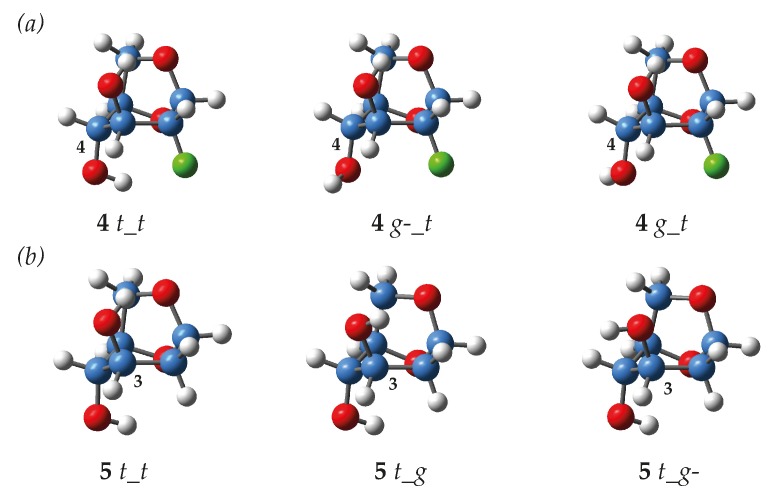
Examples of optimized structures of (**a**) levoglucosan **4**, with the OH4 group in *t*, *g-*, and *g* orientation; (**b**) levoglucosan **5**, with the OH3 group in *t*, *g* and *g-* orientation.

Observed *J* values are averaged over the populations of the possible conformers. For the compounds under study, the identified degrees of freedom are the rotation around the C-O bonds of the hydroxyl groups, and of the methoxy group in **6**. Hence, when comparing coupling constants between molecules, the relative population of the relevant conformers needs to be considered. The conformers are defined by their values of the ϕ_HCOH_ dihedral angles: *trans* (*t*) if ϕ_HCOH_ ~180°, *gauche* (*g*) when ϕ_HCOH_ ~60°, and *gauche(-)* (*g-*) when ϕ_HCOH_ ~−60°, and similarly for the ϕ_HCOC_ dihedral angle for the methoxy group. For example, the notation *t_g-_g* depicts a *trans* orientation around the C-4 bond, a *gauche(-)* conformation around the C-3 bond and finally a *gauche* orientation around the C-2 bond. The whole dataset is reported in the Supplementary Materials, and a summary of the most important conformers involving OH4 is given in Table 2, and of OH3 in Table 3. Figure 3 illustrates typical geometries encountered with the various conformations around the OH4 and OH3 hydroxyl groups. For all structures, the *t_t* forms are systematically the most stabilized conformers meaning that both hydroxyl moieties are involved in OH···O intramolecular interactions.

### 2.4. Levoglucosans Wavefunction Analyses

AIM wavefunction analyses in the surroundings of the hydroxyl moieties have been performed to compare the different interactions operating in the various levoglucosans and to provide a better understanding of their respective structural features, as illustrated in Table 4. Bond critical points (BCPs) related to six-membered OH···O IMHB interactions were systematically detected, whereas we were not able to find any BCP along the OH4···F and the OH4···O5 interactions. This latter defines a five-membered IMHB motifs and the AIM methodology is known to fail detecting any BCP in these conditions [23,24,25]. The interaction energies *E*^(2)^_n→σ*_ computed from the NBO analyses pointed out charge transfer from the oxygen or the fluorine lone pairs to the hydroxyl antibonding orbital whatever the IMHB studied, but they clearly show that these interactions are very weak for the five-membered IMHBs, coherent with the AIM trends.

## 3. Discussion

### 3.1. Description of the Intramolecular OH4 Interactions

#### 3.1.1. IMHB Involving OH4

**Figure 4:**
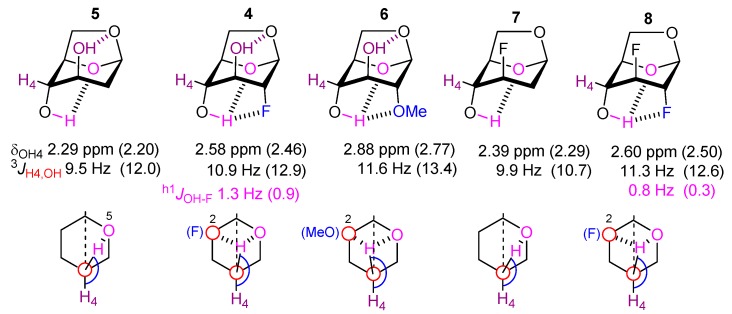
NMR features of the OH4 surroundings in compounds **4**–**8**. The theoretical data are given in parentheses.

The IMHB features of the OH4 groups of **4**–**6** are discussed first. As shown in Table 1 and in Figure 4, the ^3^*J*_OH4-H_ value for **5**, with no C2 substituent, gradually increases with the incorporation of a fluorine (**4**) and methoxy group (**6**) at C2. This can be explained following the arguments used by Bernet et al. [9]: while in **5** there is only IMHB with O5, in **4** and **6** there is a competition between the O5 atom and the introduced F or OMe substituents as HB acceptors for interaction with OH4. The OH4 hydrogen atom is therefore positioned further away from O5, leading to a steady increase in H4-OH4 dihedral angle from **5** < **4** < **6**, as shown in Figure 4. This is consistent with the increasing experimental ^3^*J*_H4-OH_ values, which almost reach the maximum coupling constant of 12.5 Hz expected for an averaged dihedral angle of 180°. It is noted that for **6**, OH4 is computed to become closer to the OMe group than to O5 (change in sign of dihedral angle) in the most stable conformers.

These findings are corroborated by the calculated intramolecular distances involving OH4, and by the NBO interaction energies. The shift in IMHB from O5 to F2 and OMe2 is reflected in an increase in d_OH4···O5_ distances (see Table 2) from **5** < **4** < **6**. The NBO interaction energies *E*^(2)^_n→σ*_ which correspond to the charge transfer from the O5 lone pair to the OH4 antibonding orbital (n_O5_ → σ*_OH4_), concomitantly decrease from **5** > **4** > **6** (Table 4), demonstrating the decrease of the efficiency of this charge transfer when a competing acceptor is present at C2. Correspondingly, the NBO interaction energies involving OH4 and the substituent at C2 increase going from fluorine to methoxy. This is consistent with the worse HB acceptor ability of fluorine compared to oxygen. Interestingly, the NBO interaction energies involving the OH4 group in **4** also show that the interaction with O5 is still stronger than the interaction with F2. This is supported by the AIM analyses, for which we only found BCPs between the OH4 and methoxy substituents in **6**, with weak electron densities (from 0.007 to 0.012 e∙bohr^−3^) typical of a HB interaction. The *E*_HB_ parameter, calculated from the potential energy density *V*_bcp_ and ranging from 7 to 11 kJ∙mol^−1^ for this compound, also indicates a weak IMHB interaction. Conversely, we never observed any BCP between the hydroxyl group and the fluorine atom for the relevant conformers of **4** (and **8**). In fact, one can notice that the interaction with the fluorine atom in the *t_t*, *t_g*, and *t_g-* conformations of **4** is longer than the sum of the fluorine and hydrogen van der Waals radii (2.57 Å) [26]. This intramolecular interaction would, hence, be too long to be considered as an IMHB, but it is clearly an attractive interaction significant enough to slightly weaken the OH4···O5 IMHB, and to result in a ^h1^*J*_OH-F_ coupling.

The chemical shift value of OH groups is also an indicator of the extent of intramolecular hydrogen bonding. However, the situation described here is a special case as a shift in IMHB from one atom (O5) to other substituents is occurring. Nevertheless, the data show that H4 becomes progressively more deshielded when it is involved in three-centered IMHB (**5** < **4** < **6**), with δ_OH4_ being correlated with the ^3^*J*_OH4-H_ value.

Finally, the computed features for **7** and **8** show that the C3 fluorine atom has an equivalent effect on the OH4···O5 IMHB than an OH3 hydroxyl group, but only when OH3 is not H-bonded to O6 (Table 2). Hence, for the *t_g* forms of **5** and **4**, the OH4···O5 distances, the dihedral angles and the corresponding OH4 chemical shifts are all similar. Since the *t_t* conformers exhibit lower δ_OH4_ values, the higher experimental (averaged) δ_OH4_ values of **7** and **8** with respect to **5** and **4**, are explained by the higher population of *t_t* conformers of the latter.

Small through space ^h1^*J*_OH4···F_ couplings were observed in both 2-fluorinated compounds **4** and **8** (1.4 and 0.8 Hz, respectively), not detected by Widmalm et al [12] in the NMR spectra of **4**. The smaller ^h1^*J*_OH4···F_ for **8** indicates a longer averaged OH4···F2 distance. Indeed, if they show similar amounts of IMHB conformers (Table 2), **8** is calculated to exhibit a OH4···F2 distance (2.840 Å) larger than **4** (2.688 Å, weighted value).

Hence, the observation from Bernet that vicinal dihedral angles are a good indicator to reveal IMHB is confirmed here. However, for compound **5**, the OH4 *trans* conformers are only found to represent 87% of the whole population (see Table 2) and, 13% of the OH4 conformers involve a *gauche* OH4 dihedral angle for which the ^3^*J*_OH4-H_ values are much lower. This is obviously reflected in the experimental (averaged) ^3^*J*_OH4-H_ value, and also in the δ_OH4_ value (2.29/2.20 ppm, experimental/calculated, compared to the calculated values (2.31–2.53 ppm) of the OH4 *t* conformers, Table 2). With axial fluorination at the C2 position as in **4**, the OH4 *trans* conformation is more favored than in **5**, the corresponding total population reaching 94%, and with a methoxy group at C2, as in **6**, almost 99% of the conformations display an OH4 *trans* orientation. Hence, the increase of the individual ^3^*J*_OH4-H_ coupling constants for these *trans* conformations does not solely originate from the perturbation of OH4-O5 IMHB by C2-substituents (taking the OH4 away from O5), but also from the higher population of the OH4-*trans* conformers as C2 is substituted by F and OMe.

Finally, it was investigated whether the levoglucosan pyranosyl ring featured differences in conformation when different substituents are present, which could be a factor influencing the IMHB features and vice versa. However, overlays of the equivalent conformers of compounds **4**, **5**, and **6** (see Supporting Information) show that root mean square deviations computed for the superposition are not higher than 0.013 Å (considering either the pyranosyl or the five-membered C6-bridge ring as reference). This shows that the substituent on the C2 atom does not significantly affect the levoglucosan scaffold when the two IMHB interactions (OH4···O2 and OH3···O5) occur.

#### 3.1.2. Interplay between OH3 Features and IMHB Involving OH4

Interestingly, for **4**–**6**, the conformation of the OH3 group, which is not involved in the OH4-O5 IMHB, has an indirect but significant influence on the shortening of the d_OH4···O5_ distance when the OH3 group is *gauche* (*t_g* and *t_g-*) compared to the *trans* (*t_t*). This leads to a higher calculated δ_OH4_ chemical shift, but has no significant influence on the dihedral angle or on the ^3^*J*_H4-OH_ value. This effect affects the IMHB features. In **4**, when the OH3 group is *trans*, the OH4-O5 interaction is weaker (longer d_OH4···O5_) compared to the *gauche* OH3 conformers, which thus leads to an increase of the ϕ_HC4OH_ value, favoring the OH···F interaction. Indeed, this is consistent with a reduction of the inductive effect of the OH3 group when it is acting as an H-bond donor, leading to a more electron-rich fluorine atom. In tandem, the O6 electron withdrawing effect is strengthened with the OH3···O6 IMHB, resulting in a more electron poor O5 atom. Additionally, for **6**, the OH3 conformation has a significant impact on the bond distances between OH4 and O5 or O2 (Table 2): the two most stable OH4 conformers (*t*_*t*_*g* and *t*_*t*_*g-*, amounting to 72%) in which OH3 has an IMHB with O6, show a shorter d_OH4···OMe_ distance than the d_OH4···O5_ one, leading to negative ϕ_HC4OH_ dihedral angles (−176.4° and −174.1°, respectively). In the other OH4 IMHB conformers, where the OH3 group is *gauche*, the loss of the OH3···O6 interaction favors the OH4···O5 IMHB. This is obviously to the detriment of the OH4···OMe IMHB, and positive ϕ_HC4OH_ dihedral angles are measured in this situation (from 176.3° to 178.6°). It is worth noticing that the effect on the individual ^3^*J*_OH4-H_ coupling constants are not so important, and that from **4** to **6**, these values are almost the same indicating that the differences observed are especially significantly dependent on the population of the OH4 *gauche* conformers.

The observations above are corroborated by the increase of the charge transfer for a given compound upon the loss of the OH3···O6 IMHB, reaching, for **5** and **4**, the values calculated for **7** and **8** for which no OH3···O6 IMHB can occur. Hence, the NBO analysis confirms that the occurrence of the OH3···O6 IMHB interaction favors the OH4···F2 interaction. Indeed, in the *t_t* form of **4**, an enhanced *E*^(2)^_n→σ*_ value for OH4···F2 and a lowered *E*^(2)^_n→σ*_ value for OH4···O5 is calculated with respect to the *t_g* conformer. This directly affects the individual δ_OH_ chemical shifts identified to be significantly dependent of the three-center IMHB interaction. Indeed, δ_OH4_ decreases from the *t_g* to the *t_t* conformer of **4**, since the contribution OH4···O5 (*E*^(2)^_n→σ*_ value) is larger than OH4···F2. In **6**, the main contribution is observed for OH4···OMe rather than for OH4···O5, and it therefore results to an increase of δ_OH4_ in its *t_g* forms compared to its *t_t* conformers.

### 3.2. Description of the Intramolecular OH3 Interactions

**Figure 5:**
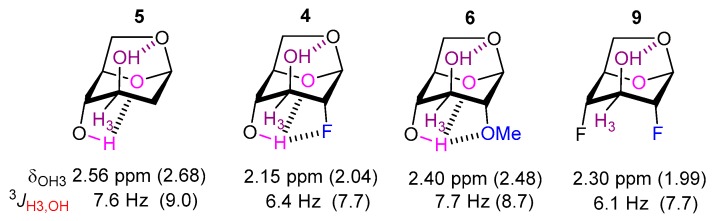
NMR features of the OH3 surroundings in compounds **4**–**6**, and **9**. The theoretical data are given in parenthesis.

The axial OH3 groups of **4**–**6**, **9** are engaged in IMHB with O6, with optimum ϕ_HC3OH_ dihedral angles for the IMHB conformations of around 157°–160°. As a consequence, the experimental coupling constants involving OH3, ^3^*J*_OH3-H_ (see Table 1 and Figure 5), are significantly lower than the OH4 hydroxyl group ones, with values around 7.6 Hz for compounds **5** and **6**. Interestingly, for the two deoxyfluorinated derivatives **4** (6.4 Hz) and **9** (6.1 Hz), a further significant decrease of ^3^*J*_OH3-H_ is observed despite ϕ_HC3OH_ dihedral angles very similar to **5** and **6**. Given the OH3···O6 interaction motif corresponds to a 6-membered IMHB ring, within the AIM methodology, a BCP is systematically detected along this interaction for all these compounds (Table 4). The corresponding electron densities (from 0.018 to 0.022 e∙bohr^−3^) and *E*_HB_ parameters (from 18 to 23 kJ∙mol^−1^) are much higher than for the OH4···OMe case, and vary in the order of **6** > **5** > **4** > **9**. In line with these trends, the IMHB distances involving OH3 decrease following the same order, and the δ_OH3_ chemical shift values of the respective IMHB conformers increase rather similarly (**5** > **6** > **4** > **9**, Table 3).

Both observations regarding ^3^*J*_OH3-H_ and δ_OH3_ variations are difficult to explain by using IMHB arguments only. In contrast, conformational analysis revealed that the IMHB conformations of OH3 are less stabilized than the IMHB conformations involving OH4, despite the apparent stronger and shorter IM H-bonds with OH3 (less than 2.2 Å, to be compared to more than 2.3 Å for OH4 IMHBs). Thus, it was found that conformers with OH3···O6 IMHB are significantly less populated (53%, 73%, and 51% in **4**, **6**, and **9,** respectively, and 78% in **5**, in comparison with populations larger than 83% for IMHB conformers involving OH4). The same applies for the δ_OH3_ chemical shifts: the values computed for OH3 for the IMHB conformers (Table 3) are always larger than the equivalent δ_OH4_ ones (Table 2), in agreement with the greater deshielding of OH3 because of the stronger interaction. However, since the OH3 IMHB conformers are less stabilized and therefore less represented in the whole set, the average δ_OH3_ values obtained after weighting of the individual values do appear significantly lower than the δ_OH4_ values, except for **5**. Hence, it is again shown that careful consideration of computational data is required to explain the experimental data.

## 4. Materials and Methods

### 4.1. NMR Data Acquisition

NMR data were collected on a Bruker AVIII HD 500 MHz NMR spectrometer (Bruker UK Ltd., Coventry, UK). Samples were shimmed until the w_½_ for the residual CDCl_3_ solvent signal was 0.5 Hz or better through a combination of sequential iterations of “TopShim” gradient shimming with additional manual intervention, as required. ^1^H spectra were collected with TD = 65,536 points and SW = 14 ppm (o1p = 5.0 ppm); zero-filling afforded digital resolution of 0.05 Hz/point. ^19^F spectra were collected with TD = 262,144 points and SW = 50 ppm (o1p proximal to ^19^F signal); zero-filling afforded digital resolution of 0.09 Hz/point. ^19^F{^1^H} spectra were collected with TD = 262,144 points and SW = 200 ppm (o1p proximal to ^19^F signal; inverse-gated decoupling with o2p = 5.0 ppm); zero-filling afforded digital resolution of 0.36 Hz/point. ^1^H{^19^F} spectra were collected with TD = 65,536 points and SW = 14 ppm (o1p = 5.0 ppm; adiabatic inverse-gated decoupling with o2p proximal to ^19^F signal); zero-filling afforded digital resolution of 0.05 Hz/point. For compound **4**, residual molecular sieve particles affected shimming leading to poorer lineshape and resolution (w_½_ for residual CDCl_3_ solvent signal was 1.1 Hz).

### 4.2. Quantum Calculations

All DFT calculations were performed by using the D.01 version of the Gaussian 09 program [27]. We have previously shown, for a series of acyclic fluorohydrins [8], that the experimental trends regarding the variation of ^h1^*J*_OH···F_ and ^3^*J*_OH-H_ can be properly reproduced through quantum calculations using optimized geometries at the PCM/MPWB1K/6-31+G(d,p) level and coupling constants calculated at the B97-2/pcJ-2 level of theory.

Unfortunately, within the current series of levoglucosan derivatives, this methodology appeared to fail and we have therefore selected the following methodology. The conformational landscapes of the levoglucosans have been exhaustively explored at the B97-D3^BJ^/6-311++G(2d,p) level of theory with investigations around the dihedral angles of the hydroxyl and methoxy groups. This level of theory is, therefore, based, on one hand, on a more extended basis set and, on the other hand, on a dispersion-corrected density functional, B97-D3^BJ^, designed for a more accurate description of systems involving noncovalent interactions with dispersion effects. Solvent effects (CHCl_3_) were systematically introduced by means of the polarizable continuum model (PCM) within the integral equation formalism. Frequency calculation of the various energetic minima were then carried out at the same level of theory, allowing the calculations of the corresponding Gibbs free energies, and of the Boltzmann distribution (Equation (1)):

(1) $$p_{i}=\frac{e^{-\Delta G_{i}/RT}}{\sum_{i}e^{-\Delta G_{i}/RT}},$$

The spin-spin coupling constants (*J*) were then estimated from the previous optimized geometries by using the gauge-invariant atomic orbital (GIAO) method. The hybrid B97-2 functional [28] and the pcJ-2 basis set, specifically designed for the calculation of these NMR parameters [29], were used. Again, solvent (CHCl_3_) effects were introduced through the PCM model. Weighted *J* values were estimated according to their relative populations in CHCl_3_ at 298 K and using Equation (2). In addition, the chemical shifts of the hydroxyl hydrogen atoms were computed at the same level of theory and the weighted values were calculated similarly to the coupling constants:

(2) $$J=\sum_{i}p_{i}\;J_{\left(i\right)},$$

IMHB interactions were analyzed through AIM topological analysis of the IEF-PCM/B97-D3^BJ^/6-311++G(2d,p) wavefunctions with the AIM2000 program [30]. In addition to the electron densities ρ_b_ and their Laplacians, the potential energy density *V*_b_ at the BCP were used to gain additional insights into the strength of a given HB [8,25,31,32,33,34]. Indeed, the HB energy can be estimated by using *V*_b_ according to the established relationship in Equation (3) [35]:

(3) *E*_HB_ = ½ *V*_b_,

The complementary contribution of charge transfer between the acceptor lone pair(s) of electrons and the σ* donor antibonding orbital was estimated through natural bond orbital (NBO) analyses [36]. The NBO method was applied at the IEF-PCM/B97-D3^BJ^/6-311++G(2d,p) level in CHCl_3_ to provide the corresponding interaction energies (*E*^(2)^_n→σ*_) evaluated from second-order perturbation theory, using the NBO 6.0 program [37].

## 5. Conclusions

Intramolecular hydrogen bond features involving OH3 and OH4 of a range of levoglucosan analogues were obtained by both experimental (NMR) and theoretical (DFT, AIM, NBO) means. The axial OH4 group was shown to hydrogen bond with O5, and this IMHB is perturbed by axial substituents at C2: with competing H-bond acceptors (F, OMe) at C2, OH4 engages in a three-center interaction, which can be deduced from the experimentally obtained ^3^*J*_OH4-H_ coupling constants and interpreted following the rationales introduced by Bernet et al. The axial OH3 is involved in IMHB with O6, and while varying substitution at C2 and C4 does not seem to modify the conformation of the six-membered IMHB (as calculated by the very similar dihedral angles), the corresponding ^3^*J*_OH3-H_ coupling constants do vary significantly. Conformational analysis has shown that, contrary to intuition for such rigid systems, there can be significant rotation around the C-OH bond, leading to non-hydrogen bonded conformations being significantly populated. The resulting averaging of the observed coupling constant and chemical shift values therefore hamper straightforward interpretation. The computational study allows to demonstrate that OH3···O6 IMHB are stronger and shorter than OH4···O5 IMHB, despite the clear preference of OH4 for IM H-bonding compared to OH3, (populations greater than 83% for OH4 and range of 50% to 77% for OH3). Furthermore, the theoretical data allow emphasizing the contribution of the IMHB populations to the coupling constant and chemical shift variations. Finally, the present work brings clear evidences on the subtle influence of IMHB on hydroxyl group features, both in terms of geometry and electron density parameters. Interestingly, while a ^h1^*J*_OH4···F_ value was measured for both fluorinated substrates, the calculated OH···F distance was larger than the sum of their van der Waals radii.

Our results complement and augment the conclusions by Bernet et al., that the weak hydrogen-bond accepting fluorine atom is able to perturb OH···O IMHBs, even for fluorination at the levoglucosan C2 position, and that additional substitution modifies this perturbation. However, further to the work of Bernet et al. we show that even for the nominally conformationally rigid levoglucosan systems, conformational analysis and weighting of the theoretical data is important to accurately describe IM H-bonding features. More precisely, weighted coupling constants and chemical shifts are necessary to fully analyze the NMR experimental trends.

## Figures and Tables

**Table 1:** Experimental and theoretical NMR features of the OH4 and OH3 hydroxyl groups in compounds **4**–**9**.

Compound	^3^*J*_H4,OH_ (Hz)		δ_OH4_ (ppm)		^h1^*J*_OH···F_ (Hz)		^3^*J*_H3,OH_ (Hz)		δ_OH3_ (ppm)	
	Exp	Calc ^1^	Exp	Calc ^1^	Exp	Calc ^1^	Exp	Calc ^1^	Exp	Calc ^1^
**5**	9.5	12.0	2.29	2.20	-	-	7.5	9.0	2.56	2.68
**4**	10.9	12.9	2.58	2.46	1.4	0.9	6.4	7.7	2.15	2.04
**6**	11.6	13.2	2.88	2.77	v	-	7.7	8.9	2.40	2.48
**7**	9.9	10.7	2.39	2.29	-	-	-	-	-	-
**8**	11.3	12.6	2.60	2.50	0.8	0.3	-	-	-	-
**9**	-	-	-	-	-	-	6.1	7.7	2.30	1.99

^1^ Theoretical parameters are calculated at the IEF-PCM/B97–2/pcJ-2//B97-D3^BJ^/6-311++G(2d,p) level of theory in CHCl_3_ at 25 °C.

**Table 2:** Theoretical features ^1^ of the levoglucosan conformations featuring the OH4 group in compounds **4**–**8**.

Conformer ^2^		*G* (kJ∙mol^−1^)	*p_i_* (%)	*d*_OH4···O5_ (Å)	*d*_OH4···X2(ax)_ (Å)	_HO4CH_ (°)	^3^*J*_OH4-H_ (Hz)	δ_OH4_ (ppm)
**5**	*t_t*	0.0	65.2%	2.377	-	165.0	13.6	2.31
	*t_g*	3.8	14.0%	2.324	-	164.2	13.5	2.49
	*t_g-*	5.4	7.5%	2.319	-	163.7	13.2	2.53
	*g-_t*	5.5	7.0%	-	-	−56.7	3.0	1.31
	*g_t*	6.1	5.5%	-	-	68.7	0.8	1.05
	*g_g-*	12.2	0.5%	-	-	76.0	−0.2	1.05
	*g-_g-*	13.4	0.3%	-	-	−60.2	2.8	1.24
**4**	*t_t*	0.0	49.8%	2.465	2.595	175.0	13.8	2.49
	*t_g*	1.8	23.7%	2.388	2.802	170.8	13.7	2.62
	*t_g-*	2.2	20.2%	2.386	2.782	170.7	13.4	2.61
	*g-_t*	8.4	1.7%	-	-	−60.1	1.7	1.34
	*g_t*	8.8	1.4%	-	-	65.3	0.9	1.08
	*g_g*	9.5	1.1%	-	-	67.5	0.6	1.04
	*g-_g*	10.1	0.9%	-	-	−57.1	2.2	1.36
	*g_g-*	10.2	0.8%	-	-	−54.5	−0.1	1.07
	*g-_g-*	11.5	0.5%	-	-	−58.2	1.2	1.29
**6**	*t_t_g*	0.0	48.1%	2.511	2.384	−176.4	13.4	2.83
	*t_t_g-*	1.8	23.7%	2.539	2.324	−174.1	13.3	2.88
	*t_g_g*	4.1	9.2%	2.408	2.627	176.3	13.6	2.66
	*t_g-_g*	4.5	7.8%	2.413	2.603	177.1	13.4	2.69
	*t_g_g-*	5.5	5.2%	2.431	2.578	178.6	13.7	2.65
	*t_g-_g-*	5.7	4.8%	2.425	2.588	178.0	13.4	2.65
	*g-_t_g*	11.4	0.5%	-	-	−64.1	1.1	1.14
	*g-_t_g-*	12.8	0.3%	-	-	−67.2	0.7	1.10
	*g_t_g-*	12.9	0.3%	-	-	67.0	0.7	0.97
	*g_t_g*	13.3	0.2%	-	-	67.1	0.6	0.93
**7**	*t*	0.0	82.9%	2.321	-	163.4	12.6	2.50
	*g*	5.2	10.0%	-	-	77.9	−0.3	1.11
	*g-*	6.1	7.1%	-	-	−49.3	3.4	1.49
**8**	*t*	0.0	92.6%	2.390	2.840	169.5	13.5	2.60
	*g*	7.6	4.3%	-	-	68.9	0.6	1.18
	*g-*	8.4	3.1%	-	-	−52.6	2.9	1.54

^1^ Theoretical parameters are calculated at the IEF-PCM/B97-D3^BJ^/6-311++G(2d,p) level of theory in CHCl_3_ at 25 °C. ^2^ The first descriptor refers to H-C4-O4-H, the second descriptor to H-C3-O3-H, and the last descriptor to the H-C2-O2-CH_3_ torsion angle.

**Table 3:** Theoretical features ^1^ of the levoglucosan conformations featuring the OH3 group in compounds **4**–**6**, **9**.

Conformer ^2^		*G* (kJ∙mol^−1^)	*p_i_* (%)	*d*_OH3···O6_ (Å)	_HO3CH_ (°)	^3^*J*_OH3-H_ (Hz)	δ_OH3_ (ppm)
**5**	*t_t*	0.0	65.2%	2.122	156.9	11.0	3.15
	*t_g*	3.8	14.0%	-	81.6	−0.2	0.74
	*t_g-*	5.4	7.5%	-	−50.8	3.9	1.33
	*g-_t*	5.5	7.0%	2.109	158.2	11.1	3.34
	*g_t*	6.1	5.5%	2.116	158.2	11.3	3.26
	*g_g-*	12.2	0.5%	-	76.0	4.0	1.21
	*g-_g-*	13.4	0.3%	-	−60.2	3.5	1.20
**4**	*t_t*	0.0	49.8%	2.136	158.5	12.0	2.66
	*t_g*	1.8	23.7%	-	60.4	1.7	1.20
	*t_g-*	2.2	20.2%	-	−50.1	4.1	1.56
	*g-_t*	8.4	1.7%	2.169	159.8	12.2	2.59
	*g_t*	8.8	1.4%	2.185	160.6	12.6	2.47
	*g_g*	9.5	1.1%	-	62.2	1.5	1.14
	*g-_g*	10.1	0.9%	-	64.4	1.0	1.15
	*g_g-*	10.2	0.8%	-	−54.5	3.2	1.39
	*g-_g-*	11.5	0.5%	-	−58.2	2.6	1.36
**6**	*t_t_g*	0.0	48.1%	2.094	158.4	11.5	2.97
	*t_t_g-*	1.8	23.7%	2.078	157.6	11.7	3.07
	*t_g_g*	4.1	9.2%	-	77.1	−0.3	0.77
	*t_g-_g*	4.5	7.8%	-	−52.2	3.2	1.35
	*t_g_g-*	5.5	5.2%	-	68.8	0.5	0.98
	*t_g-_g-*	5.7	4.8%	-	−51.8	3.4	1.34
	*g-_t_g*	11.4	0.5%	2.173	159.7	11.8	2.64
	*g-_t_g-*	12.8	0.3%	2.138	159.0	11.9	2.80
	*g_t_g*	12.9	0.3%	2.173	160.0	12.0	2.59
	*g_t_g-*	13.3	0.2%	2.157	159.8	12.3	2.66
**9**	*t*	0.0	51.0%	2.186	160.3	12.4	2.50
	*g-*	1.8	24.9%	-	−48.8	4.3	1.64
	*g*	1.9	24.1%	-	63.4	1.5	1.28

^1^ Theoretical parameters are calculated at the IEF-PCM/B97-D3^BJ^/6-311++G(2d,p) level of theory in CHCl_3_ at 25 °C. ^2^ The first descriptor refers to H-C4-O4-H, the second descriptor to H-C3-O3-H, and the last descriptor to H-C2-O2-CH_3_ torsion angle.

**Table 4:** AIM and NBO computed descriptors ^1^ of the IMHB conformers of **4**–**9**.

Conformer		*E*^(2)^_n→σ*_ n_O6_ → σ*_OH3_ (kJ∙mol^−1^)	ρ_OH3···O6_ e∙bohr^−3^	*E*_HB_ (kJ∙mol^−1^)	*E*^(2)^_n→σ*_ n_O5_ → σ*_OH4_ (kJ∙mol^−1^)	*E*^(2)^_n→σ*_ n_X2_ → σ*_OH4_ (kJ∙mol^−1^)	ρ_OH4···OMe_ e∙bohr^−3^	*E*_HB_ (kJ∙mol^−1^)
**4**	*t_t*	11.9	0.020	20.0	1.6	0.4	-	-
	*t_g*	-	-	-	2.4	0.2	-	-
	*t_g-*	-	-	-	2.4	0.3	-	-
	*g-_t*	10.5	0.018	18.5	-	-	-	-
	*g_t*	9.7	0.018	17.9	-	-	-	-
**5**	*t_t*	12.6	0.020	20.5	2.8	-	-	-
	*t_g*	-	-	-	3.5	-	-	-
	*t_g-*	-	-	-	3.6	-	-	-
	*g-_t*	13.6	0.021	21.1	-	-	-	-
	*g_t*	13.1	0.020	20.7	-	-	-	-
**6**	*t_t_g*	14.1	0.021	22.1	1.2	3.8	0.011	10.5
	*t_t_g-*	15.0	0.022	23.0	1.0	4.2	0.012	11.4
	*t_g_g*	-	-	-	2.1	0.8	0.007	7.0
	*t_g-_g*	-	-	-	2.1	1.0	0.008	7.2
	*t_g_g-*	-	-	-	1.9	1.1	0.008	7.2
	*t_g-_g-*	-	-	-	2.0	1.1	0.007	7.1
**7**	*t*	-	-	-	2.4	0.08	-	-
**8**	*t*	-	-	-	3.6	-	-	-
**9**	*t*	9.7	0.018	17.9	-	-	-	-

^1^ Theoretical parameters are calculated at the IEF-PCM/B97-D3^BJ^/6-311++G(2d,p) level of theory in CHCl_3_ at 25 °C.

## Supplementary Materials

The synthesis, characterization and computational data are available online.
