# Supplementary material for: A Study of Intramolecular Hydrogen Bonding in Levoglucosan Derivatives
Source: Molecules. 2017 Mar 24;22(4):518. doi: 10.3390/molecules22040518 (PMC6154293; doi:10.3390/molecules22040518)
Supplement: Supplementary file 1 [file molecules-22-00518-s001.pdf]

Table of Contents

1 Synthesis and/or characterisation data....3
1.1 Characterisation data of 1,6-Anhydro-2-deoxy-2-fluoro-β-D-glucopyranoside (4)....3
1.2 Characterisation data of 1,6-anhydro-2-deoxy-β-D-arabino-hexopyranoside (5)....3
1.3 Synthesis and characterisation data of 1,6-Anhydro-2-*O*-methyl-β-D-glucopyranoside (6)....4
1.4 Synthesis and characterisation data 1,6-anhydro-2,3-dideoxy-3-fluoro-β-D-arabino-hexopyranoside (7)....5
1.5 Synthesis and characterisation data of 1,6-anhydro-2,3-dideoxy-2,3-difluoro-β-D-glucopyranoside (8)....5
1.6 Characterisation data of 1,6-anhydro-2,4-dideoxy-2,4-difluoro-β-D-glucopyranoside (9)....6
2 Spectral details for OH coupling constants....7
2.1 Sample preparation....7
2.2 Compound 4....8
2.2.1 Spectrum in $CDCl_3$ with <1% H_2O....8
2.2.2 Spectrum in $CDCl_3$ with 1 equiv of H_2O....10
2.3 Compound 5....11
2.4 Compound 6....12
2.5 Compound 7....13
2.6 Compound 8....14
2.7 Compound 9....16
3 Computational data....17
3.1 Compound 4....17
3.2 Compound 5....18
3.3 Compound 6....19
3.4 Compound 7....19
3.5 Compound 8....20
3.6 Compound 9....20
4 ^{1}H, ^{19}F and ^{13}C spectra of all compounds....22
4.1 Compound 4....22
4.2 Compound 5....28

4.3 Compound 6 .... **31**
4.4 Compound 7 .... **34**
4.5 Compound 8 .... **38**
4.6 Compound 9 .... **44**
4.7 Compound 10 .... **48**

1 Synthesis and/or characterisation data

1.1 Characterisation data of 1,6-Anhydro-2-deoxy-2-fluoro-β-D-glucopyranoside (4)

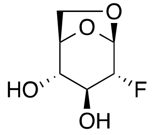

4

Mp 118-120°C (hexane/acetone), lit 129-130 °C (ether/acetone).[1] **[α]$_D$** -79.2 (c 1.20, acetone, 21 °C), lit -75 (c 1.20, MeOH, 20 °C).[2] **^{1}H NMR** (500 MHz, $CDCl_3$) δ 5.60 (1H, br. q, *J* 1.8 Hz, H_1), 4.63 (1H, m, *J* 5.4 Hz, H_5), 4.34 (1H, dq, *J* 45.2, 1.7 Hz, H_2), 4.21 (1H, dd, *J* 7.6, 0.9 Hz, H_{6endo}), 4.02 (1H, ddquin, *J* 15.7, 6.5, 1.8 Hz, H_3), 3.83 (1H, ddd appears as m, *J* 7.3, 5.8, 1.2 Hz, H_{6exo}), 3.66 (1H, br. dq, *J* 10.9, 1.7 Hz, H_4), 2.60 (1H, dd, *J* 11.0, 1.4 Hz, OH_4), 2.16 (1H, br. dd, *J* 6.4, 0.6 Hz, OH_3) ppm. **^{1}H[^{19}F] NMR** (500 MHz, $CDCl_3$) δ 5.58 (1H, br. t, *J* 1.6 Hz, H_1), 4.62 (1H, m, *J* 5.3 Hz, H_5), 4.32 (1H, q, *J* 1.5 Hz, H_2), 4.19 (1H, dd, *J* 7.7, 0.9 Hz, H_{6endo}), 4.00 (1H, dquin, *J* 6.4, 1.9 Hz, H_3), 3.81 (1H, dd, *J* 7.7, 5.6 Hz, H_{6exo}), 3.65 (1H, br. dq, *J* 10.9, 1.9 Hz, H_4), 2.58 (1H, d, *J* 10.9 Hz, OH_4), 2.15 (1H, d, *J* 6.4 Hz, OH_3) ppm. **^{13}C NMR** (101 MHz, Acetone-*d6*) δ 99.29 (1C, d, *J* 30.1 Hz, C_1), 90.23 (1C, d, *J* 179.0 Hz, C_2), 76.95 (1C, s, C_5), 71.88 (1C, d, *J* 26.4 Hz, C_3), 71.61 (1C, d, *J* 5.1 Hz, C_4), 65.31 (1C, s, C_6) ppm. **^{19}F NMR** (471 MHz, $CDCl_3$) δ -187.49 (1F, br. ddd, *J* 45.2, 15.7, 1.3 Hz) ppm. **MS** (ESI$^-$) (m/z) 209.8 $[M+HCO_2]^-$ (100%). NMR data match those previously reported.[2]

1.2 Characterisation data of 1,6-anhydro-2-deoxy-β-D-arabino-hexopyranoside (5)

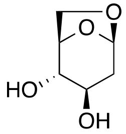

5

To a solution of compound **10**[3] (153 mg, 0.65 mmol, 1 equiv) in methanol (0.5 mL) was added $Pd(OH)_2/C$ (20%, 12 mg, 0.017 mmol, 0.03 equiv) and the resulting solution degassed with H_2. Stirring under H_2 atmosphere was continued at rt for 12h before the mixture was filtered over a pad of Celite and concentrated under vacuum. Purification by column chromatography (petroleum ether/ethyl acetone 60:40) afforded 83 mg (0.58 mmol, 89%) of compound **5** as a translucent gel. **Rf** 0.12 (petroleum ether/acetone 60:40). **[α]$_D$** -120.3 (c 1.10, water, 21 °C), lit -119 (c 1.10, water, 20 °C).[4] **^{1}H NMR** (500MHz, $CDCl_3$): δ 5.65

(1H, br. d, *J* 1.4 Hz, H_1), 4.56 (1H, m, *J* 5.4 Hz, H_5), 4.36 (1H, ddd, *J* 7.6, 0.8, 0.2 Hz, H_{6endo}), 3.85-3.91 (1H, ddquin appears as m, *J* 7.4, 5.2, 1.6 Hz, H_3), 3.80 (1H, dd, *J* 7.6, 5.4 Hz, H_{6exo}), 3.76 (1H, br. dq, *J* 9.5, 1.6 Hz, H_4), 2.57 (1H, d, *J* 7.5 Hz, OH_3), 2.31 (1H, d, *J* 9.5 Hz, OH_4), 2.18 (1H, ddd, *J* 15.3, 5.2, 1.5 Hz, H_{2ax}), 1.86 (1H, m, *J* 15.3, 2.0, 1.2 Hz, H_{2eq}) ppm. **^{13}C NMR** (101 MHz, acetone-*d6*) δ 100.09 (1C, s, C_1), 76.22 (1C, s, C_5), 72.13 (1C, s, C_4), 68.26 (1C, s, C_3), 64.38 (1C, s, C_6), 35.43 (1C, s, C_2) ppm. NMR data haven't been reported in $CDCl_3$.

1.3 Synthesis and characterisation data of 1,6-Anhydro-2-*O*-methyl-β-D-glucopyranoside (**6**)

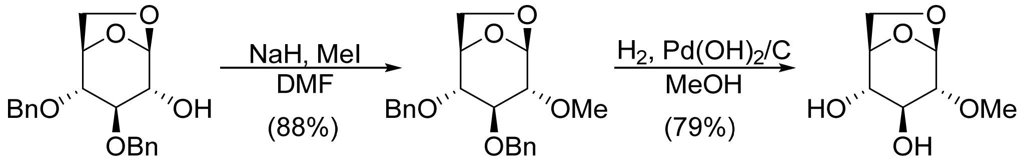

11 **11_OMe** **6**

To a stirred solution of compound **11**[5] (645 mg, 1.88 mmol, 1 equiv) in DMF (12.3 mL) was added sodium hydride in mineral oil (60%, 183 mg, 4.57 mmol, 2.4 equiv). After 30 minutes at rt, the resulting solution was cooled at 0 °C and MeI added (0.48 mL, 7.71 mmol, 4.1 equiv). The reaction mixture was then allowed to reach rt and the stirring continued for 12 H. After quenching with methanol (1.2 mL), the solution was concentrated to a syrup before being dissolved in DCM (25 mL). The resulting organic layer was then washed with water (2x25 mL), dried over $MgSO_4$ and the solvent removed under vaccum to afford a crude mixture. Purification by column chromatography (petroleum ether/acetone 90:10 to 70:30) afforded 591 mg (1.66 mmol, 88%) of compound **11_OMe** as a translucent oil. **Rf** 0.55 (petroleum ether/acetone 70:30). **[α]$_D$** -19.0 (c 1.00, $CHCl_3$, 22 °C). **^{1}H NMR** (500 MHz, $CDCl_3$) δ 7.28-7.42 (10H, m, H_{Ar}), 5.50 (1H, br. s, H_1), 4.63 (1H, d, *J* 12.6 Hz, H_{Benz}), 4.57-4.59 (1H, m, H_5), 4.58 (1H, d appears as m, *J* 12.6 Hz, H_{Benz}), 4.57 (1H, d appears as m, *J* 12.1 Hz, H_{Benz}), 4.54 (1H, d, *J* 12.1 Hz, H_{Benz}), 3.93 (1H, dd, *J* 7.2, 1.0 Hz, H_{6endo}), 3.70 (1H, dd, *J* 7.1, 5.8 Hz, H_{6exo}), 3.58 (1H, tt, *J* 2.3, 1.2 Hz, H_3), 3.39 (3H, s, H_{Me}), 3.34-3.37 (1H, m, H_4), 3.16-3.18 (1H, m, H_2) ppm. **^{13}C NMR** (101 MHz, $CDCl_3$) δ 137.93 (1C, s, C_{Ar}), 137.91 (1C, s, C_{Ar}), 128.52 (2C, s, C_{Ar}), 128.49 (2C, s, C_{Ar}), 127.82-127.98 (4C, m, C_{Ar}), 127.73 (2C, s, C_{Ar}), 100.08 (1C, s, C_1), 79.11 (1C, s, C_2), 76.51 (1C, s, C_4), 75.74 (1C, s, C_3), 74.38 (1C, s, C_5), 72.05 (1C, s, C_7), 71.22 (1C, s, C_7), 65.45 (1C, s, C_6), 57.79 (1C, s, C_{Me}) ppm. **MS** (ESI^+) (m/z): 357.3 $[M+H]^+$(9%), 379.3 $[M+Na]^+$(84%), 735.6 $[2M+Na]^+$(100%).

To a solution of compound **11_OMe** (481 mg, 1.35 mmol, 1 equiv) in methanol (1.1 mL) was added $Pd(OH)_2/C$ (20%, 20 mg, 0.054 mmol, 0.04 equiv) and the resulting solution degassed with H_2. Stirring under H_2 atmosphere was continued at rt for 12h before the mixture was filtered over a pad of Celite and concentrated under vacuum. Purification by column chromatography (petroleum ether/acetone 60:40) afforded 188 mg (1.07 mmol, 92%) of compound **6** as a translucent crystalline solid. **Rf** 0.11 (petroleum ether/acetone 60:40). **Mp** 88-90°C (petroleum ether/acetone), lit 93-94 °C (hexane/acetone).[6] **[α]$_D$** -73.4 (c 1.45, acetone, 21 °C), lit -73.4 (c 1.45, acetone, 21 °C).[6] **^{1}H NMR** (500 MHz, $CDCl_3$) δ 5.58 (1H, br. t, *J* 1.8 Hz, H_1), 4.59 (1H, m, *J* 5.3 Hz, H_5), 4.22 (1H, br. dd, *J* 7.7, 0.4 Hz, H_{6endo}), 3.90 (1H, dquin, *J* 7.7, 1.9 Hz, H_3), 3.80 (1H, dd, *J* 7.6, 5.3 Hz, H_{6exo}), 3.65 (1H, br. dq, *J* 11.6, 1.9 Hz, H_4), 3.51 (3H, s, H_{Me}), 3.20 (1H, br. q, *J* 1.7 Hz, H_2), 2.88 (1H, d, *J* 11.7 Hz, OH_4), 2.40 (1H, d, *J* 7.7 Hz, OH_3) ppm. **^{13}C NMR** (101 MHz, $CDCl_3$) δ 100.28 (1C, s, C_1), 79.08 (1C, s, C_2), 76.79 (1C, s, C_5), 70.92 (1C, s,

C_4), 69.87 (1C, s, C_3), 65.38 (1C, s, C_6), 58.05 (1C, s, C_{Me}) ppm. **MS** (ESI$^+$) (m/z) 177.2 $[M+H]^+$(13%), 199.1 $[M+Na]^+$(100%). HRMS (ESI+) $C_7H_{12}NaO_5$ $[M+Na]^+$ calcd. 199.0582, found. 199.0577; $C_{14}H_{24}NaO_{10}$ $[2M+Na]^+$ calcd. 375.1267, found. 375.1257. NMR data haven't been reported in $CDCl_3$.

1.4 Synthesis and characterisation data 1,6-anhydro-2,3-dideoxy-3-fluoro-β-D-arabino-hexopyranoside (**7**)

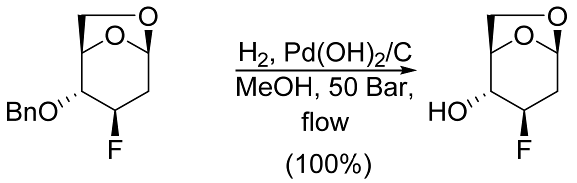

12 **7**

Compound **12**[3] (102 mg, 0.43 mmol) was dissolved in MeOH (1 mL) and pumped at a flow rate of 1.0 mL/min through a 20% $Pd(OH)_2$ cartridge installed in the H-Cube® flow hydrogenation reactor. The solution of starting material was passed through the system a single time under 50 bar of H_2 at rt before being flushed out with MeOH (10 mL). Evaporation of the solvent under vacuum afforded 64 mg (0.43 mmol, 100%) of compound **7** as a translucent oil. **Rf** 0.26 (petroleum ether/acetone 70:30). **[α]$_D$** -101.6 (c 0.25, $CHCl_3$, 19 °C). **^{1}H NMR** (500 MHz, $CDCl_3$) δ 5.57 (1H, br. d, *J* 1.1 Hz, H_1), 4.67 (1H, m, *J* 46.7 Hz, H_3), 4.55 (1H, m, *J* 6.0 Hz, H_5), 4.22 (1H, dt, *J* 7.6, 1.1 Hz, H_{6endo}), 3.84 (1H, ddd appears as m, *J* 7.6, 6.0, 4.0 Hz, H_{6exo}), 3.81 (1H, m, *J* 10.0, 2.0, 0.9 Hz, H_4), 2.38 (1H, d, *J* 9.9 Hz, OH_4), 2.10 (1H, dddd appears as m, *J* 38.5, 15.8, 4.6, 1.9 Hz, H_{2ax}), 2.04 (1H, m, *J* 23.3, 15.8 Hz, H_{2eq}) ppm. **^{1}H[^{19}F] NMR** (500 MHz, $CDCl_3$) δ 5.57 (1H, br. d, *J* 1.3 Hz, H_1), 4.67 (1H, ddt, *J* 4.7, 3.3, 1.5 Hz, H_3), 4.55 (1H, m, *J* 6.0 Hz, H_5), 4.22 (1H, dd, *J* 7.6, 1.2 Hz, H_{6endo}), 3.84 (1H, dd appears as m, *J* 7.5, 6.0 Hz, H_{6exo}), 3.82 (1H, m, *J* 10.0 Hz, H_4), 2.38 (1H, d, *J* 10.0 Hz, OH_4), 2.10 (1H, ddd, *J* 15.8, 4.5, 1.9 Hz, H_{2ax}), 2.04 (1H, m, *J* 15.8, 1.5 Hz, H_{2eq}) ppm. **^{13}C NMR** (101 MHz, $CDCl_3$) δ 99.77 (1C, s, C_1), 88.12 (1C, d, *J*=177.1 Hz, C_3), 75.37 (1C, s, C_5), 68.62 (1C, d, *J* 26.4 Hz, C_4), 64.30 (1C, d, *J* 5.9 Hz, C_6), 33.26 (1C, d, *J* 20.5 Hz, C_2) ppm. **^{19}F NMR** (471 MHz, $CDCl_3$) δ -177.32 (1F, m, *J* 46.7, 38.5, 23.3, 10.8, 4.0 Hz) ppm. **MS** (EI) (m/z) 148.1 $[M]^{+\bullet}$(60%).

1.5 Synthesis and characterisation data of 1,6-anhydro-2,3-dideoxy-2,3-difluoro-β-D-glucopyranoside (**8**)

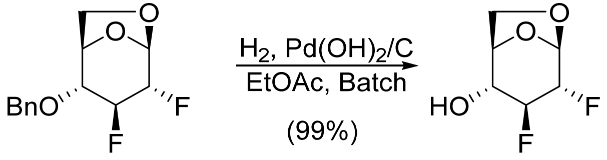

13 **8**

To a solution of compound **13**[3,7] (2.22 g, 8.66 mmol, 1 equiv) in methanol (1.1 mL) was added $Pd(OH)_2/C$ (20%, 300 mg, 0.43 mmol, 0.05 equiv) and the resulting solution degassed with H_2. Stirring under H_2 atmosphere was continued at rt for 12h before the mixture was filtered over a pad of Celite and concentrated under vacuum. Purification by column chromatography (petroleum ether/acetone 60:40) afforded 1.43 g (8.59 mmol, 99%) of compound **8** as a white solid. **Rf** 0.16 (petroleum ether/ethyl acetate 80:20). **Mp** 92-94 °C (chloroform). **[α]$_D$** -61.6 (c 0.7, $CHCl_3$, 22 °C), lit -59° (c 0.7, $CHCl_3$).[7] **^{1}H NMR** (500 MHz, $CDCl_3$) δ 5.58 (1H, br. q, *J* 1.8 Hz, H_1), 4.70 (1H, ddquin appears as m, *J* 43.2, 12.3, 1.7 Hz, H_3), 4.61 - 4.66 (1H, m, H_5), 4.43 (1H, ddqd, *J* 44.1, 12.4, 1.6, 0.6 Hz, H_2), 4.08 (1H, dt, *J* 7.8, 1.2 Hz, H_{6endo}), 3.86 (1H, br. ddt, *J* 7.8, 5.8, 1.8 Hz, H_{6exo}), 3.78 (1H, br. ddq, *J* 13.0, 11.3, 1.6 Hz, H_4), 2.58 (1H, dt, *J* 11.4, 0.8 Hz, OH_4) ppm. **^{1}H[^{19}F]** NMR (500 MHz, $CDCl_3$) δ 5.59 (1H, br. t, *J* 1.7 Hz, H_1), 4.72 (1H, quin, *J* 1.7 Hz, H_3), 4.65 (1H, m, *J* 5.8 Hz, H_5), 4.45 (1H, m, *J* 1.6 Hz, H_2), 4.10 (1H, ddd, *J* 7.7, 1.1, 0.4 Hz, H_{6endo}), 3.87 (1H, dd, *J* 7.7, 5.8 Hz, H_{6exo}), 3.80 (1H, br. dq, *J* 11.3, 1.5 Hz, H_4), 2.60 (1H, d, *J* 11.3 Hz, OH_4) ppm. **^{13}C NMR** (101 MHz, $CDCl_3$) δ 98.56 (1C, d, *J* 27.1 Hz, C_1), 88.18 (1C, dd, *J* 181.2, 30.1 Hz, C_3), 84.35 (1C, dd, *J* 180.1, 28.2 Hz, C_2), 75.71 (1C, s, C_5), 67.67 (1C, dd, *J* 27.5, 1.8 Hz, C_4), 64.76 (1C, d, *J* 4.4 Hz, C_6) ppm. **^{19}F NMR** (471 MHz, $CDCl_3$) δ -187.42 (1F, m, *J* 43.2, 14.3, 12.3 Hz, F_3), -193.72 (1F, m, *J* 44.1, 15.0, 12.3 Hz, F_2) ppm. **^{19}F[^{1}H]** NMR (471 MHz, $CDCl_3$) δ -187.42 (1F, d, *J* 14.3 Hz, F_3), -193.72 (1F, d, *J* 15.0 Hz, F_2) ppm. **MS** (CI) (m/z) 167.0 $[M+H]^+$(21%). ^{1}H and ^{13}C NMR had not yet been described on $CDCl_3$.

1.6 Characterisation data of 1,6-anhydro-2,4-dideoxy-2,4-difluoro-β-D-glucopyranoside (**9**)

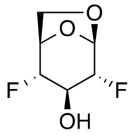

9

Mp 96° (chloroform/acetone), lit 99-100°C (no solvent given).[1] **[α]$_D$** -63.0 (c 0.82, water, 21 °C), lit -62 (c 0.82, water 20 °C).[1] **^{1}H NMR** (500 MHz, $CDCl_3$) δ 5.61 (1H, br. dt, *J* 3.8, 1.4 Hz, H_1), 4.78 (1H, br. ddq, *J* 12.9, 5.7, 1.3 Hz, H_5), 4.45 (1H, m, *J* 46.4 Hz, H_4), 4.30 (1H, br. dquin, *J* 46.4, 1.3 Hz, H_2), 4.11 (1H, m, *J* 18.0, 6.1 Hz, H_3), 4.04 (1H, dt, *J* 7.8, 0.9 Hz, H_{6endo}), 3.80 (1H, ddddd app as m, *J* 7.8, 5.4, 4.3, 1.1, 0.4 Hz, H_{6exo}), 2.30 (1H, br. d, *J* 6.1 Hz, OH_3) ppm. **^{13}C NMR** (101 MHz, $CDCl_3$) δ 99.33 (1C, d, *J* 28.6 Hz, C_1), 89.88 (1C, dd, *J* 181.9, 5.1 Hz, C_4), 88.02 (1C, dd, *J* 184.1, 4.4 Hz, C_2), 74.33 (1C, d, *J* 22.7 Hz, C_5), 69.46 (1C, dd, *J* 29.3, 27.9 Hz, C_3), 64.74 (1C, d, *J* 9.5 Hz, C_6) ppm. **^{19}F NMR** (471 MHz, $CDCl_3$) δ -183.32 (1F, ddddd app as m, *J* 46.3, 17.3, 12.8, 4.1, 0.9 Hz, F_4), -188.39 (1F, ddd, *J* 46.5, 18.4, 3.9 Hz, F_2) ppm. **^{19}F[^{1}H] NMR** (471 MHz, $CDCl_3$) δ -183.32 (1F, s, F_4), -188.39 (1F, s, F_2) ppm. ^{1}H and ^{13}C NMR had not yet been described in $CDCl_3$.

2 Spectral details for OH coupling constants

2.1 Sample preparation

Samples were prepared as follows : dried molecular sieves (3Å) were added (0.3 v/v) to the solution of the levoglucosan derivative in $CDCl_3$ in a 4 mL vial. The solution was stirred overnight at room temperature. The sieves were allowed to settle and the supernatant was transferred to a dried (oven + vacuum) NMR tube. The vial was kept under argon, and the transfer was carried out with a dried syringe flushed with argon.

2.2 Compound **4**

2.2.1 Spectrum in $CDCl_3$ with <1% H_2O

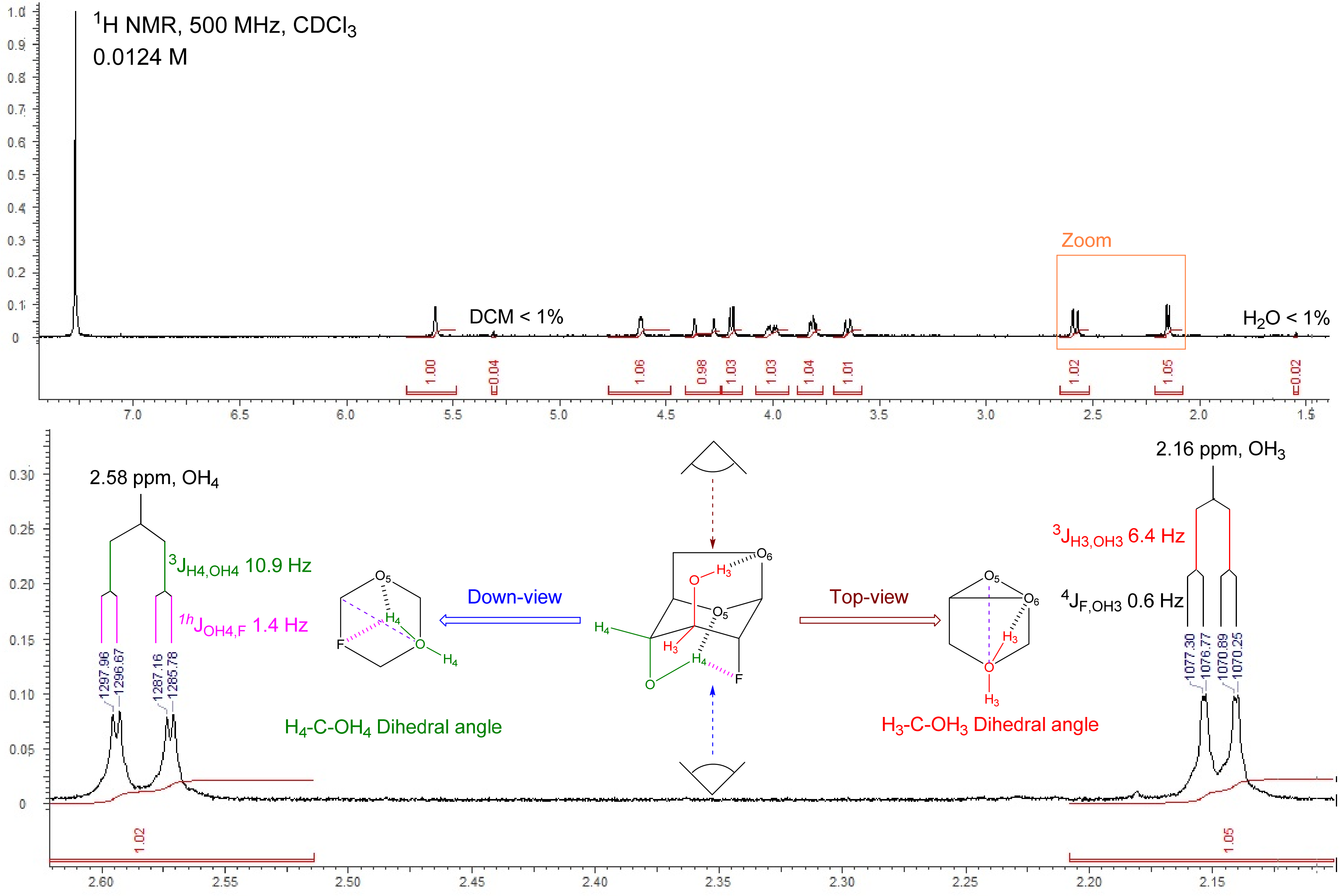

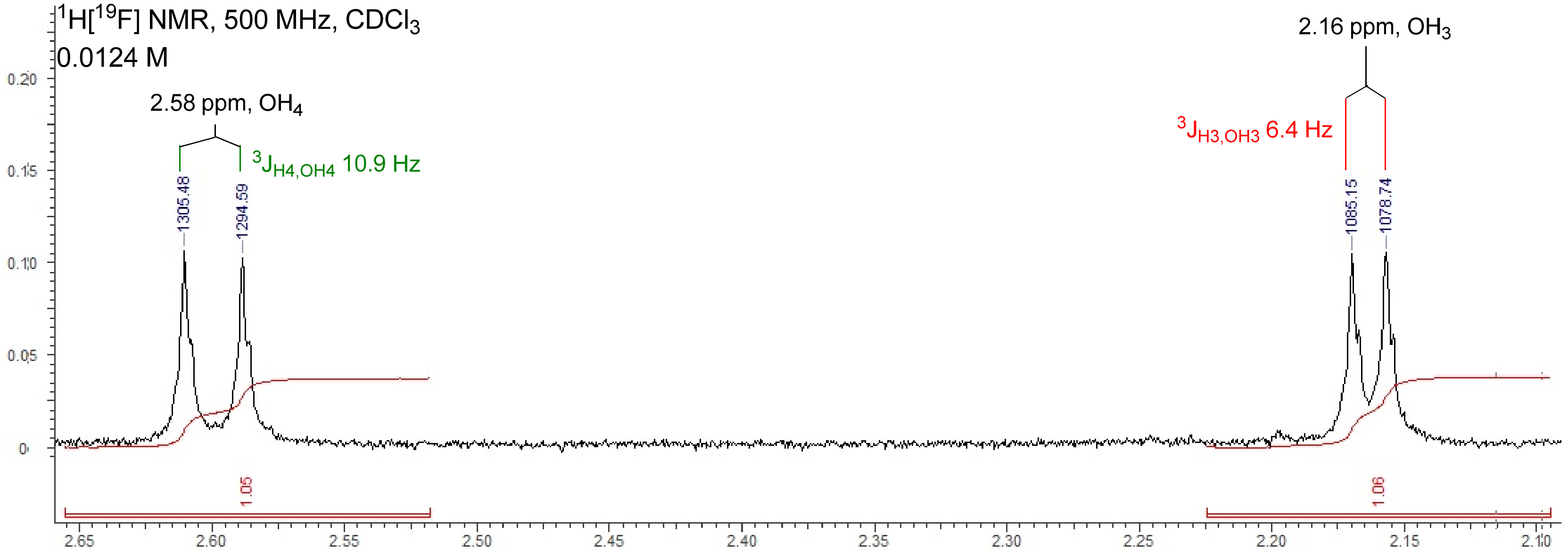

1H[19F] NMR, 500 MHz, CDCl3
0.0124 M
2.58 ppm, OH4
3JH4,OH4 10.9 Hz
2.16 ppm, OH3
3JH3,OH3 6.4 Hz
1305.48
1294.59
1085.15
1078.74
1.05
1.06
0.20
0.15
0.10
0.05
0
2.65
2.60
2.55
2.50
2.45
2.40
2.35
2.30
2.25
2.20
2.15
2.10

2.2.2 Spectrum in $CDCl_3$ with 1 equiv of H_2O

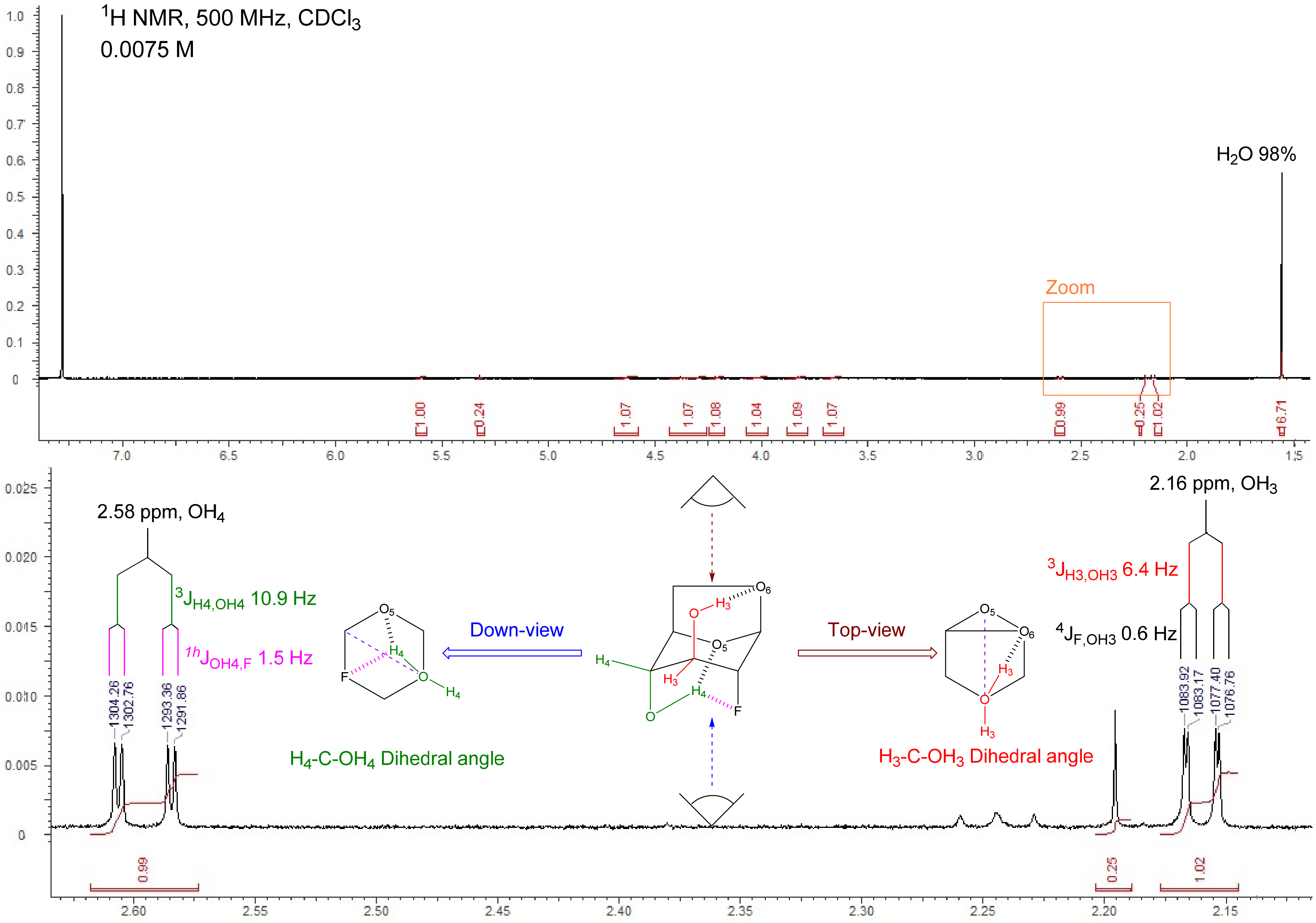

2.3 Compound **5**

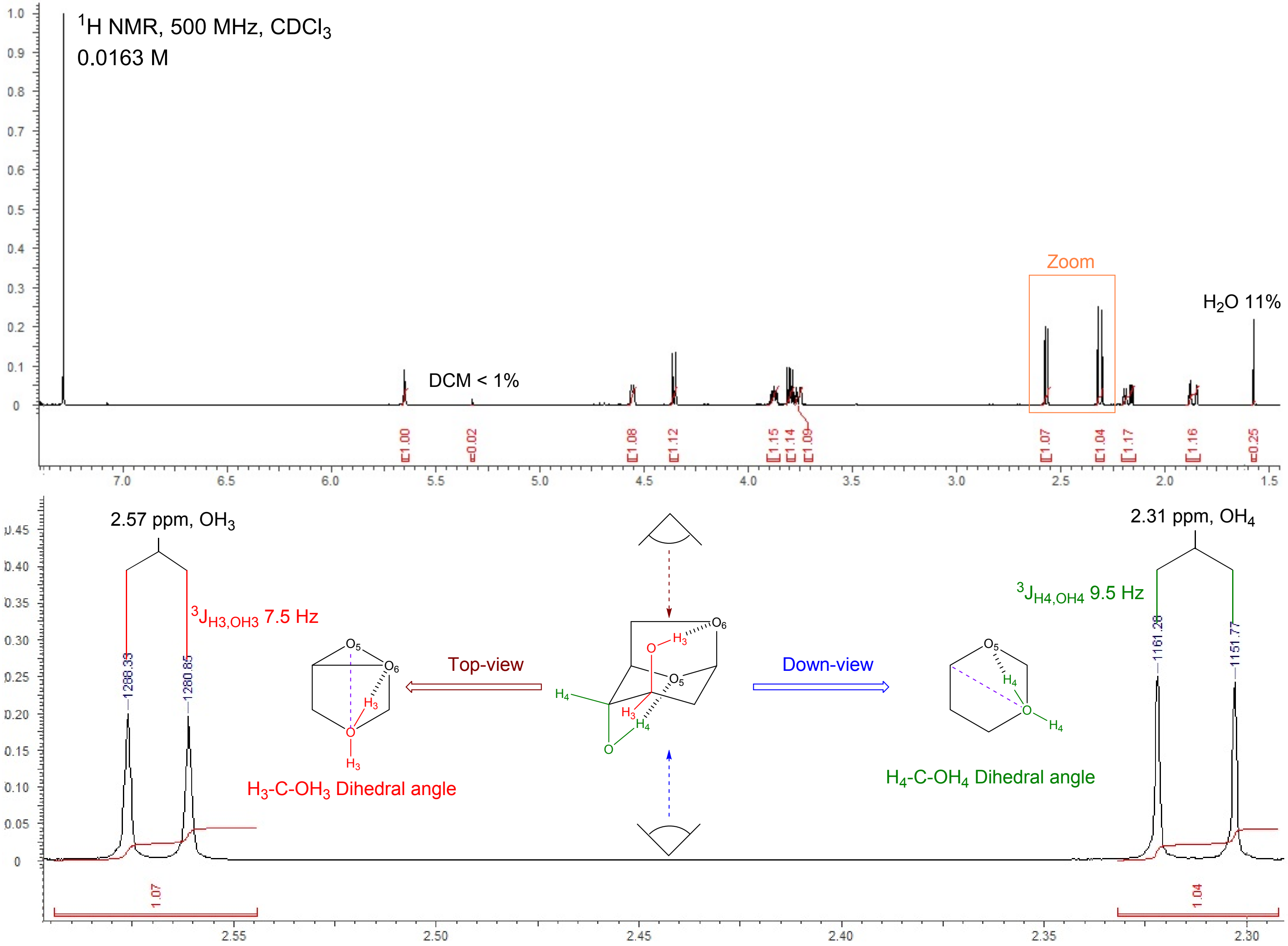

2.4 Compound **6**

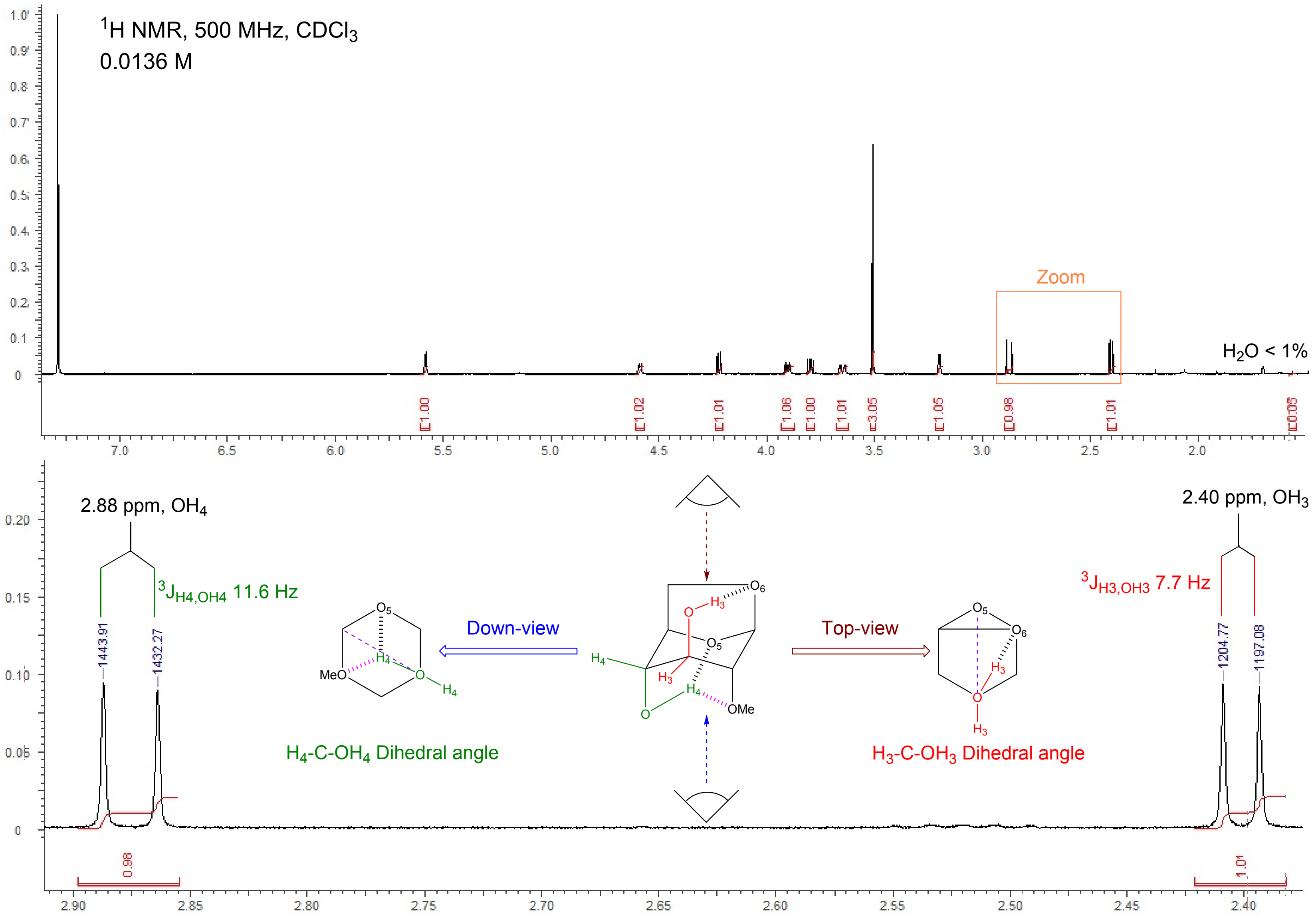

2.5 Compound **7**

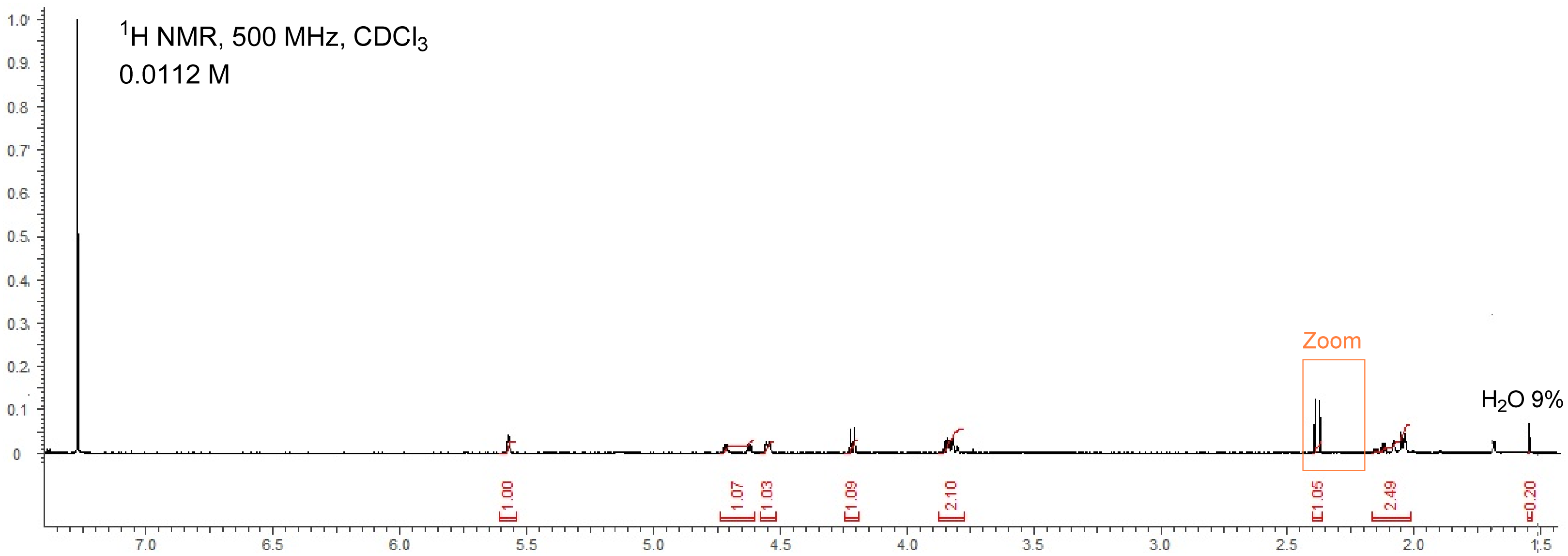

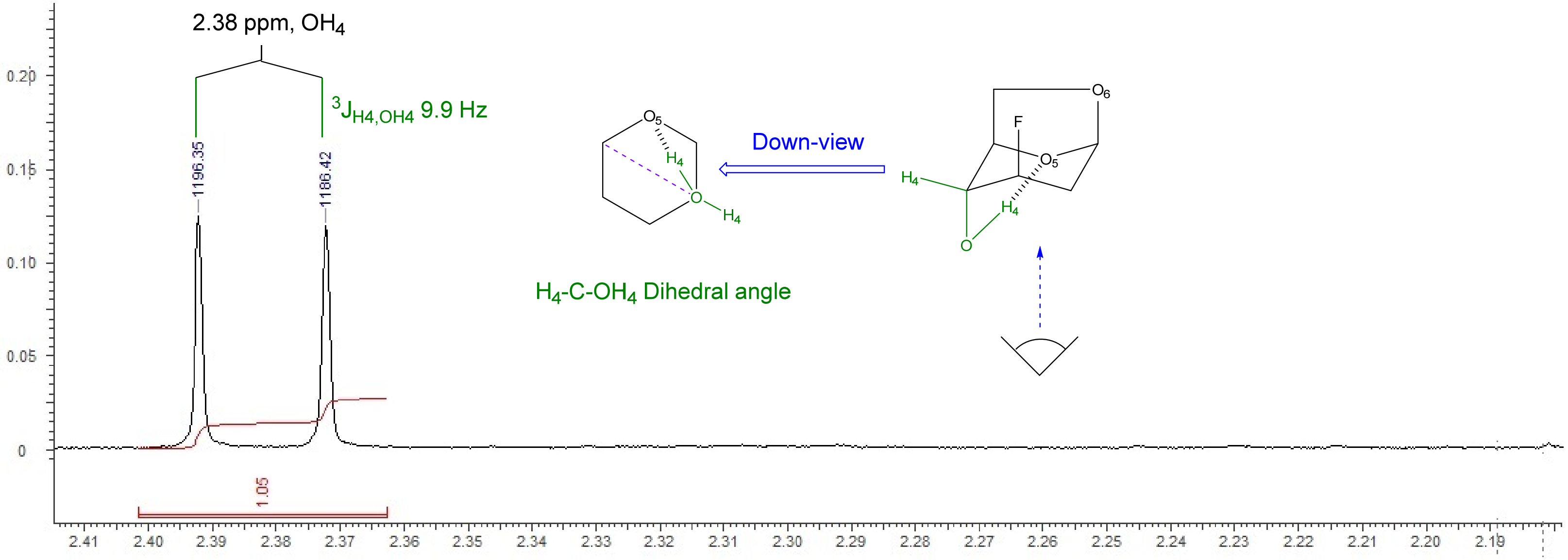

2.6 Compound **8**

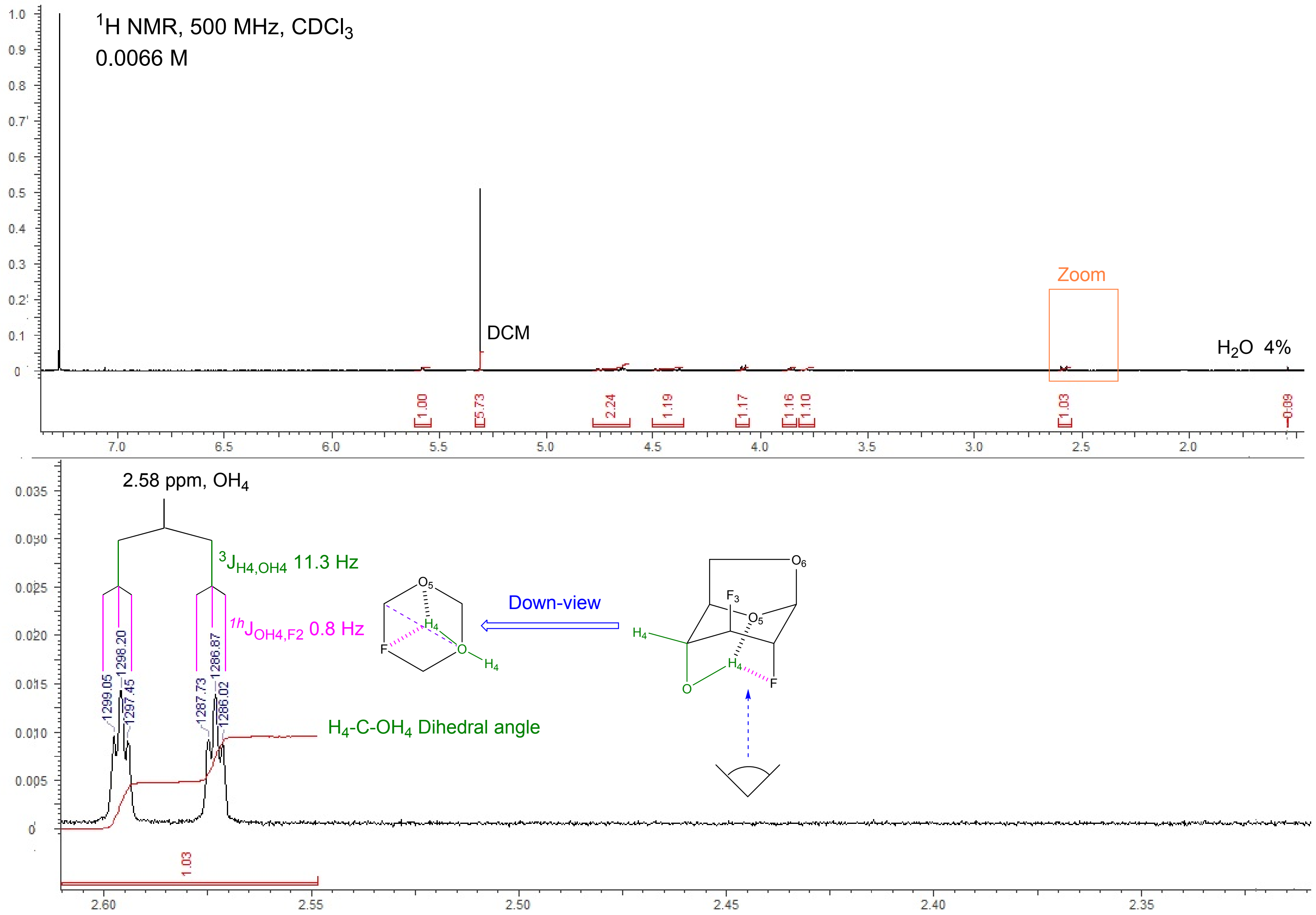

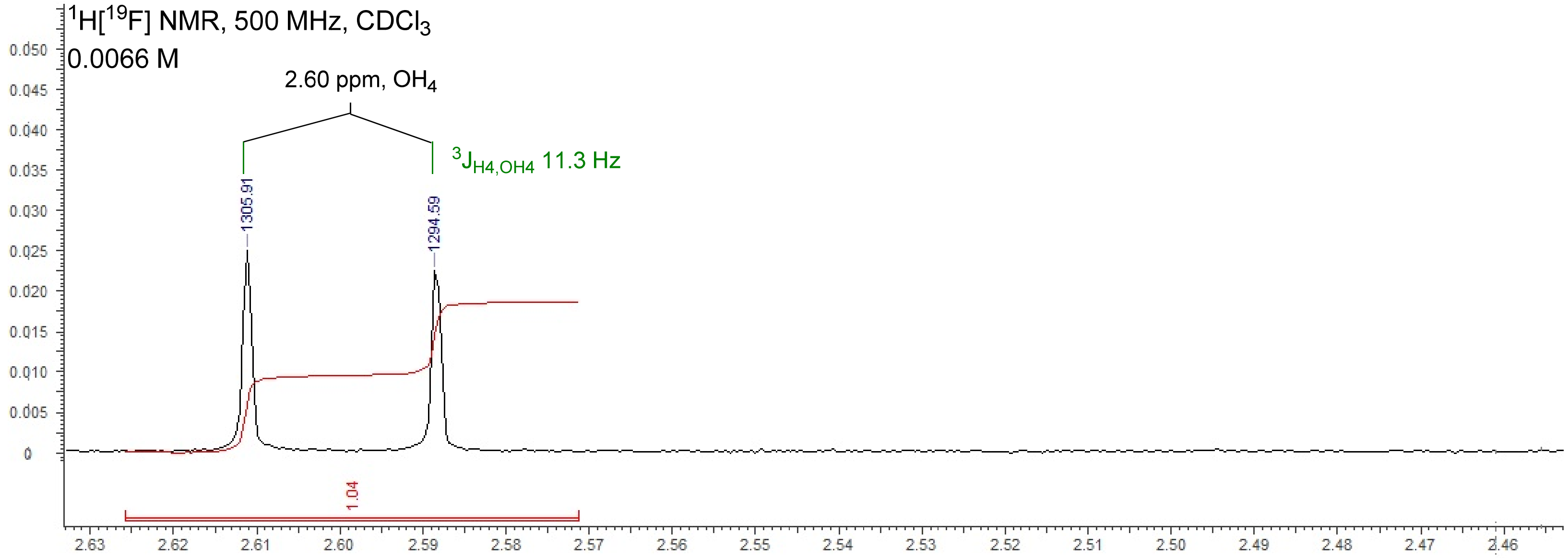

$^1H[^{19}F]$ NMR, 500 MHz, $CDCl_3$
0.0066 M
2.60 ppm, OH_4
$^3J_{H4,OH4}$ 11.3 Hz
1305.91
1294.59
1.04

2.7 Compound **9**

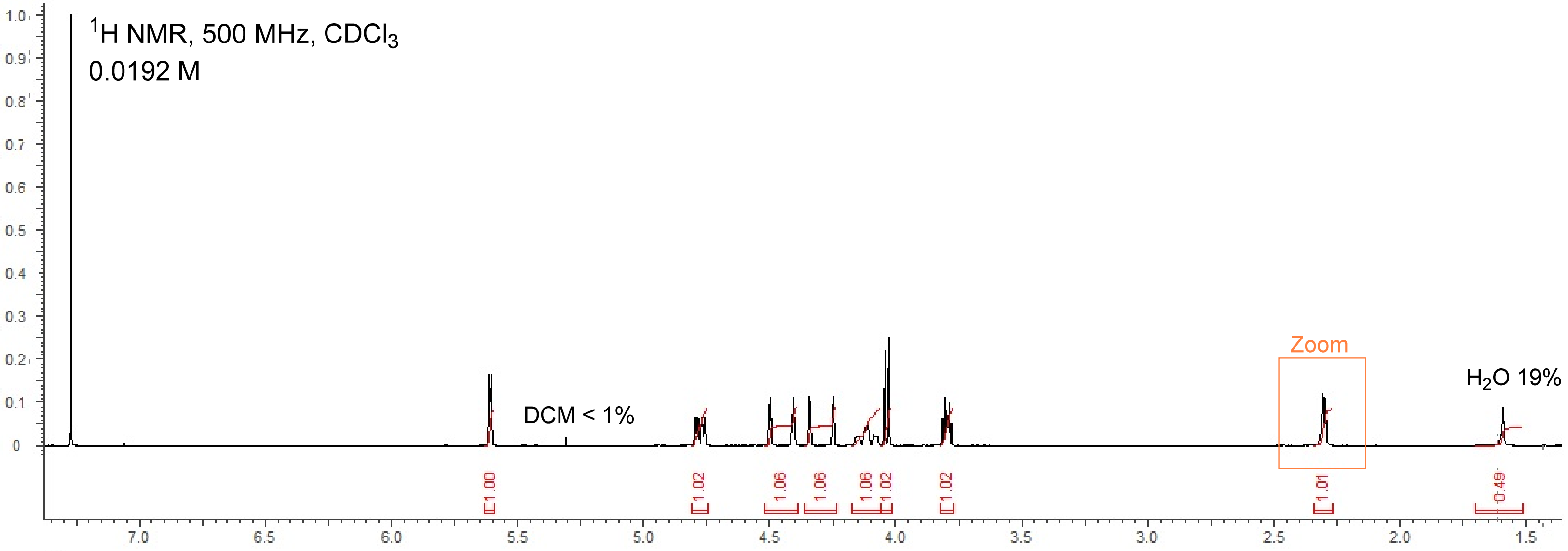

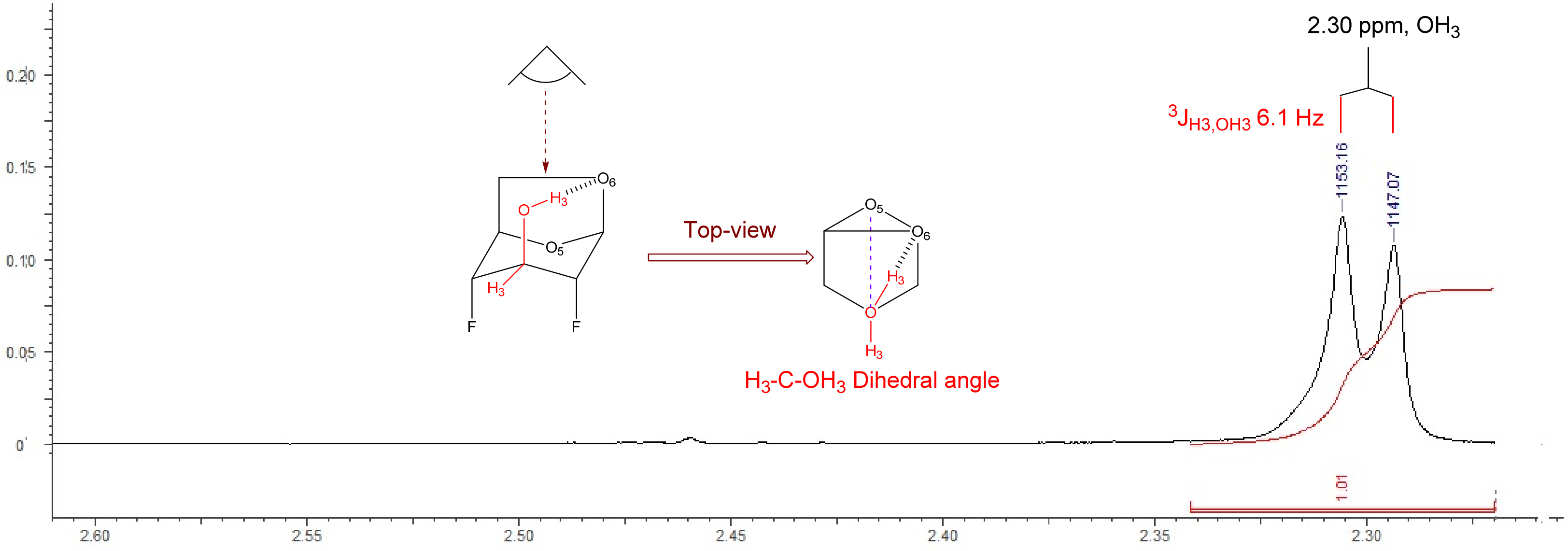

3 Computational data

3.1 Compound **4**

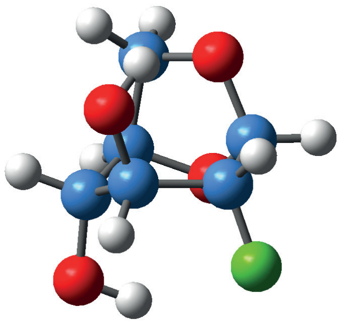

4 *t_t*, 49.8%

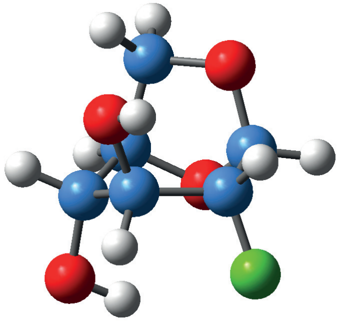

4 *t_g*, 23.7%

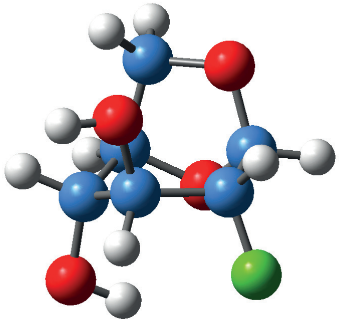

4 *t_g-*, 20.2%

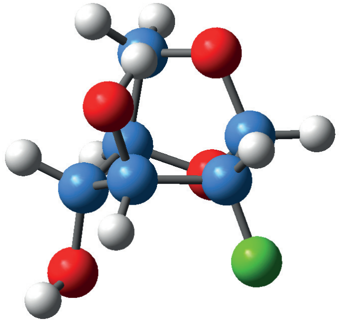

4 *g-_t*, 1.7%

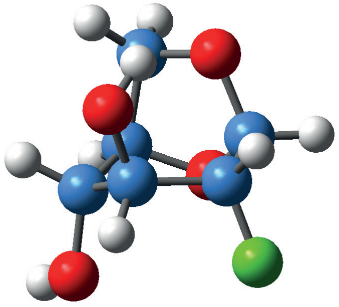

4 *g_t*, 1.4%

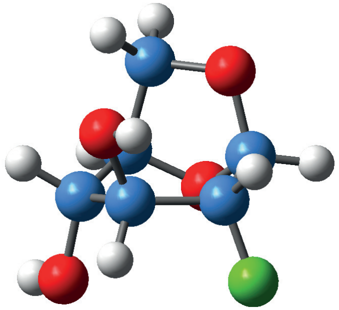

4 *g_g*, 1.1%

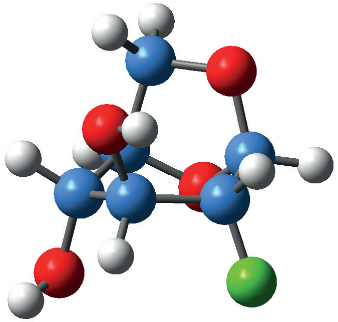

4 *g-_g*, 0.9%

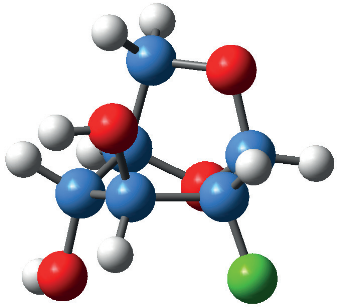

4 *g_g-*, 0.8%

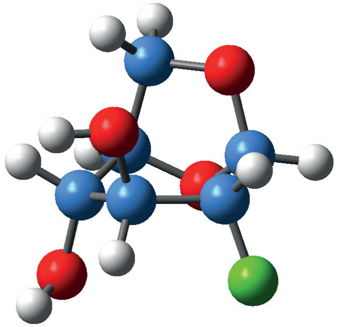

4 *g-_g-*, 0.5%

3.2 Compound **5**

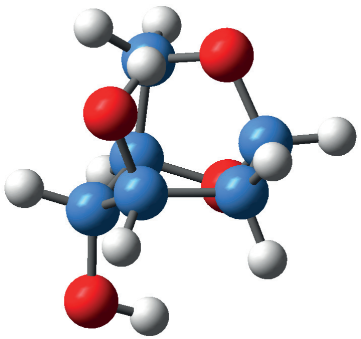

5 *t_t*, 65.2%

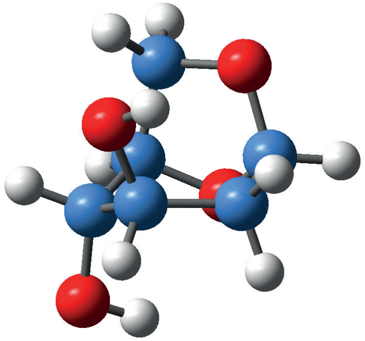

5 *t_g*, 14.0%

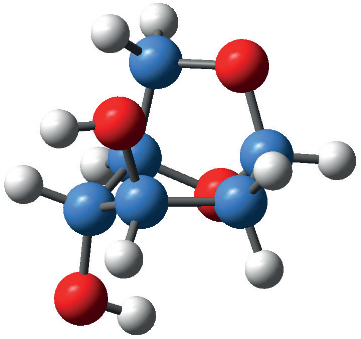

5 *t_g-*, 7.5%

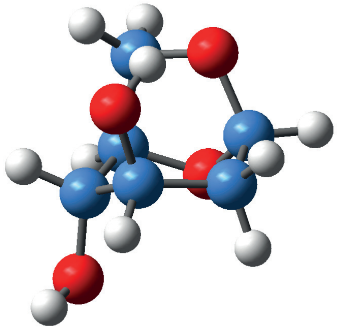

5 *g-_t*, 7.0%

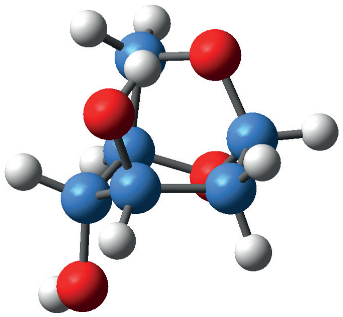

5 *g_t*, 5.5%

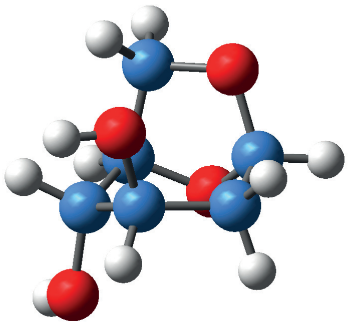

5 *g_g-*, 0.5%

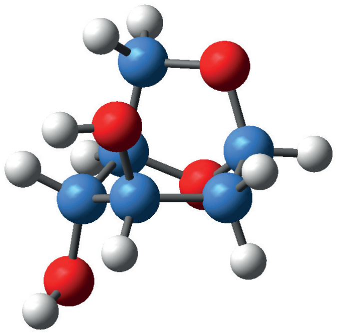

5 *g-_g-*, 0.3%

3.3 Compound **6**

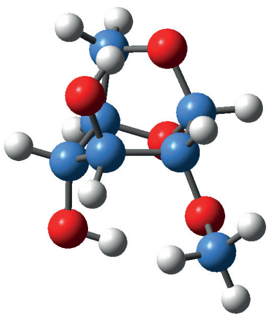

6 *t_t_g*, 48.1%

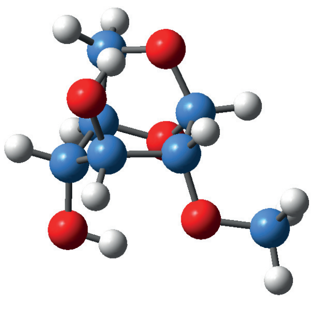

6 *t_t_g-*, 23.7%

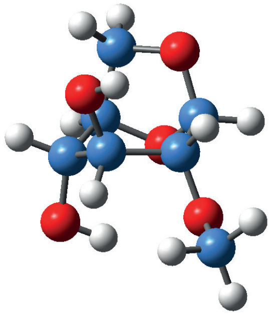

6 *t_g_g*, 9.2%

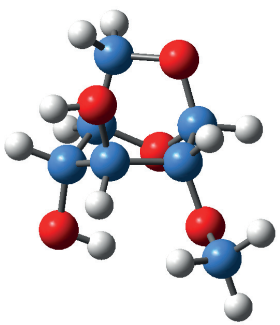

6 *t_g-_g*, 7.8%

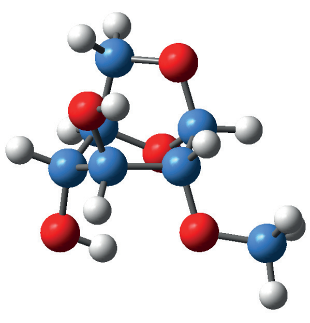

6 *t_g_g-*, 5.2%

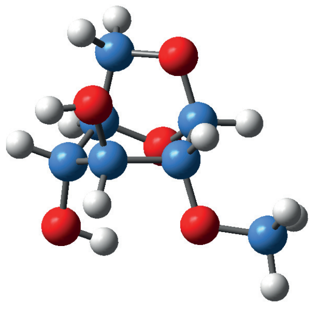

6 *t_g-_g-*, 4.8%

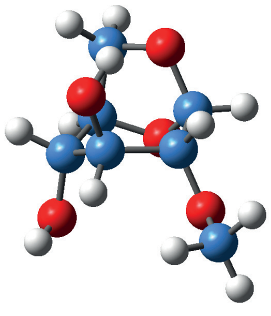

6 *g-_t_g*, 0.5%

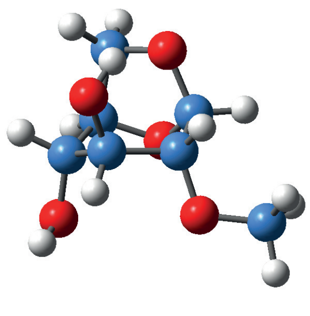

6 *g-_t_g-*, 0.3%

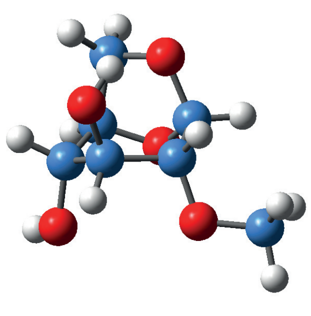

6 *g_t_g-*, 0.3%

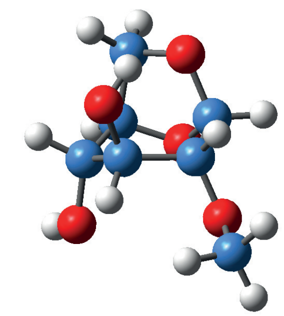

6 *g_t_g*, 0.2%

3.4 Compound **7**

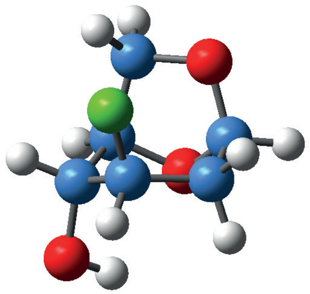

7 *t*, 82.9%

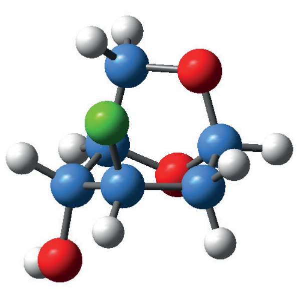

7 *g*, 10.0%

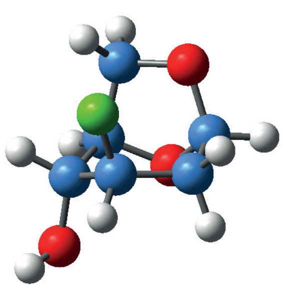

7 *g-*, 7.1%

3.5 Compound **8**

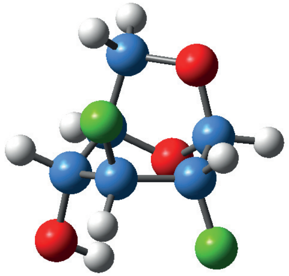

8 *t*, 92.6%

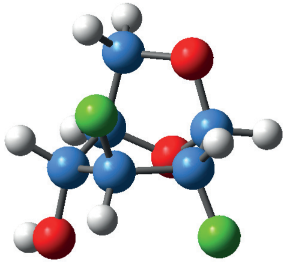

8 *g*, 4.3%

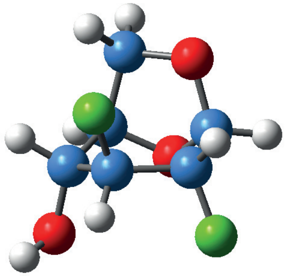

8 *g*-, 3.1%

3.6 Compound **9**

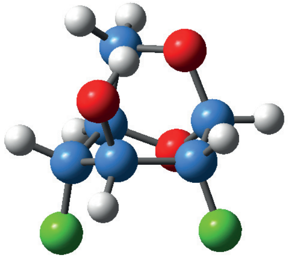

9 *t*, 51.0%

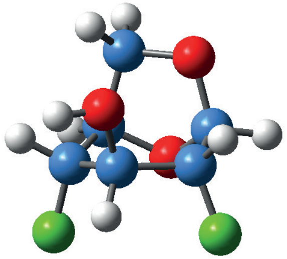

9 *g*-, 24.9%

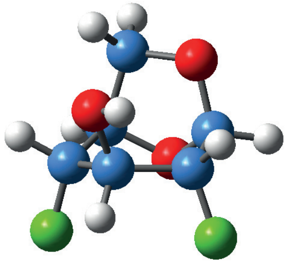

9 *g*, 24.1%

3.7 Superposition of the *t_t* conformers of **4**, **5** and **6** demonstrating the non influence of the C2 substituent on the levoglucosan scaffold.

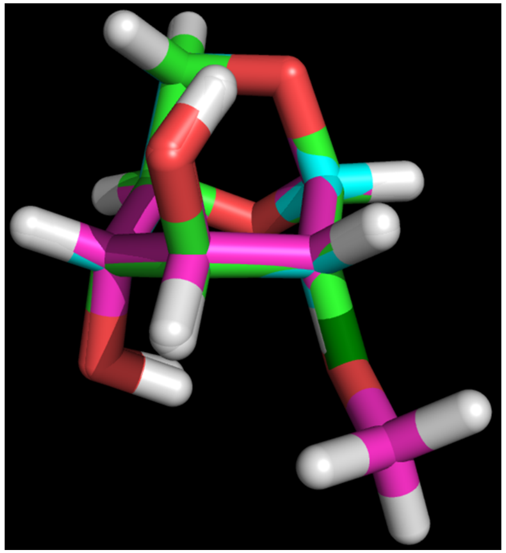

4 ^{1}H, ^{19}F and ^{13}C spectra of all compounds

4.1 Compound 4

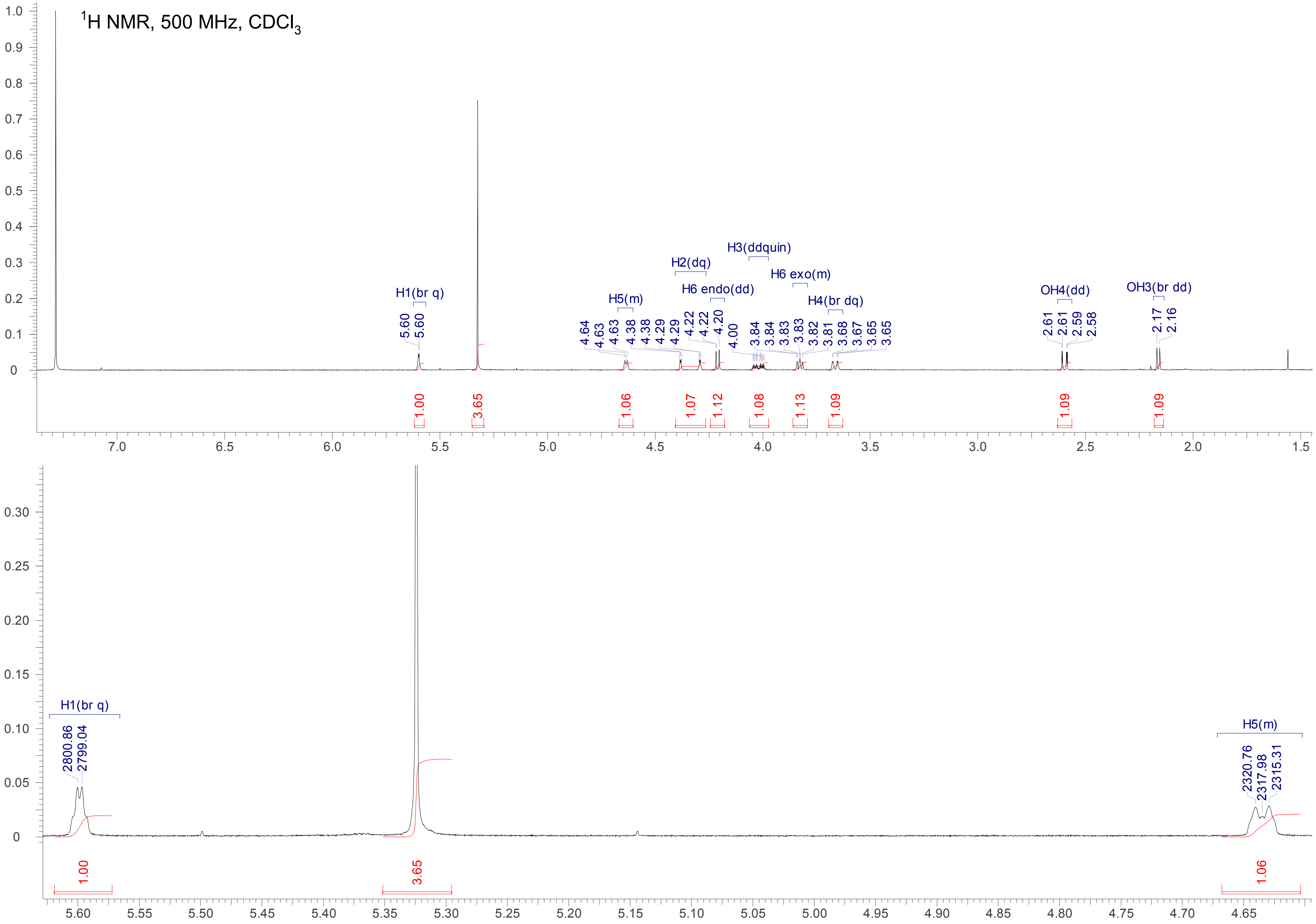

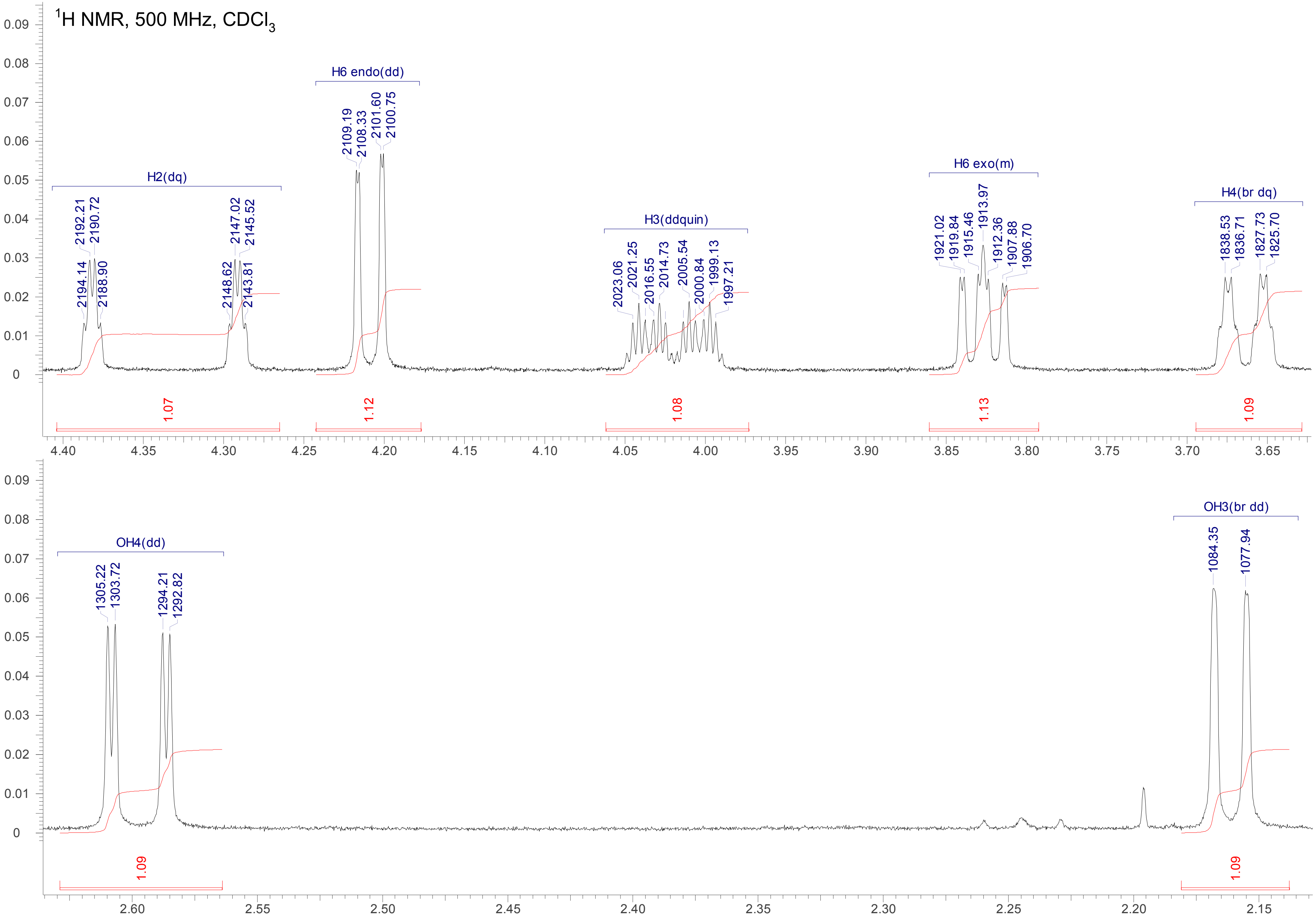

1H NMR, 500 MHz, CDCl3
H2(dq)
H6 endo(dd)
H3(ddquin)
H6 exo(m)
H4(br dq)
OH4(dd)
OH3(br dd)

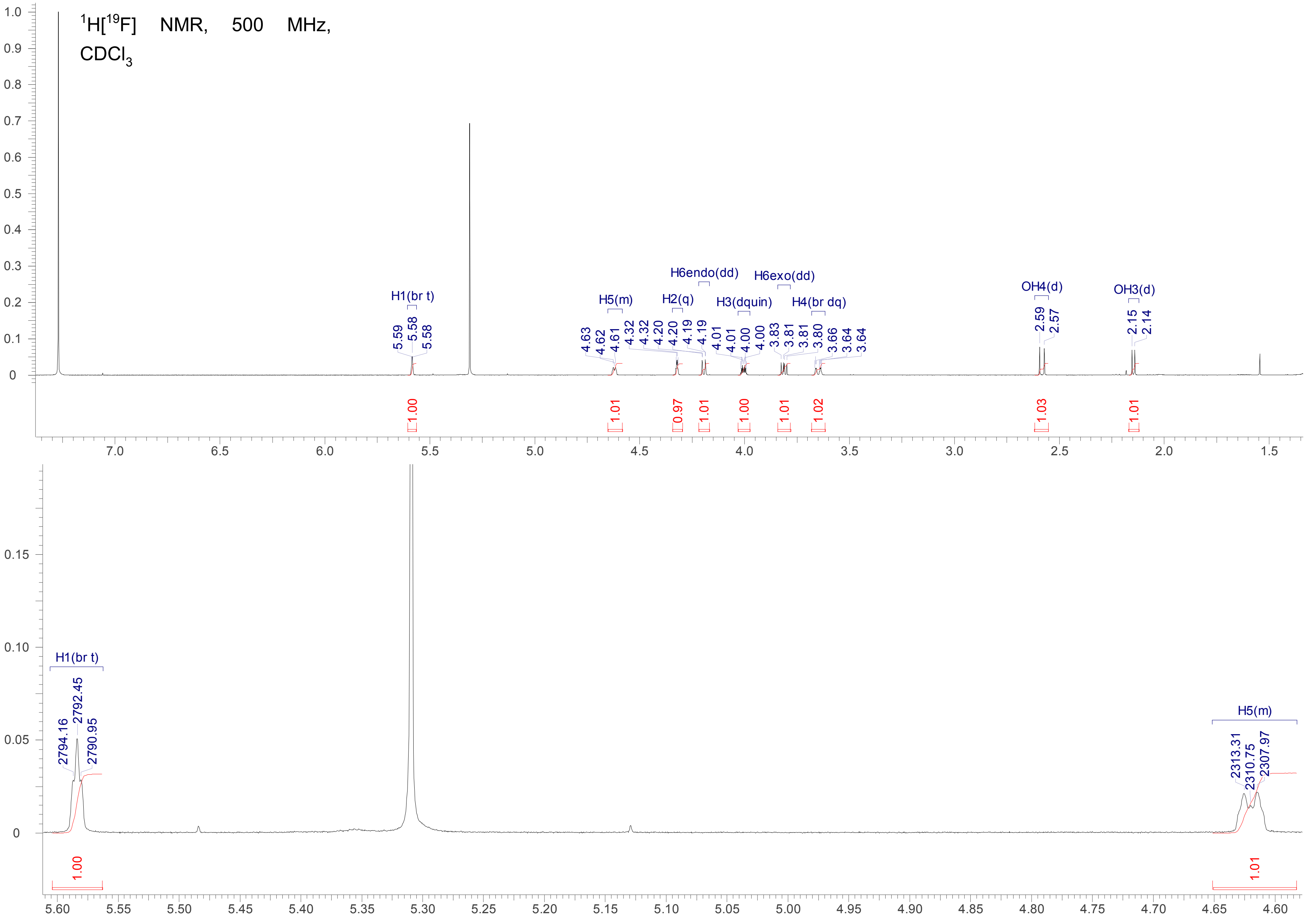

$^1H[^{19}F]$ NMR, 500 MHz, CDCl$_3$
H1(br t)
H5(m)
H2(q)
H6endo(dd)
H3(dquin)
H6exo(dd)
H4(br dq)
OH4(d)
OH3(d)

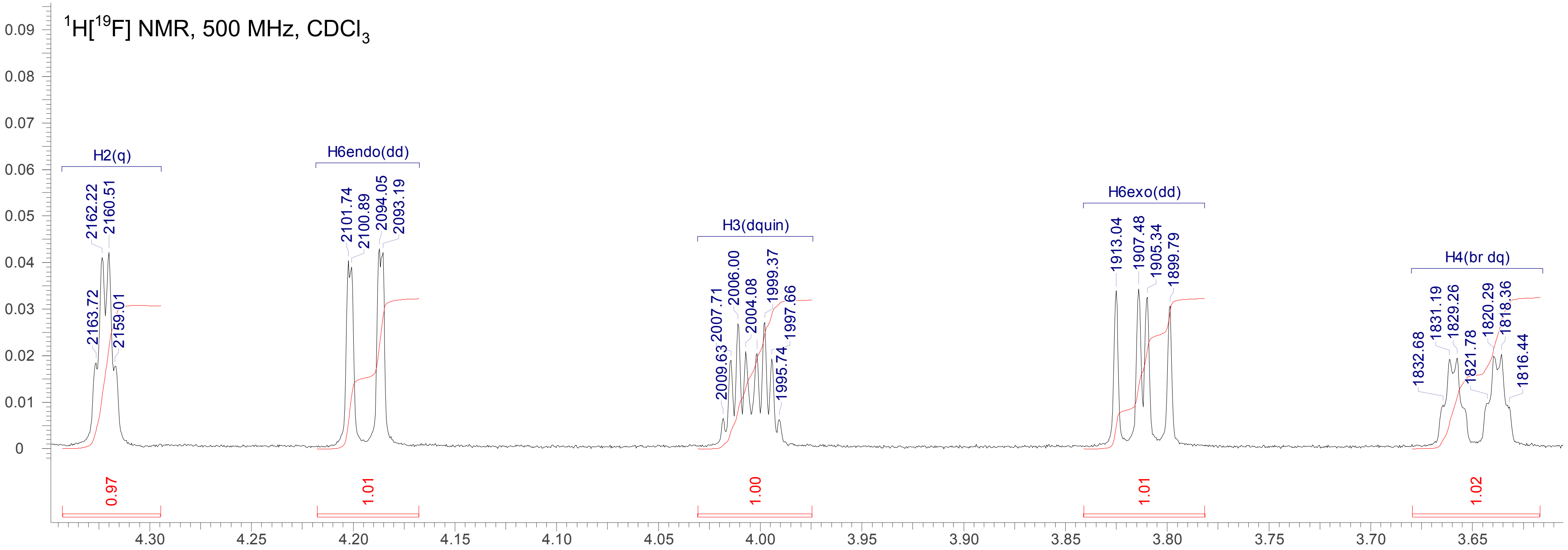
$^1H[^{19}F]$ NMR, 500 MHz, $CDCl_3$
H2(q)
2162.22
2160.51
2163.72
2159.01
H6endo(dd)
2101.74
2100.89
2094.05
2093.19
H3(dquin)
2009.63
2007.71
2006.00
2004.08
1999.37
1997.66
1995.74
H6exo(dd)
1913.04
1907.48
1905.34
1899.79
H4(br dq)
1832.68
1831.19
1829.26
1821.78
1820.29
1818.36
1816.44
0.97
1.01
1.00
1.01
1.02

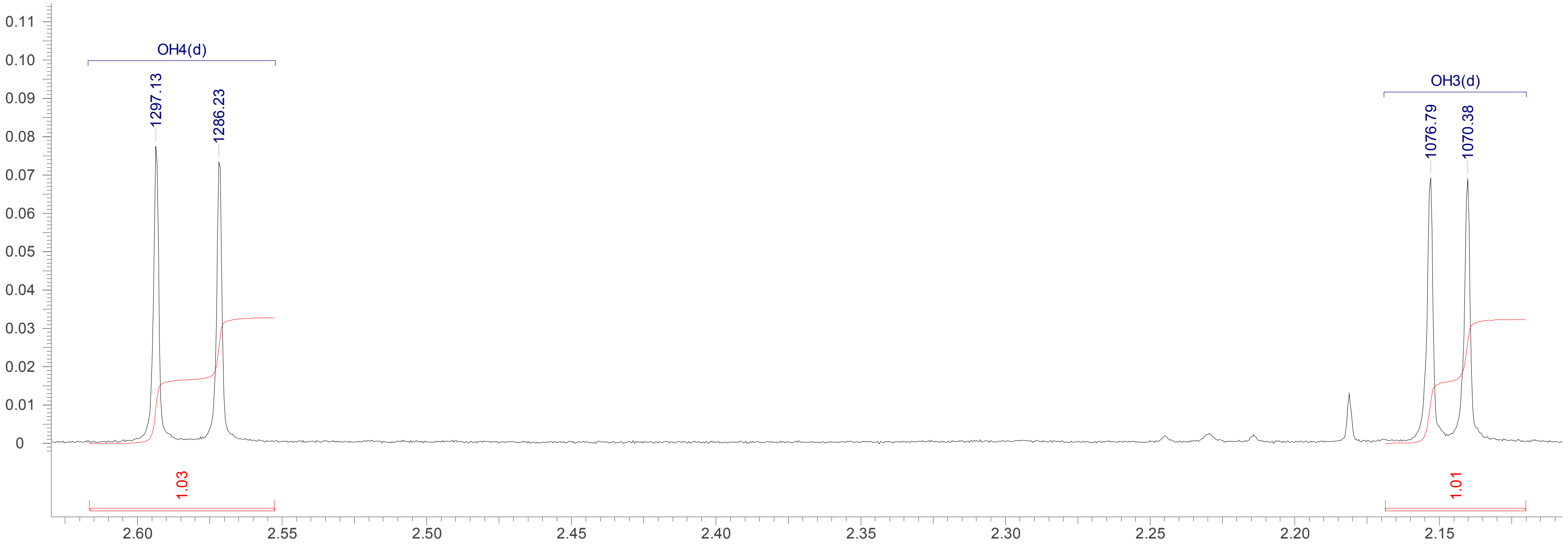
OH4(d)
1297.13
1286.23
OH3(d)
1076.79
1070.38
1.03
1.01

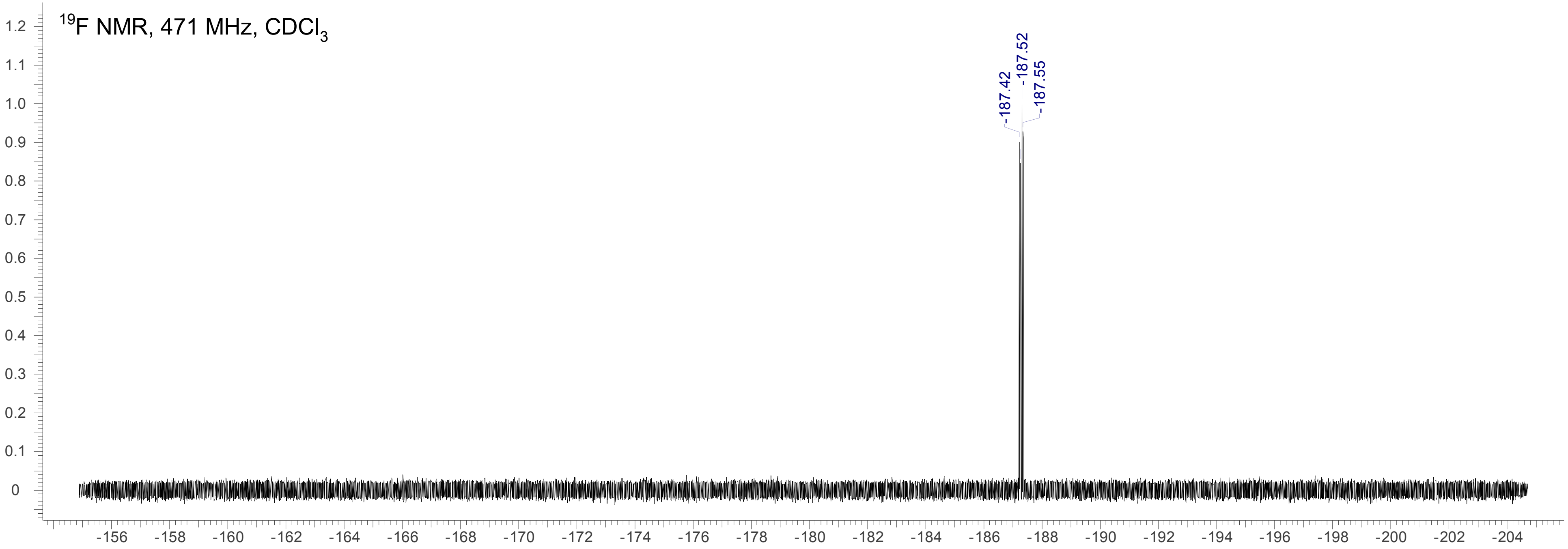
^{19}F NMR, 471 MHz, CDCl$_3$
-187.42
-187.52
-187.55

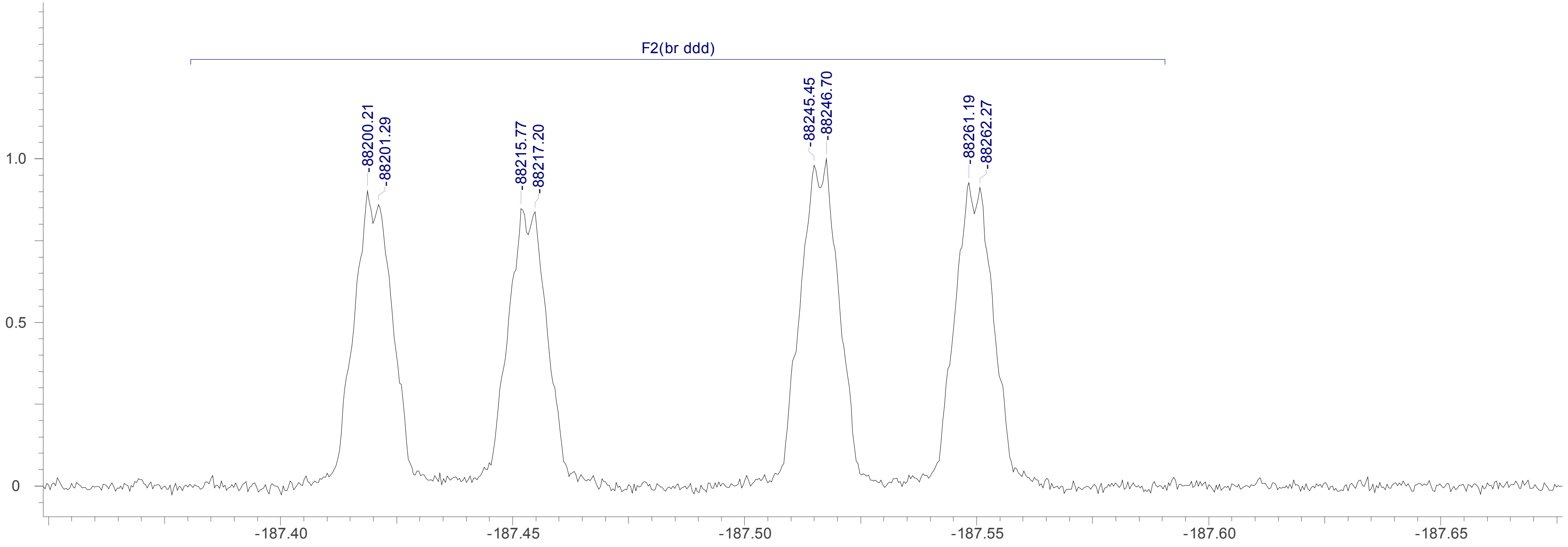
F2(br ddd)
-88200.21
-88201.29
-88215.77
-88217.20
-88245.45
-88246.70
-88261.19
-88262.27

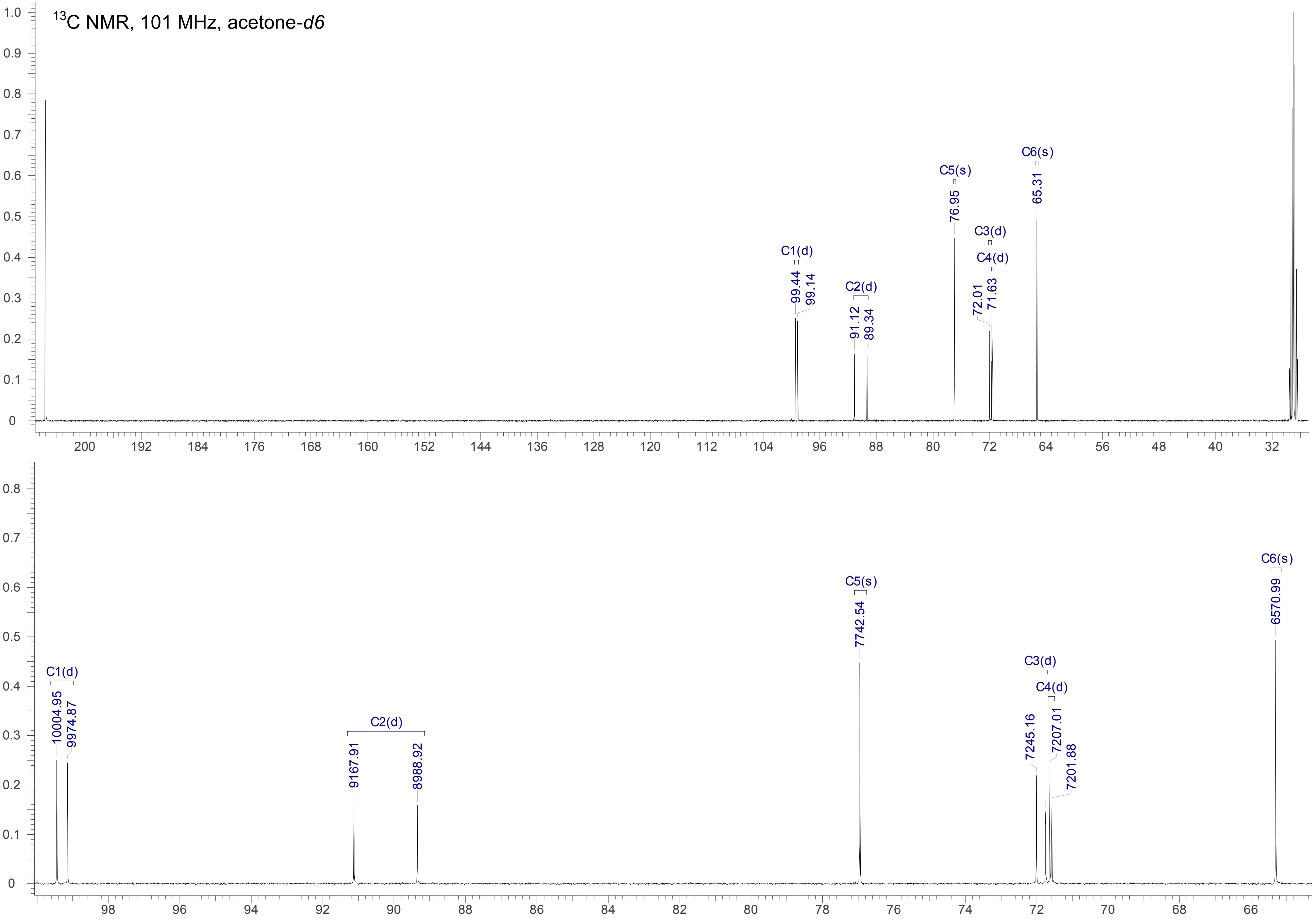

13C NMR, 101 MHz, acetone-d6
C1(d)
99.44
99.14
C2(d)
91.12
89.34
C5(s)
76.95
C3(d)
C4(d)
72.01
71.63
C6(s)
65.31
C1(d)
10004.95
9974.87
C2(d)
9167.91
8988.92
C5(s)
7742.54
C3(d)
C4(d)
7245.16
7207.01
7201.88
C6(s)
6570.99

4.2 Compound 5

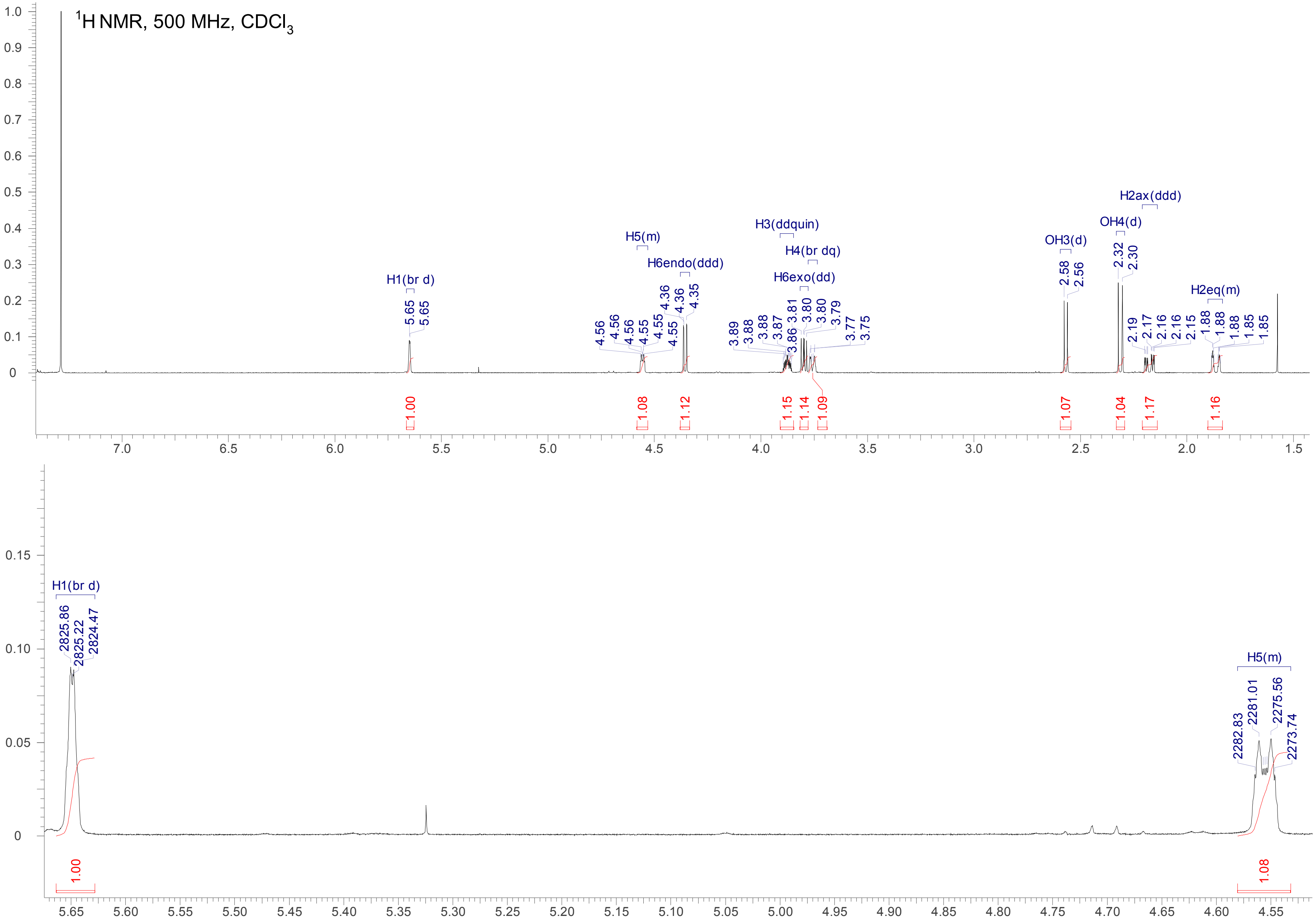

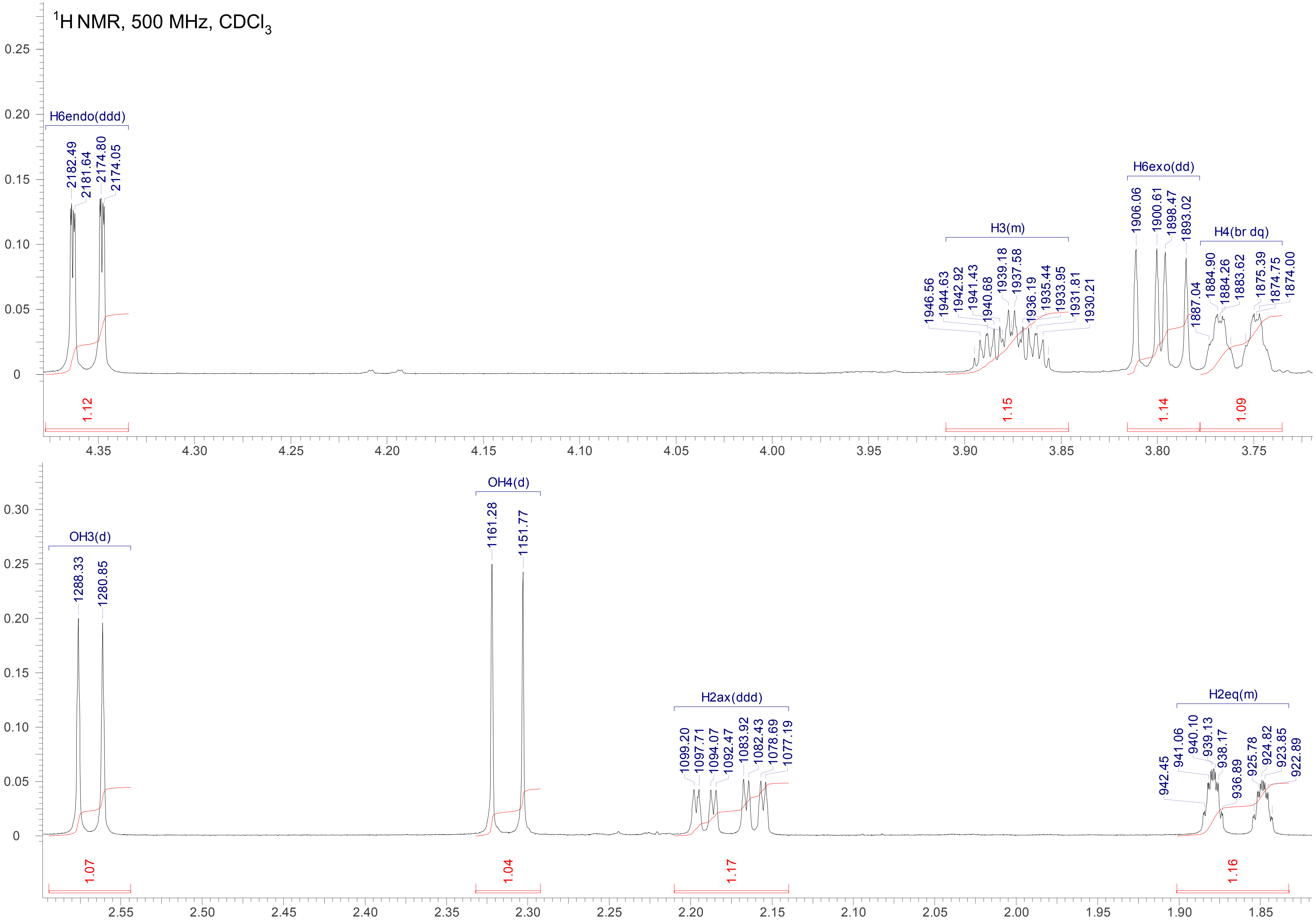

^{1}H NMR, 500 MHz, $CDCl_3$
H6endo(ddd)
2182.49
2181.64
2174.80
2174.05
H3(m)
1946.56
1944.63
1942.92
1941.43
1940.68
1939.18
1937.58
1936.19
1935.44
1933.95
1931.81
1930.21
H6exo(dd)
1906.06
1900.61
1898.47
1893.02
H4(br dq)
1887.04
1884.90
1884.26
1883.62
1875.39
1874.75
1874.00
1.12
1.15
1.14
1.09
OH3(d)
1288.33
1280.85
OH4(d)
1161.28
1151.77
H2ax(ddd)
1099.20
1097.71
1094.07
1092.47
1083.92
1082.43
1078.69
1077.19
H2eq(m)
942.45
941.06
940.10
939.13
938.17
936.89
925.78
924.82
923.85
922.89
1.07
1.04
1.17
1.16

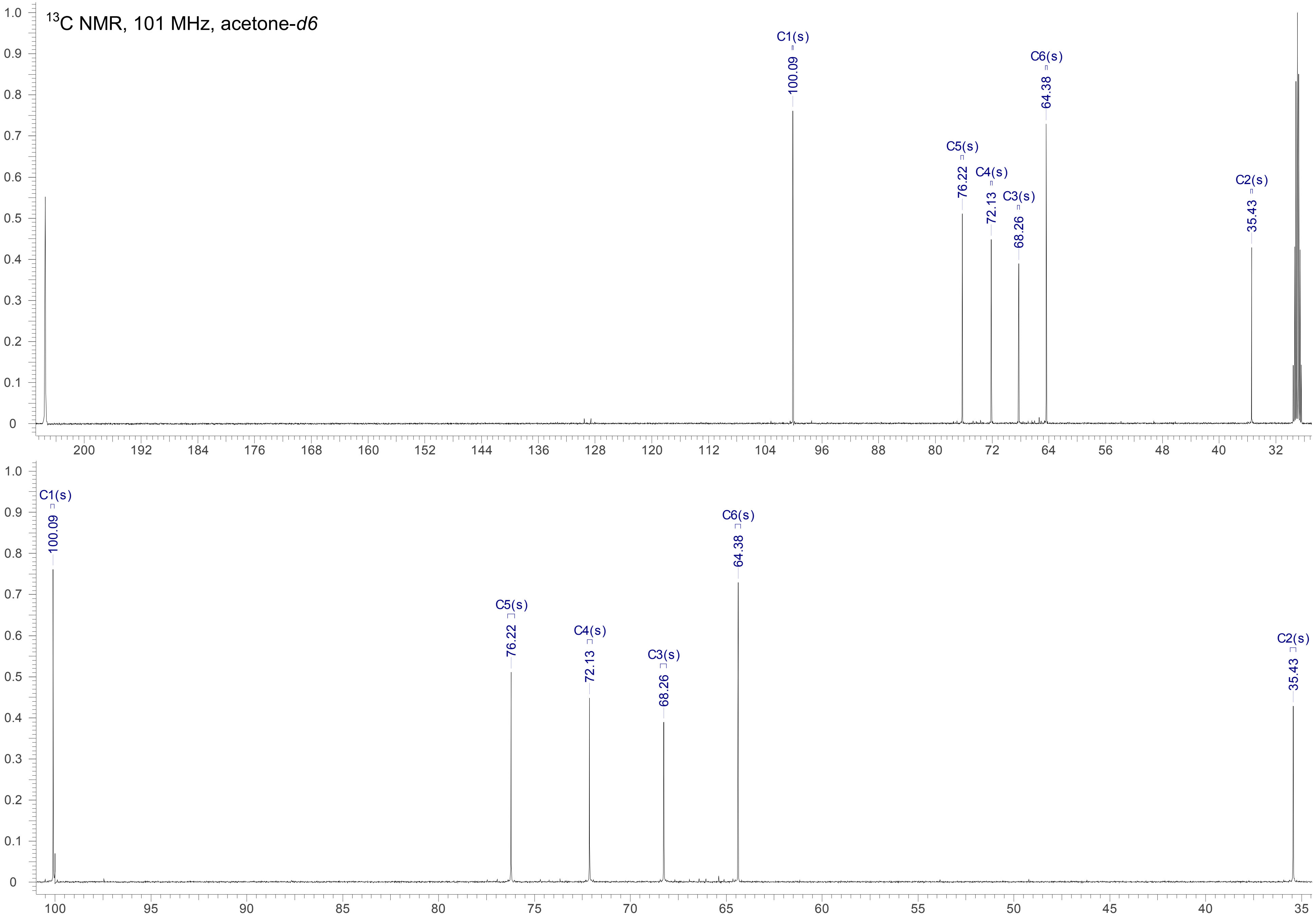

13C NMR, 101 MHz, acetone-d6
C1(s)
100.09
C5(s)
76.22
C4(s)
72.13
C3(s)
68.26
C6(s)
64.38
C2(s)
35.43

4.3 Compound 6

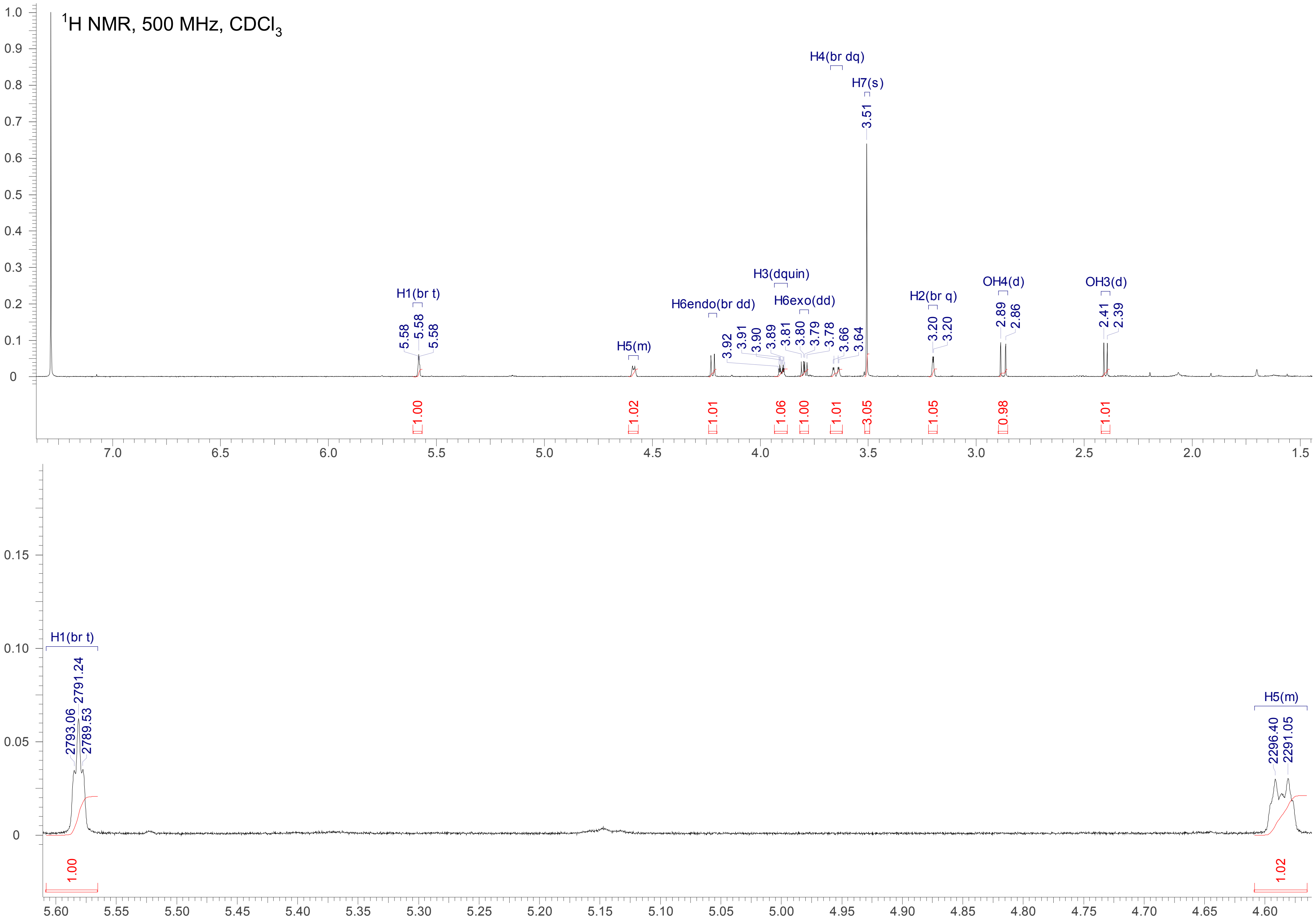

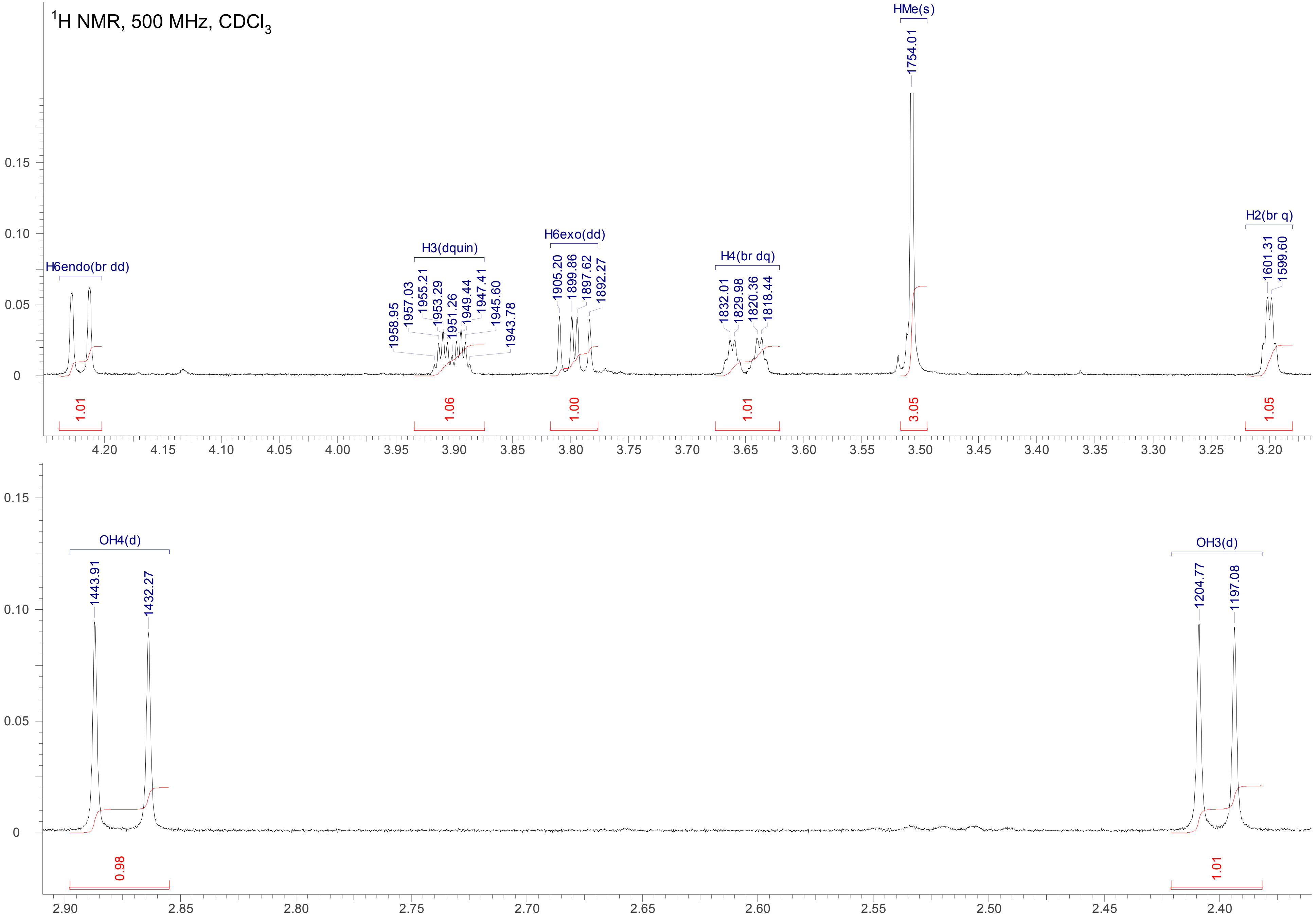

^{1}H NMR, 500 MHz, $CDCl_3$
H6endo(br dd)
H3(dquin)
1958.95
1957.03
1955.21
1953.29
1951.26
1949.44
1947.41
1945.60
1943.78
H6exo(dd)
1905.20
1899.86
1897.62
1892.27
H4(br dq)
1832.01
1829.98
1820.36
1818.44
HMe(s)
1754.01
H2(br q)
1601.31
1599.60
1.01
1.06
1.00
1.01
3.05
1.05
0.15
0.10
0.05
0
4.20 4.15 4.10 4.05 4.00 3.95 3.90 3.85 3.80 3.75 3.70 3.65 3.60 3.55 3.50 3.45 3.40 3.35 3.30 3.25 3.20
OH4(d)
1443.91
1432.27
OH3(d)
1204.77
1197.08
0.98
1.01
2.90 2.85 2.80 2.75 2.70 2.65 2.60 2.55 2.50 2.45 2.40

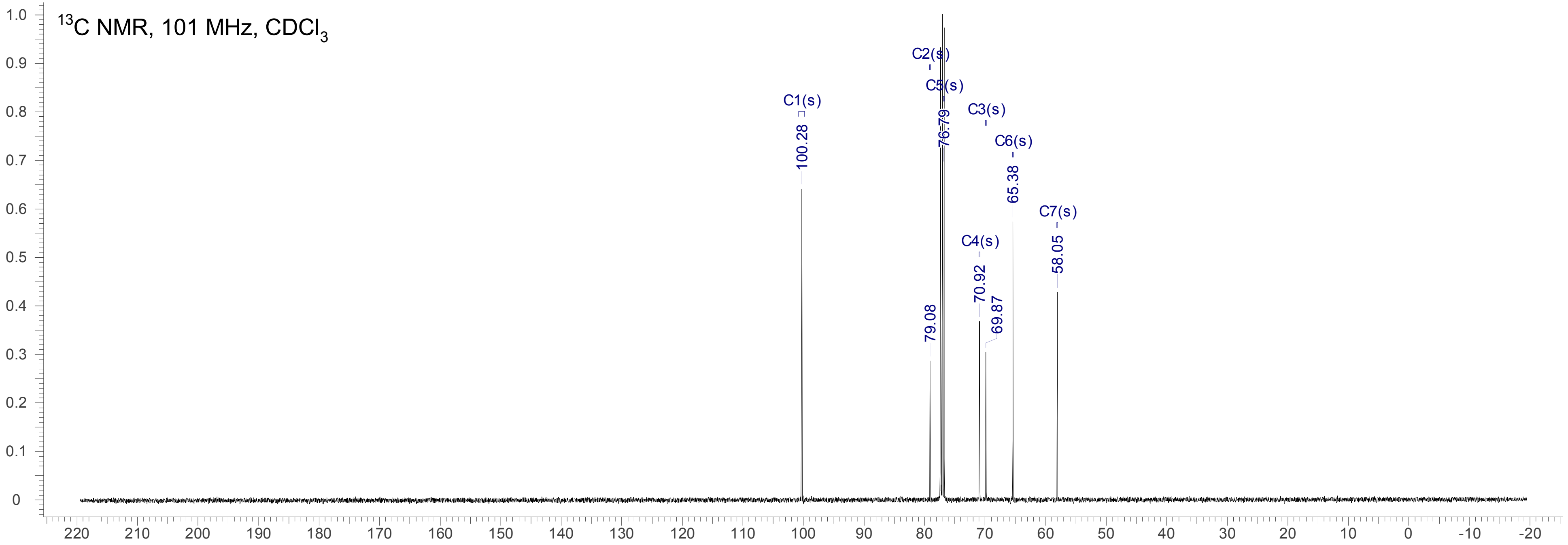
^{13}C NMR, 101 MHz, $CDCl_3$
C1(s)
100.28
C2(s)
79.08
C5(s)
76.79
C3(s)
69.87
C4(s)
70.92
C6(s)
65.38
C7(s)
58.05

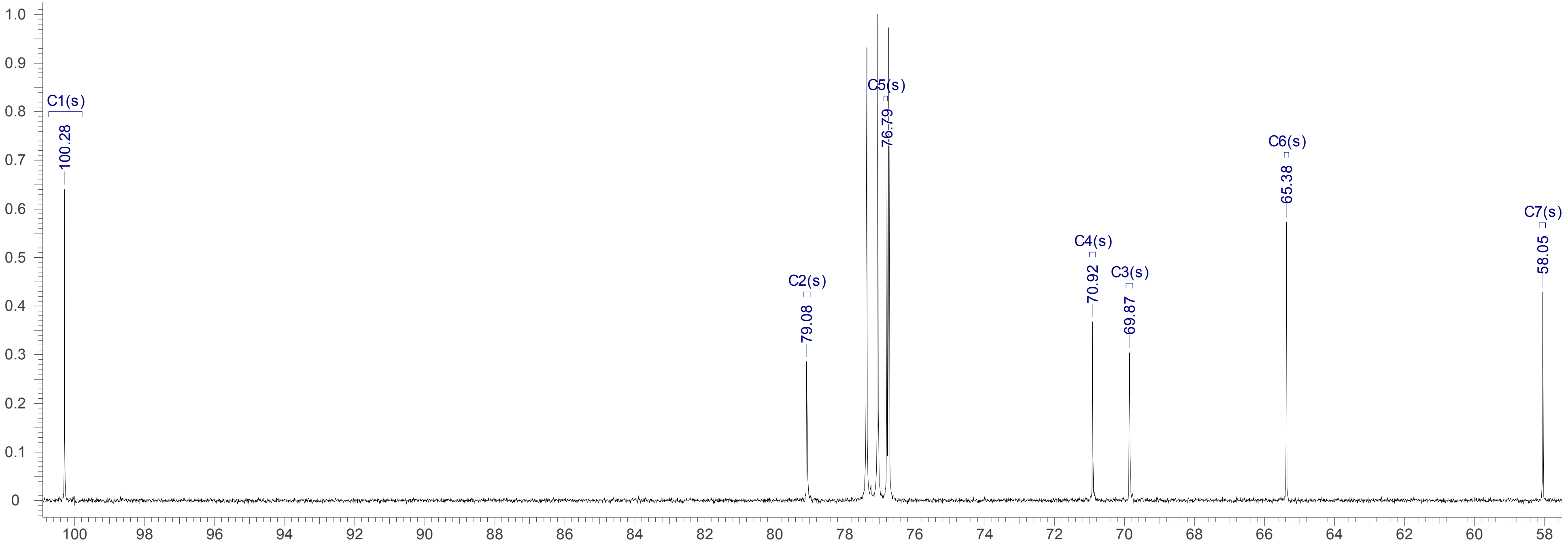
C1(s)
100.28
C2(s)
79.08
C5(s)
76.79
C4(s)
70.92
C3(s)
69.87
C6(s)
65.38
C7(s)
58.05

4.4 Compound 7

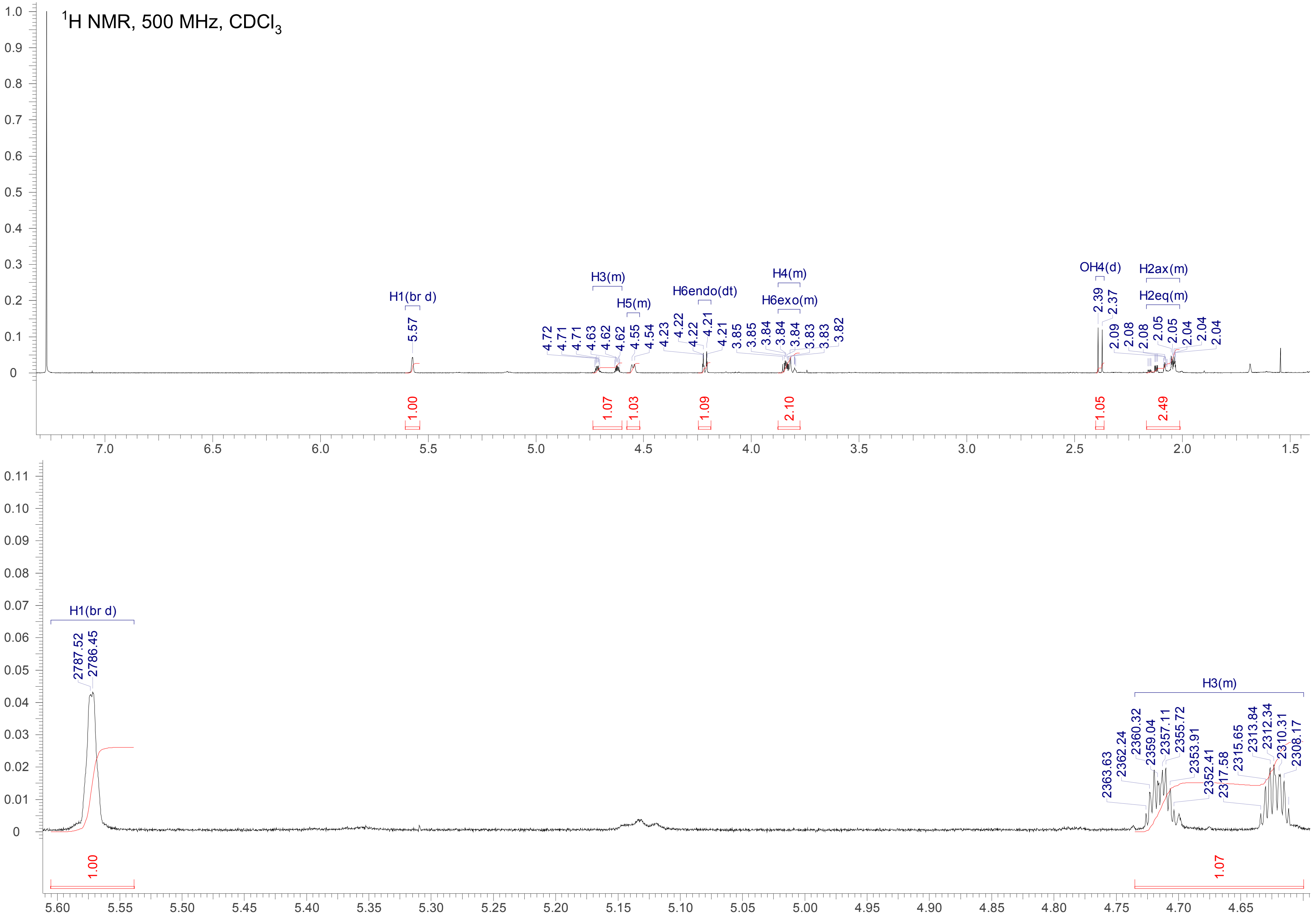

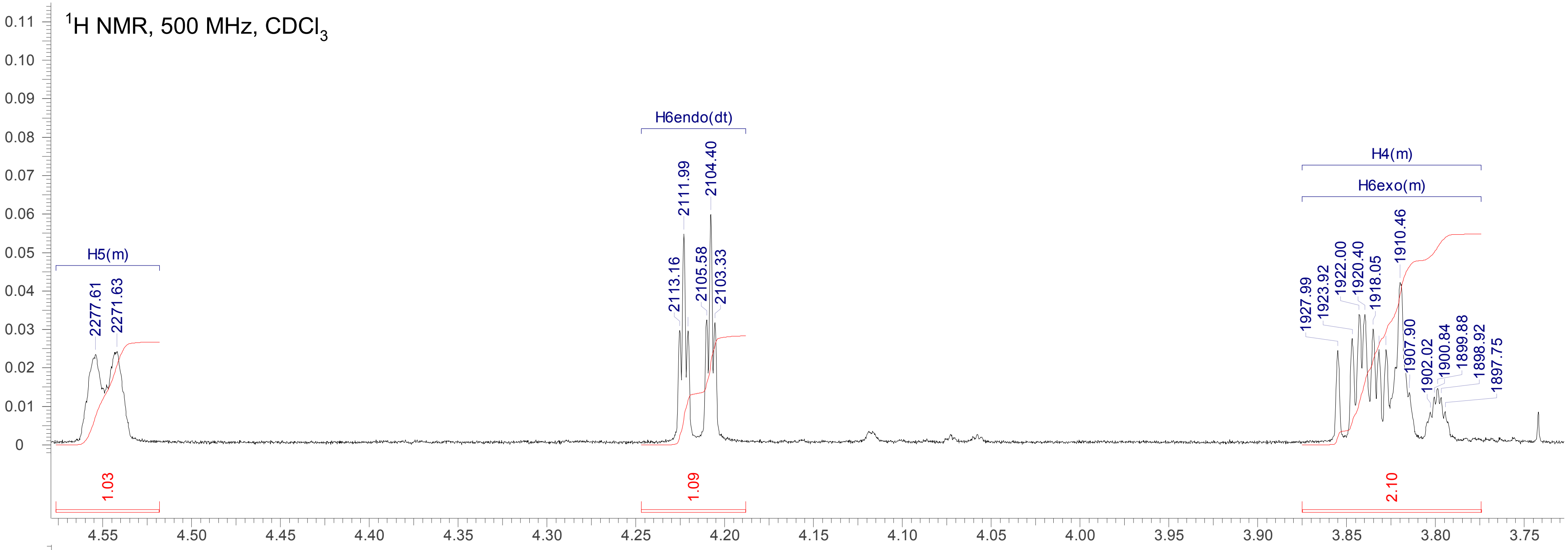

^{1}H NMR, 500 MHz, CDCl$_3$
H5(m)
2277.61
2271.63
H6endo(dt)
2113.16
2111.99
2105.58
2104.40
2103.33
H4(m)
H6exo(m)
1927.99
1923.92
1922.00
1920.40
1918.05
1910.46
1907.90
1902.02
1900.84
1899.88
1898.92
1897.75
1.03
1.09
2.10

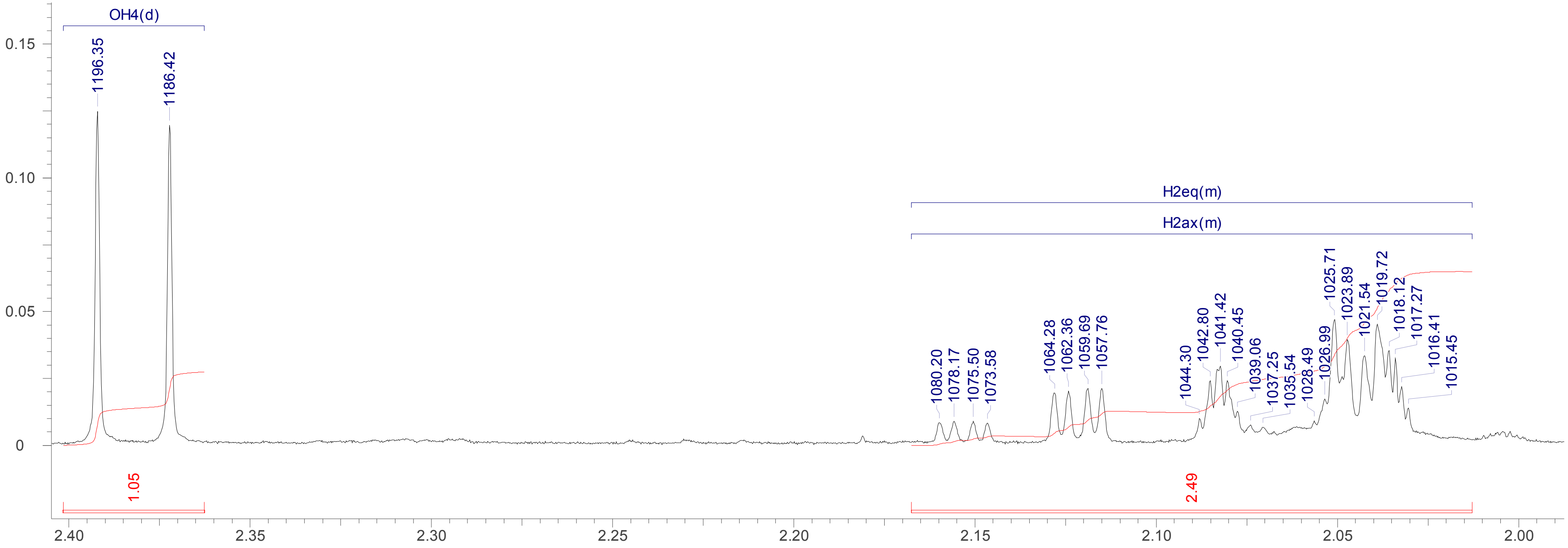

OH4(d)
1196.35
1186.42
H2eq(m)
H2ax(m)
1080.20
1078.17
1075.50
1073.58
1064.28
1062.36
1059.69
1057.76
1044.30
1042.80
1041.42
1040.45
1039.06
1037.25
1035.54
1028.49
1026.99
1025.71
1023.89
1021.54
1019.72
1018.12
1017.27
1016.41
1015.45
1.05
2.49

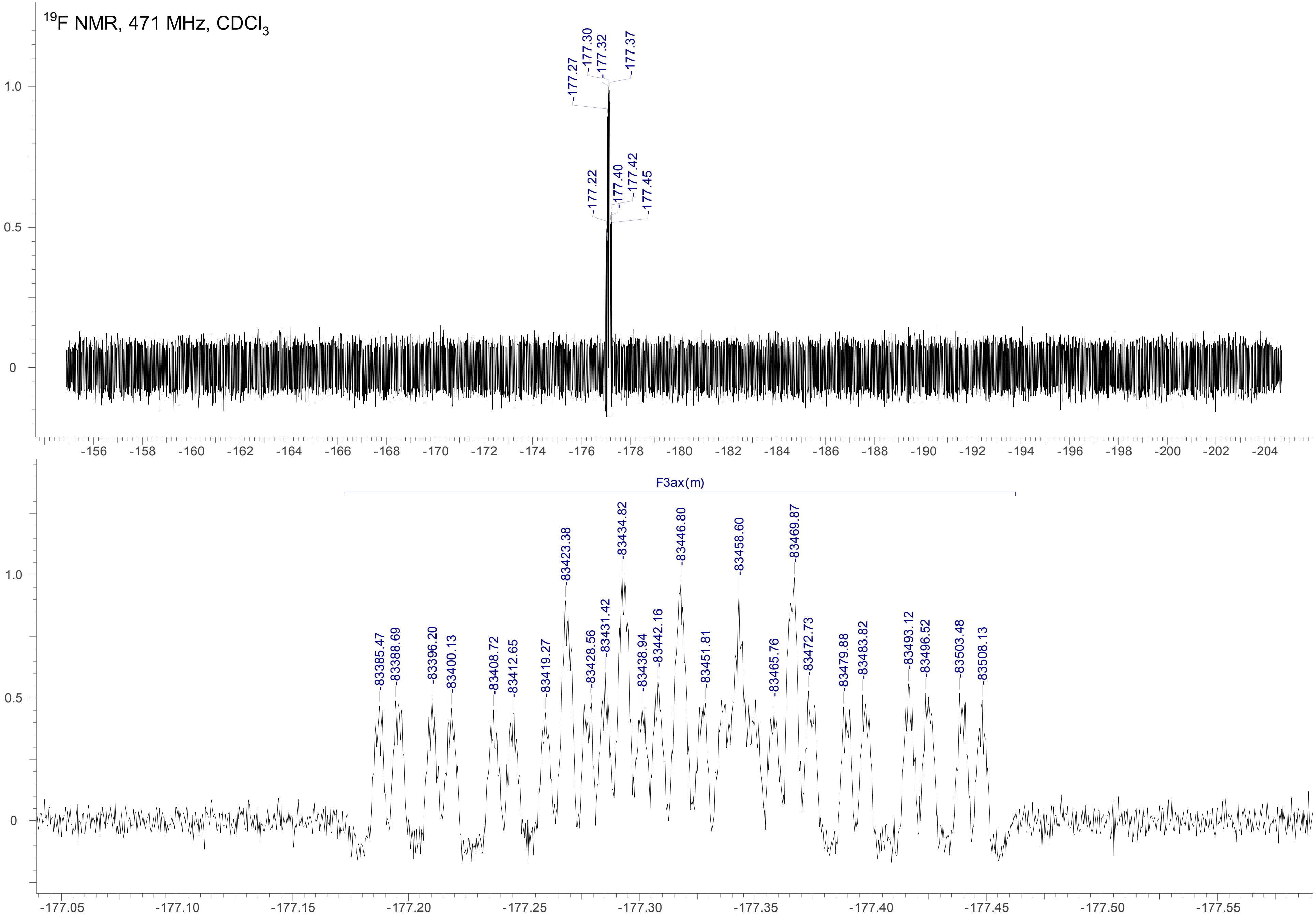

^{19}F NMR, 471 MHz, CDCl$_3$
-177.27
-177.30
-177.32
-177.37
-177.22
-177.40
-177.42
-177.45
F3ax(m)
-83385.47
-83388.69
-83396.20
-83400.13
-83408.72
-83412.65
-83419.27
-83423.38
-83428.56
-83431.42
-83434.82
-83438.94
-83442.16
-83446.80
-83451.81
-83458.60
-83465.76
-83469.87
-83472.73
-83479.88
-83483.82
-83493.12
-83496.52
-83503.48
-83508.13

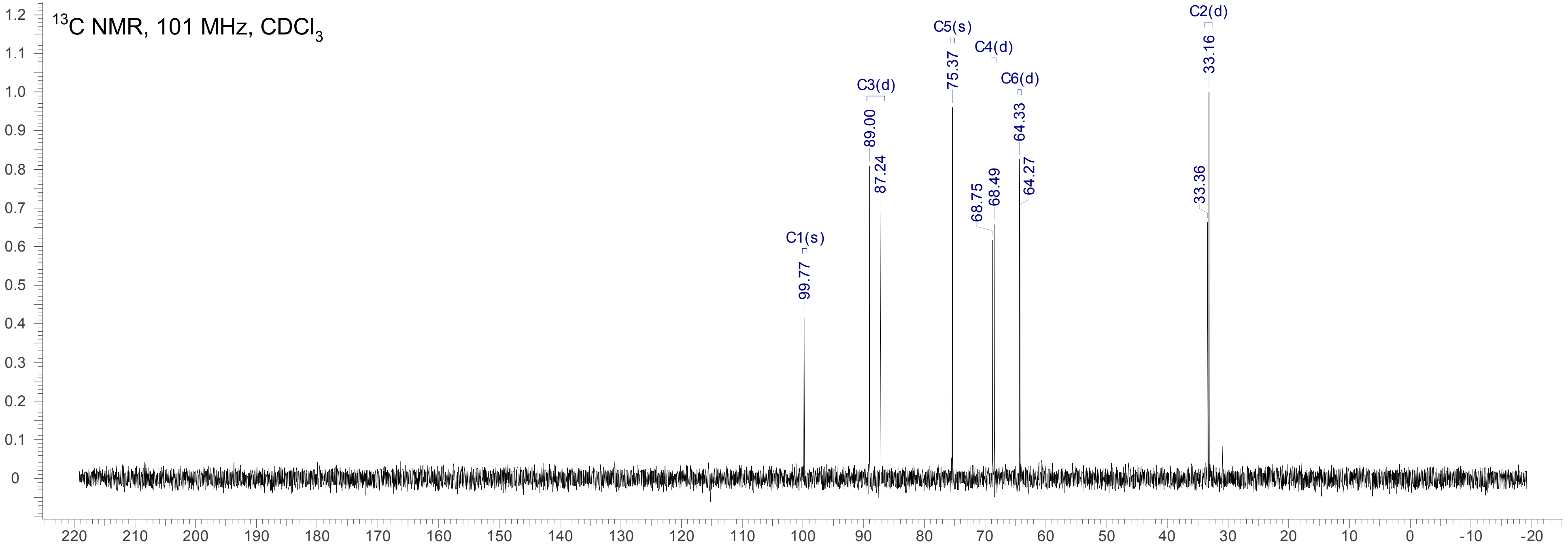
^{13}C NMR, 101 MHz, $CDCl_3$
C1(s)
99.77
C3(d)
89.00
87.24
C5(s)
75.37
C4(d)
68.75
68.49
C6(d)
64.33
64.27
C2(d)
33.16
33.36

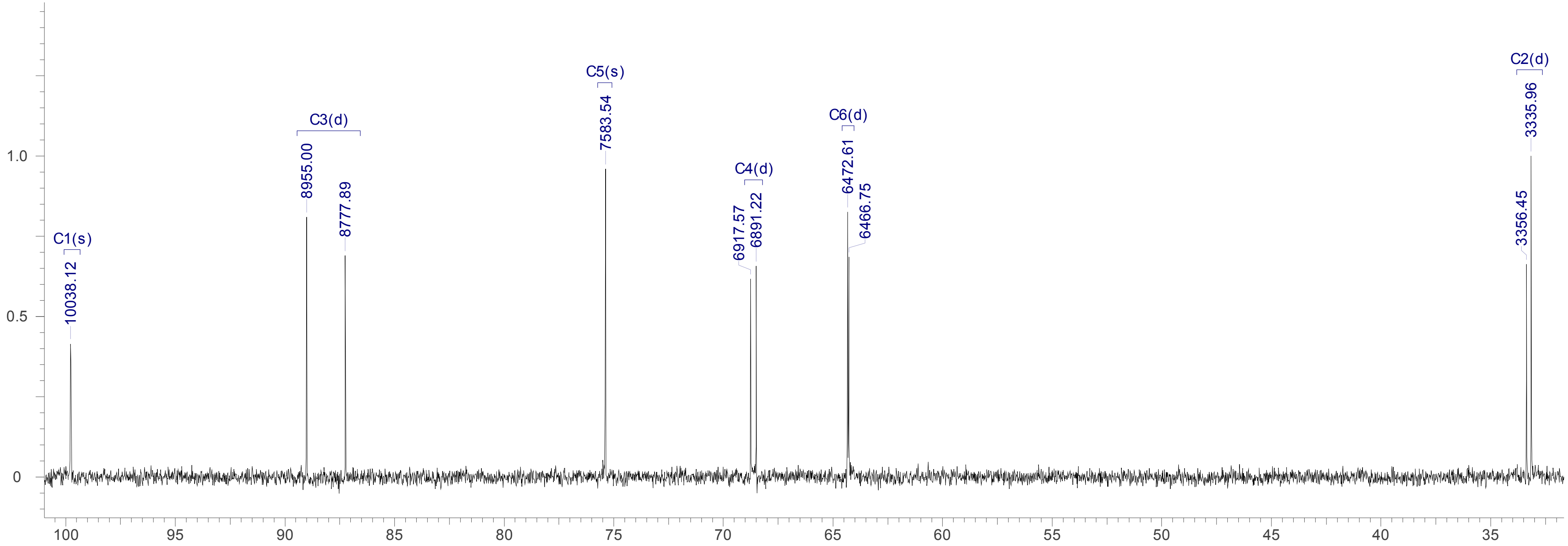
C1(s)
10038.12
C3(d)
8955.00
8777.89
C5(s)
7583.54
C4(d)
6917.57
6891.22
C6(d)
6472.61
6466.75
C2(d)
3335.96
3356.45

4.5 Compound 8

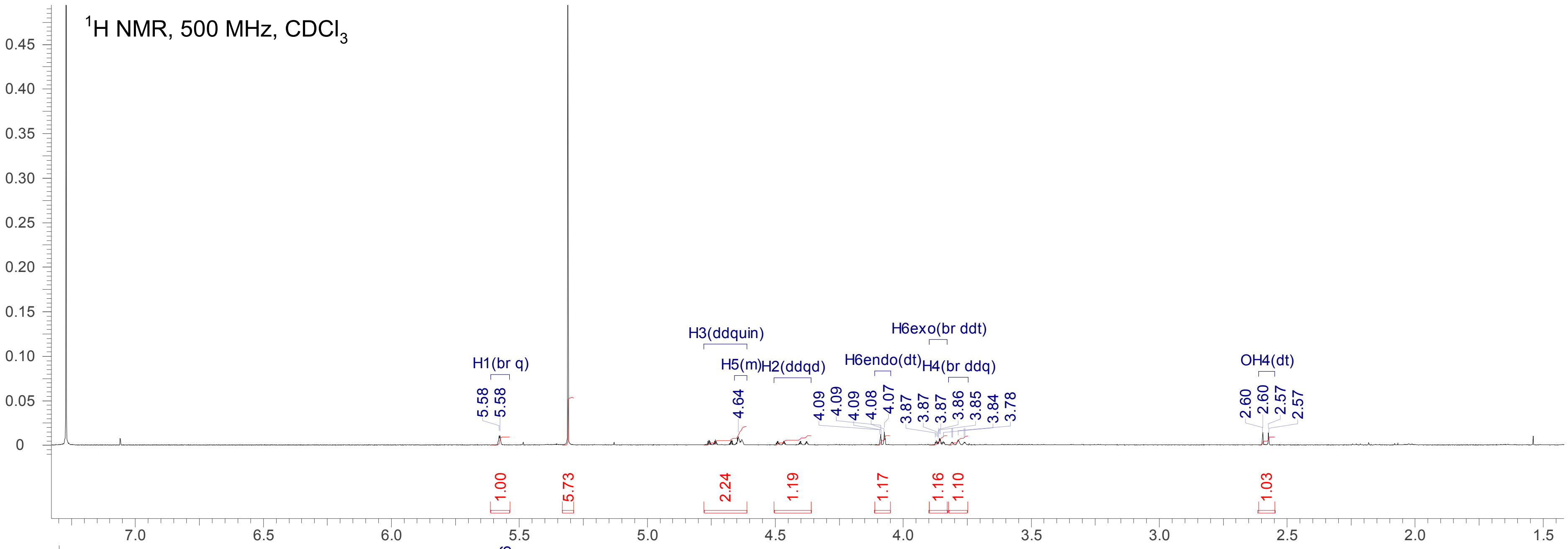

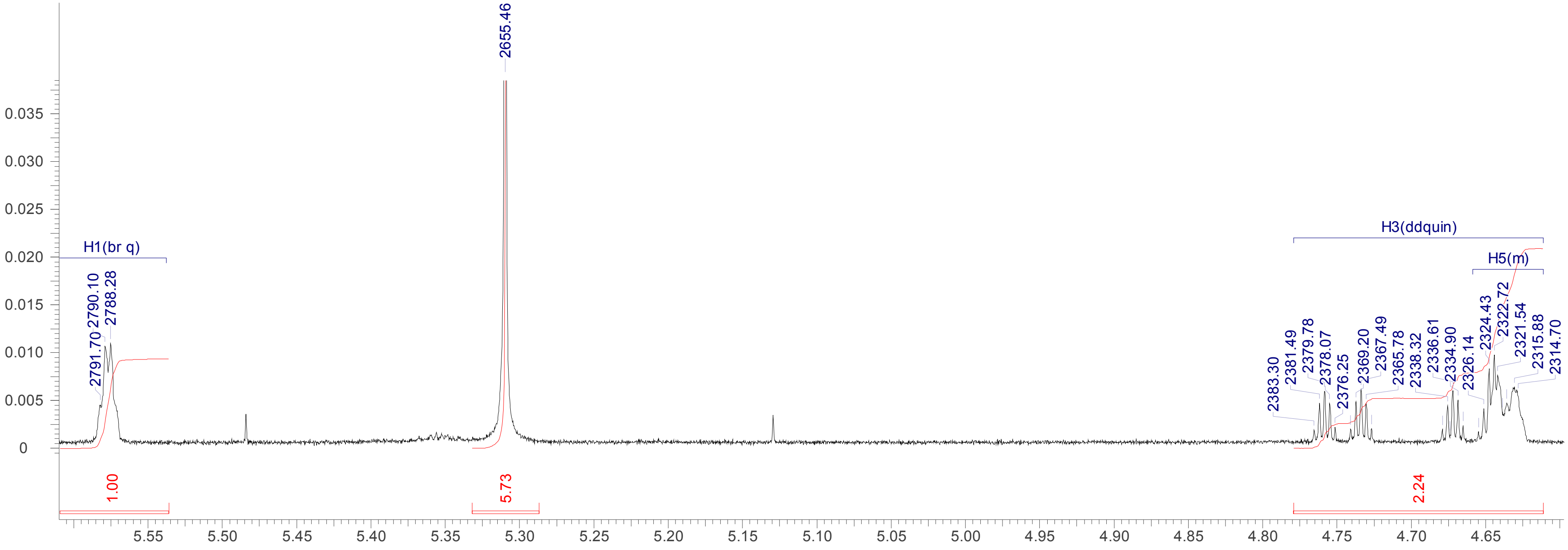

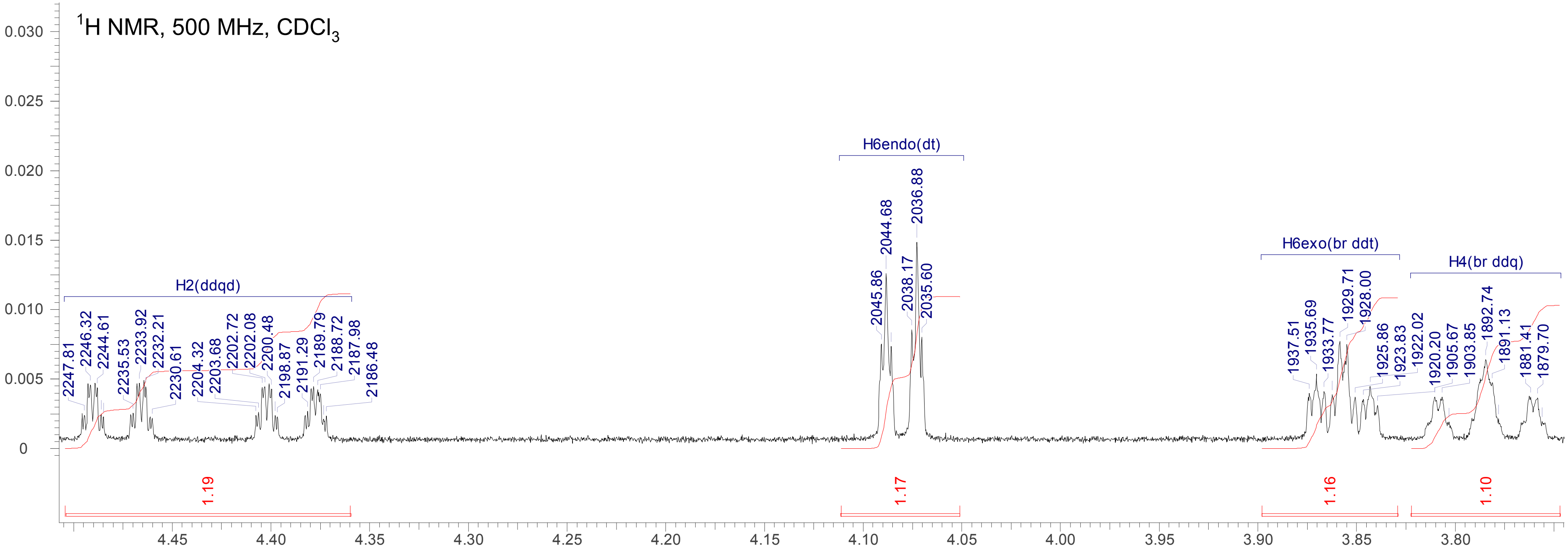
^{1}H NMR, 500 MHz, $CDCl_3$
H2(ddqd)
H6endo(dt)
H6exo(br ddt)
H4(br ddq)
1.19
1.17
1.16
1.10

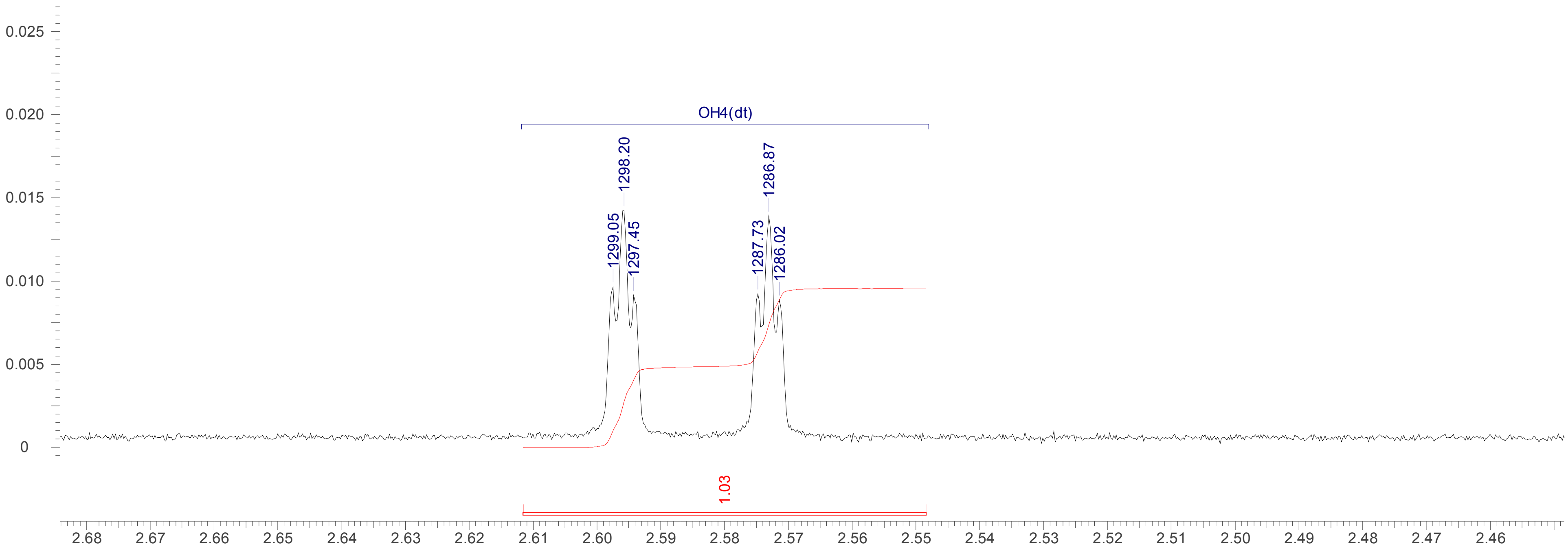
OH4(dt)
1.03

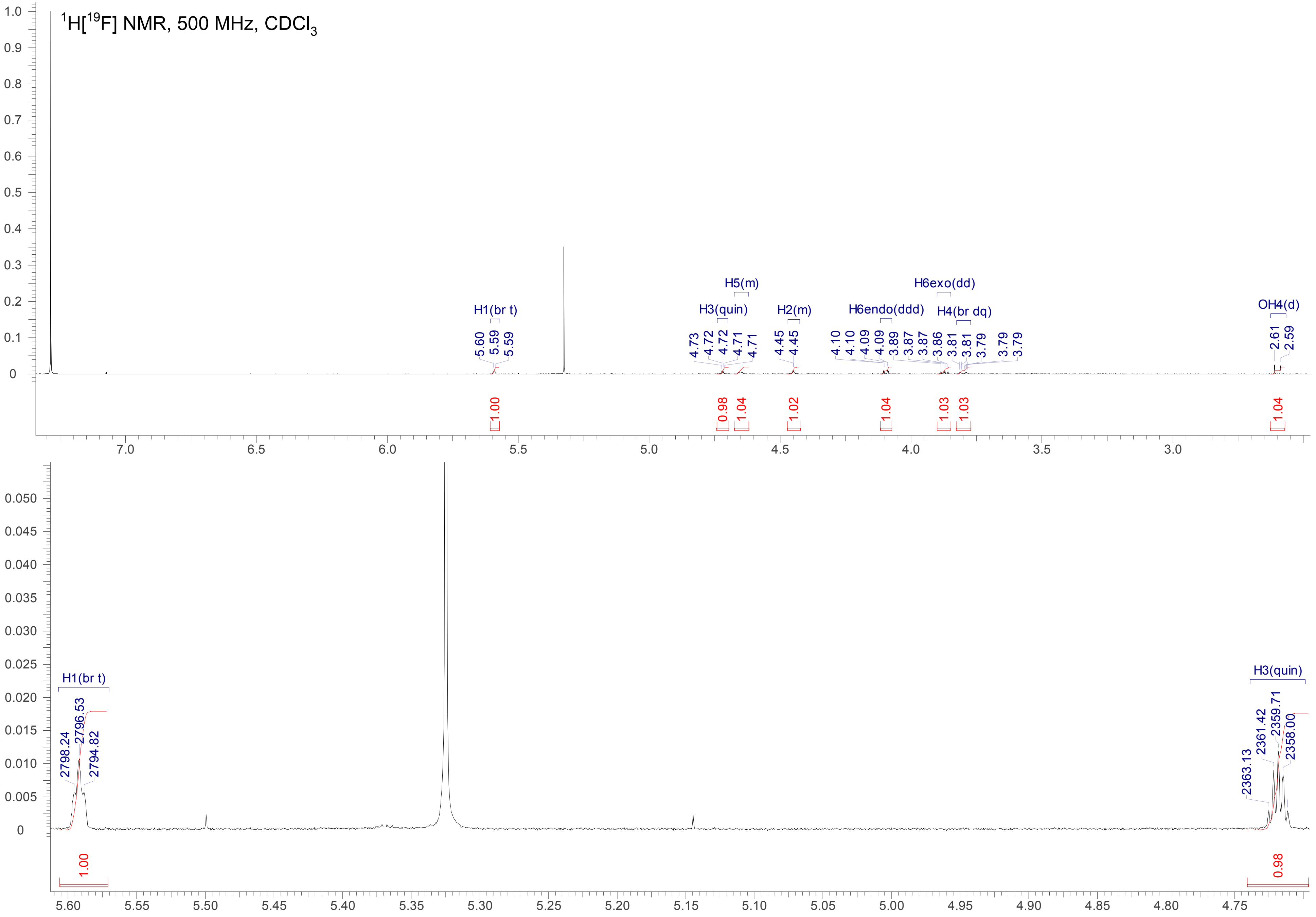

^{1}H[^{19}F] NMR, 500 MHz, CDCl$_3$
H1(br t)
H3(quin)
H5(m)
H2(m)
H6endo(ddd)
H6exo(dd)
H4(br dq)
OH4(d)
2798.24
2796.53
2794.82
2363.13
2361.42
2359.71
2358.00

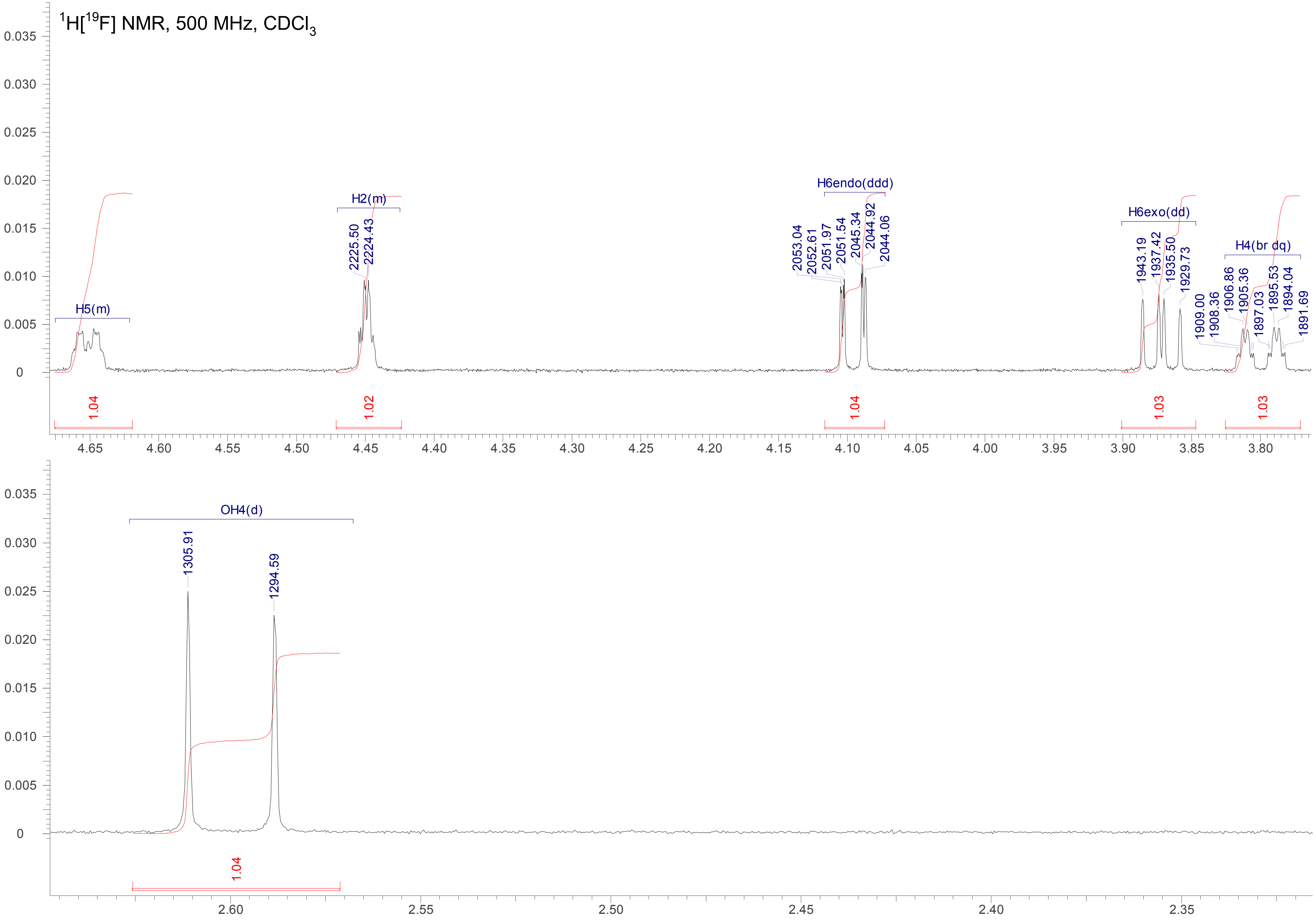

$^1H[^{19}F]$ NMR, 500 MHz, $CDCl_3$
H5(m)
H2(m)
2225.50
2224.43
H6endo(ddd)
2053.04
2052.61
2051.97
2051.54
2045.34
2044.92
2044.06
H6exo(dd)
1943.19
1937.42
1935.50
1929.73
H4(br dq)
1909.00
1908.36
1906.86
1905.36
1897.03
1895.53
1894.04
1891.69
1.04
1.02
1.04
1.03
1.03
OH4(d)
1305.91
1294.59
1.04

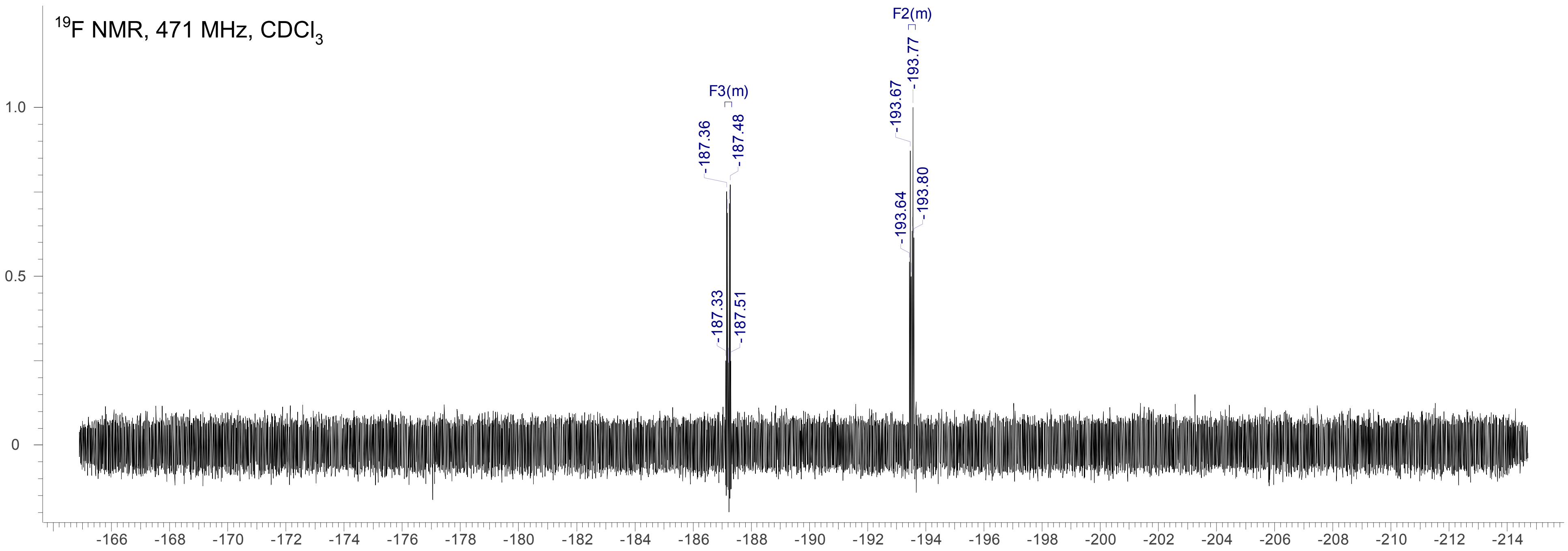

^{19}F NMR, 471 MHz, $CDCl_3$
F2(m)
F3(m)
-193.77
-193.67
-193.80
-193.64
-187.36
-187.48
-187.33
-187.51

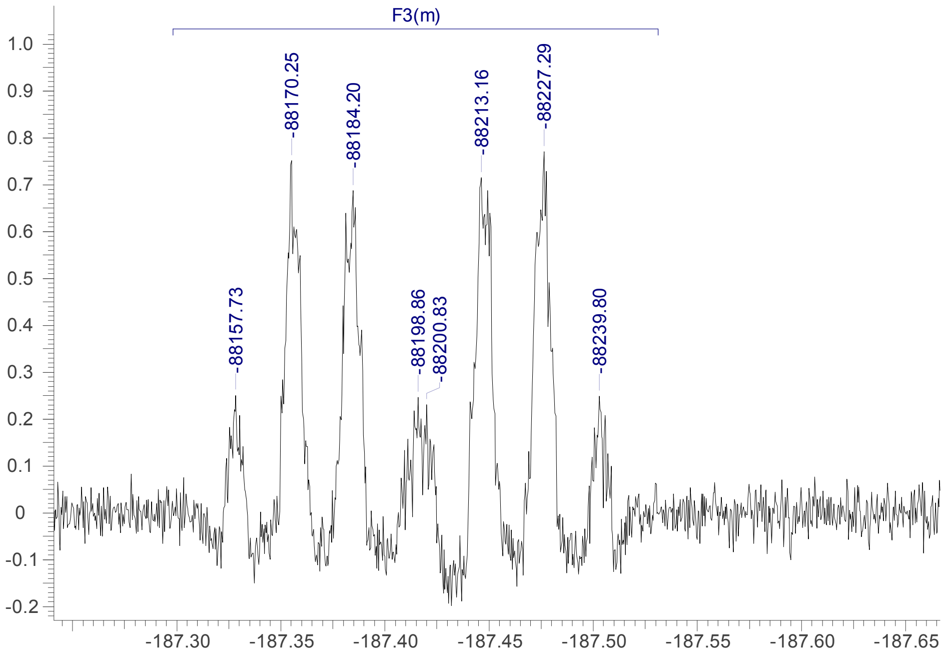

F3(m)
-88157.73
-88170.25
-88184.20
-88198.86
-88200.83
-88213.16
-88227.29
-88239.80

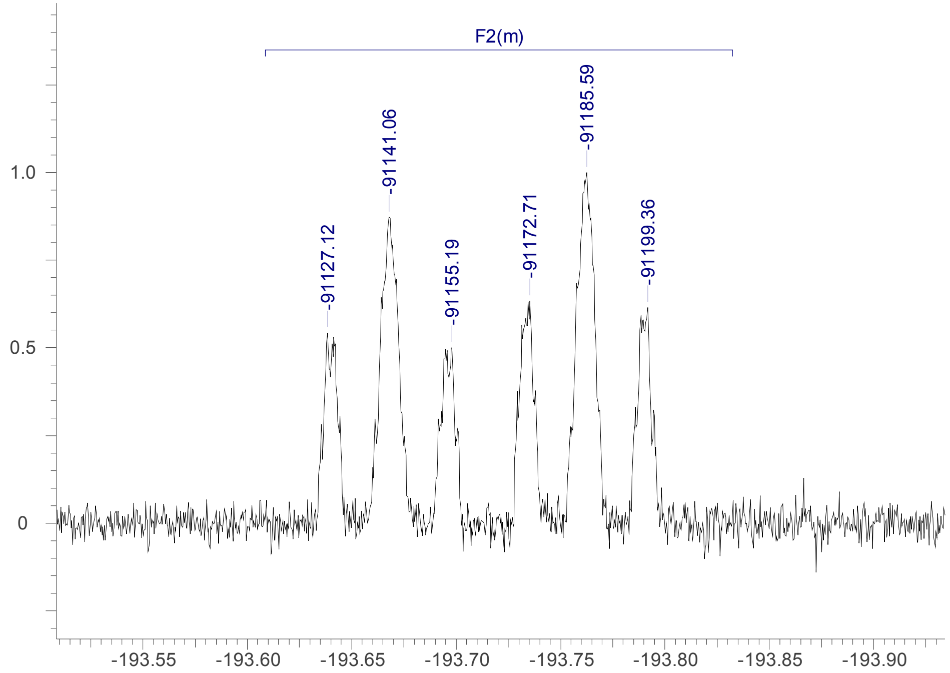

F2(m)
-91127.12
-91141.06
-91155.19
-91172.71
-91185.59
-91199.36

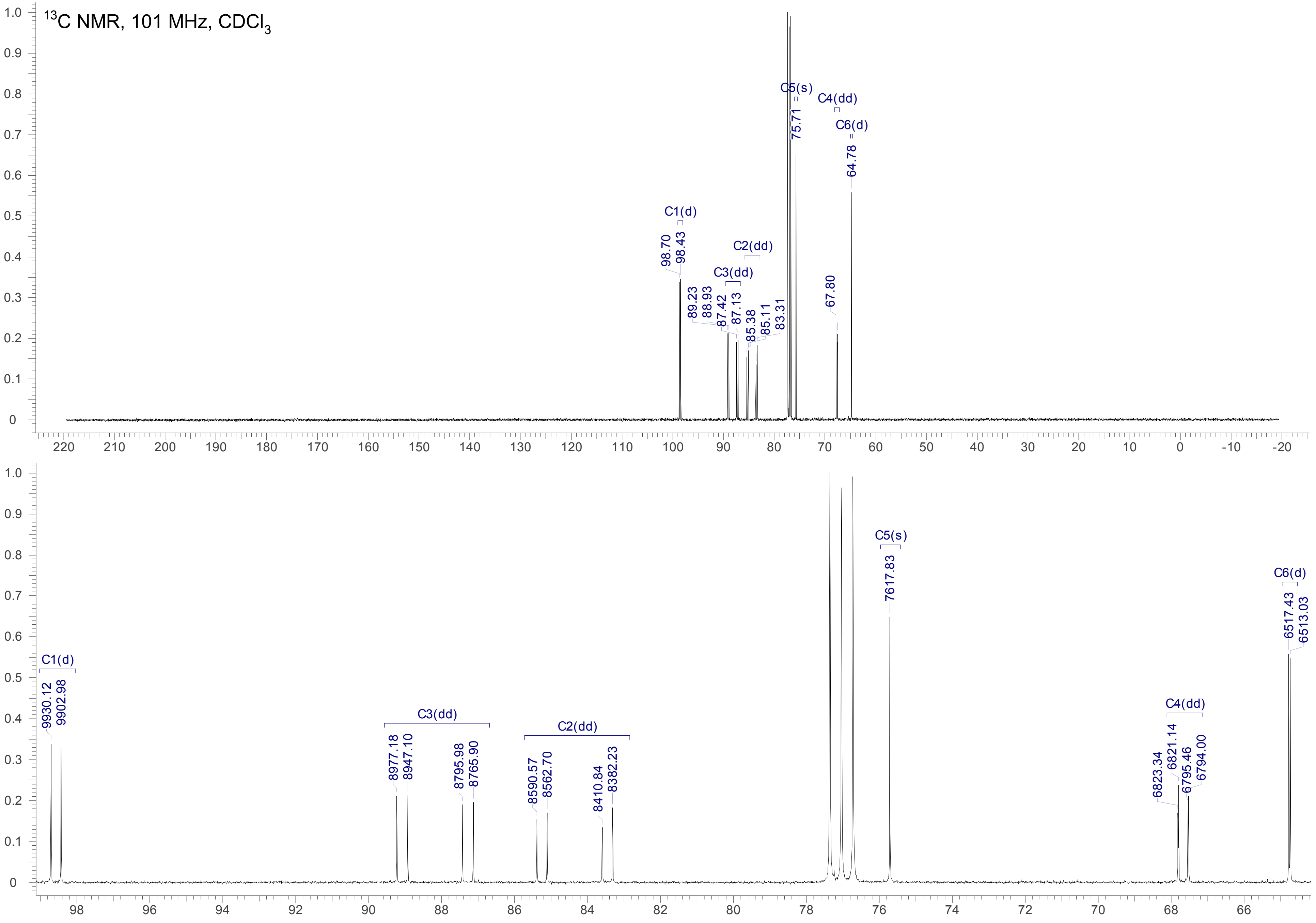

13C NMR, 101 MHz, CDCl3
C1(d)
98.70
98.43
C2(dd)
C3(dd)
89.23
88.93
87.42
87.13
85.38
85.11
83.31
C5(s)
75.71
C4(dd)
C6(d)
64.78
67.80
C1(d)
9930.12
9902.98
C3(dd)
8977.18
8947.10
8795.98
8765.90
C2(dd)
8590.57
8562.70
8410.84
8382.23
C5(s)
7617.83
C4(dd)
6823.34
6821.14
6795.46
6794.00
C6(d)
6517.43
6513.03

4.6 Compound 9

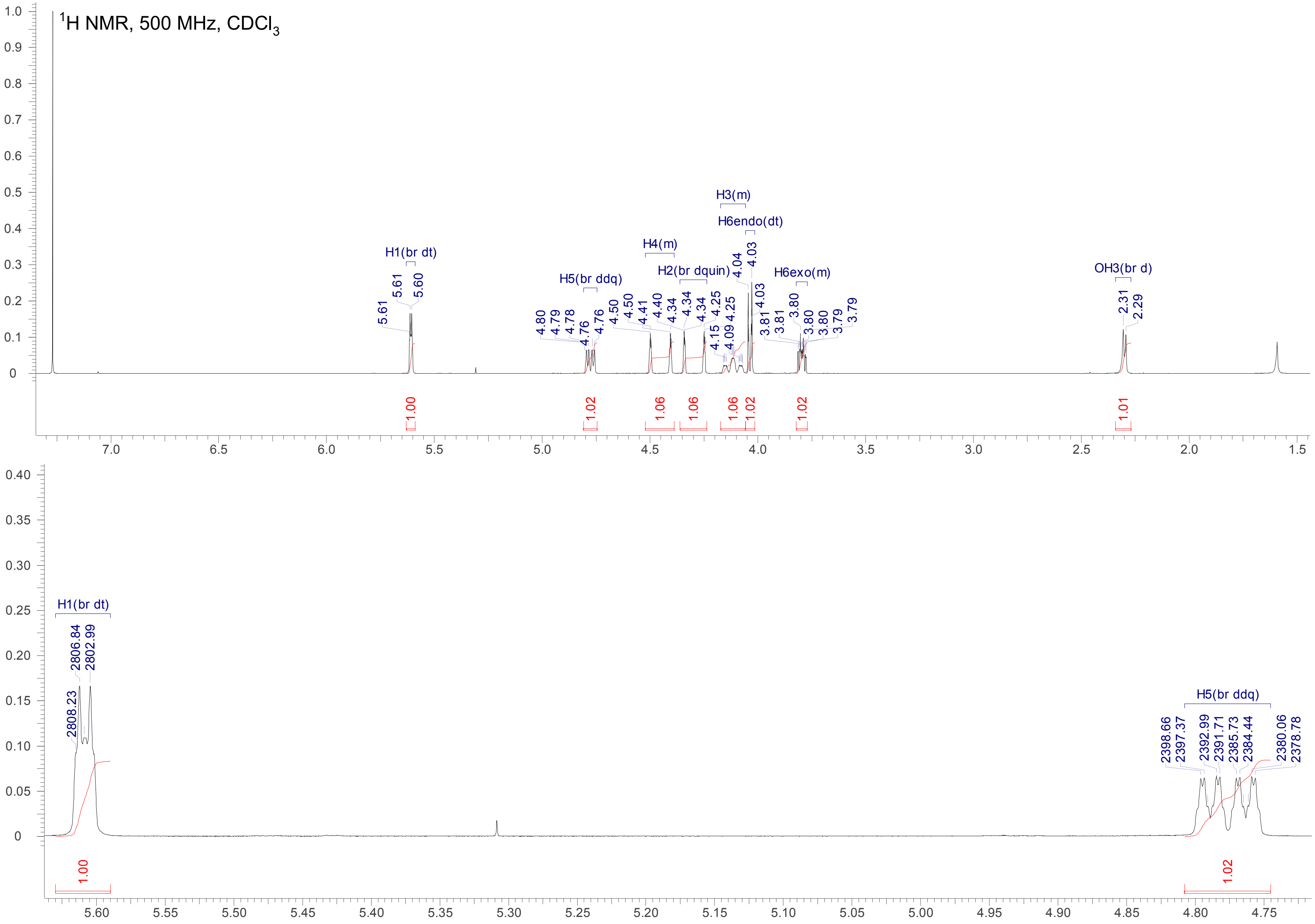

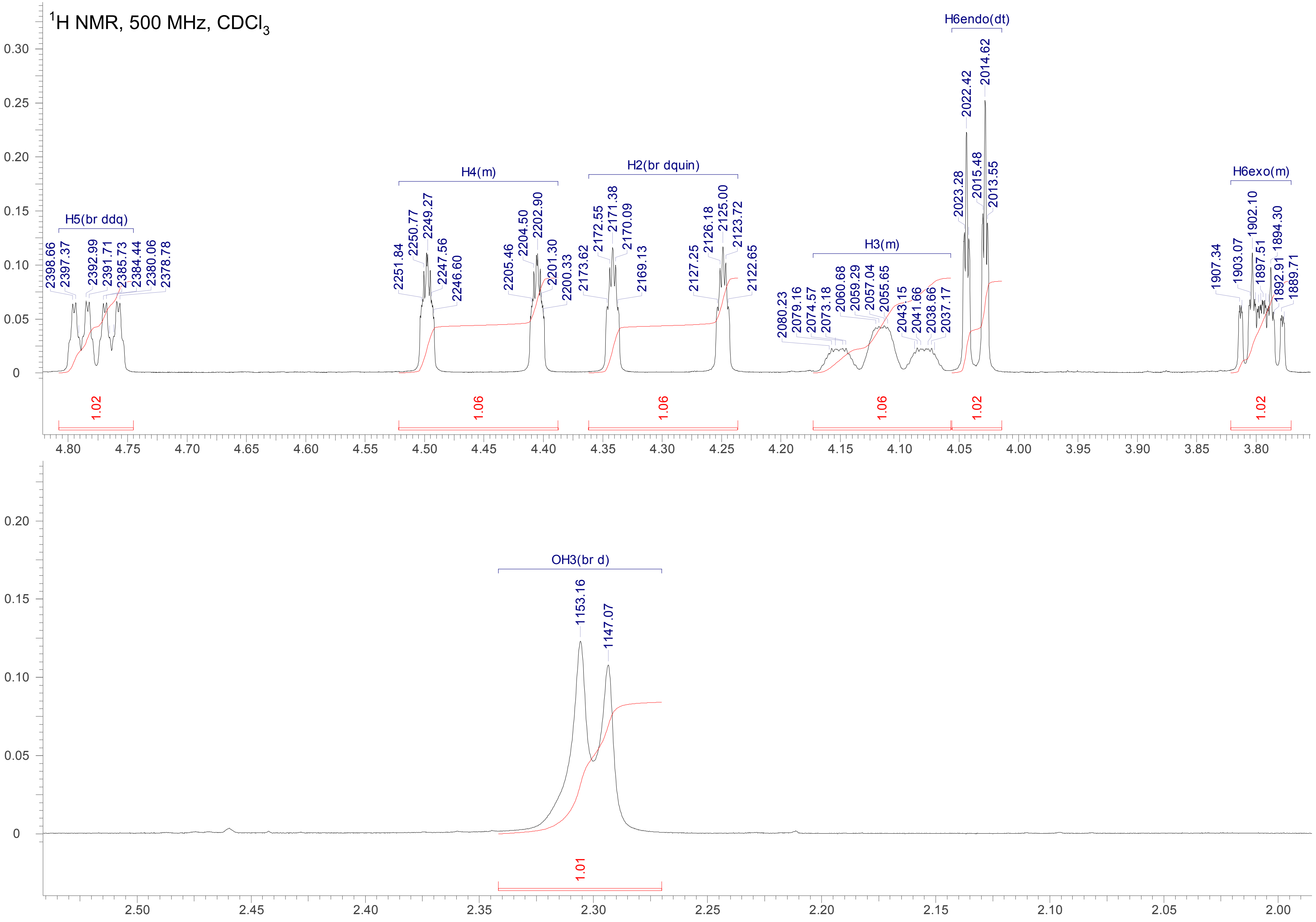
1H NMR, 500 MHz, CDCl3
H5(br ddq)
H4(m)
H2(br dquin)
H3(m)
H6endo(dt)
H6exo(m)
OH3(br d)
1.02
1.06
1.06
1.06
1.02
1.02
1.01

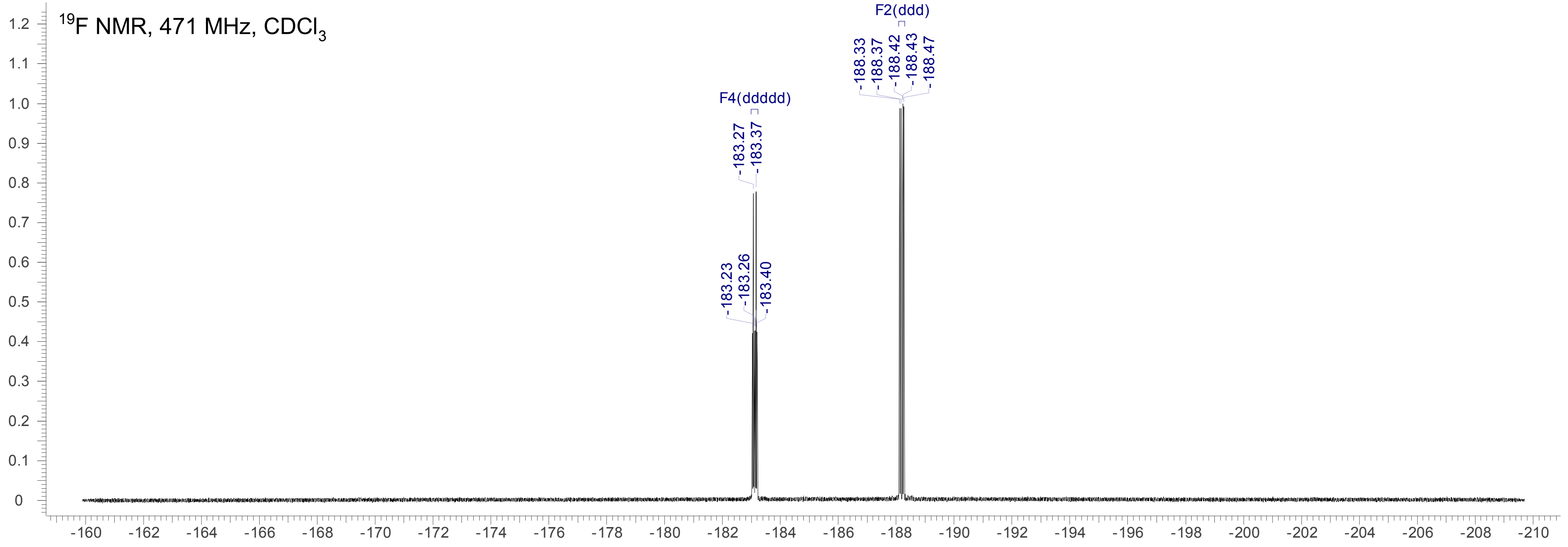

19F NMR, 471 MHz, CDCl3
F4(ddddd)
F2(ddd)
-183.27
-183.37
-183.23
-183.26
-183.40
-188.33
-188.37
-188.42
-188.43
-188.47

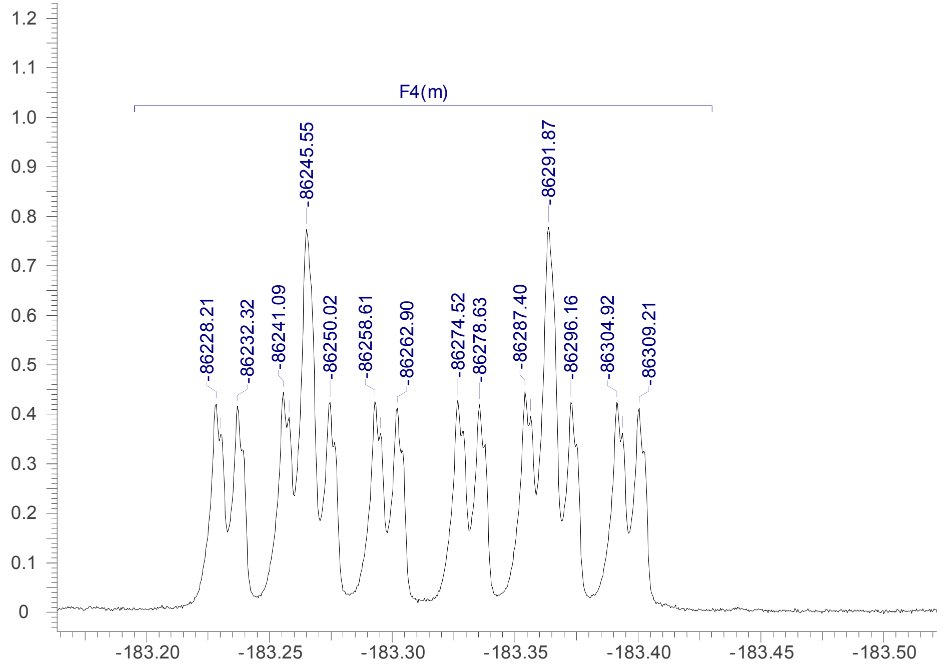

F4(m)
-86228.21
-86232.32
-86241.09
-86245.55
-86250.02
-86258.61
-86262.90
-86274.52
-86278.63
-86287.40
-86291.87
-86296.16
-86304.92
-86309.21

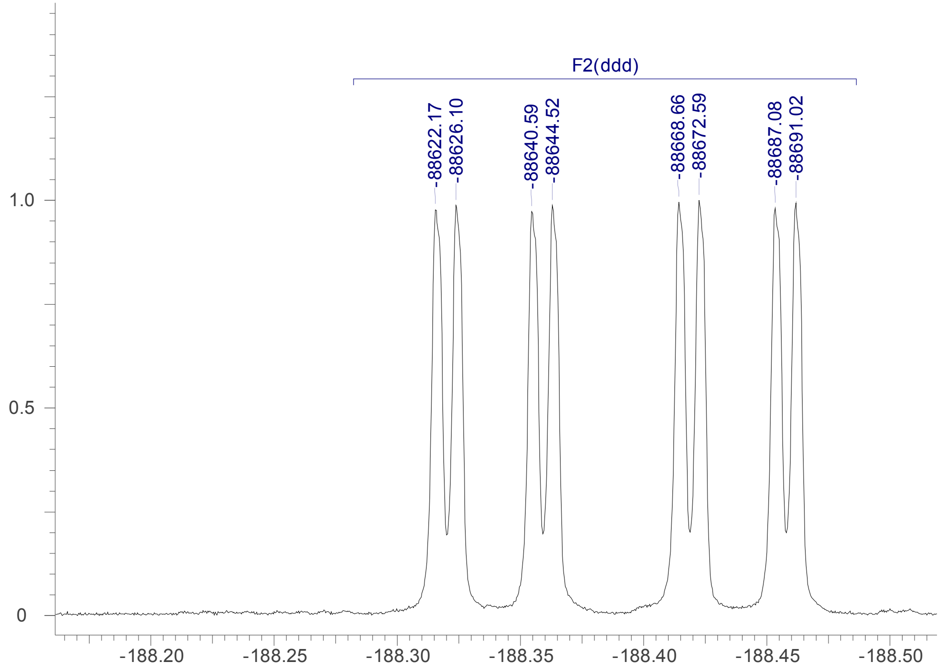

F2(ddd)
-88622.17
-88626.10
-88640.59
-88644.52
-88668.66
-88672.59
-88687.08
-88691.02

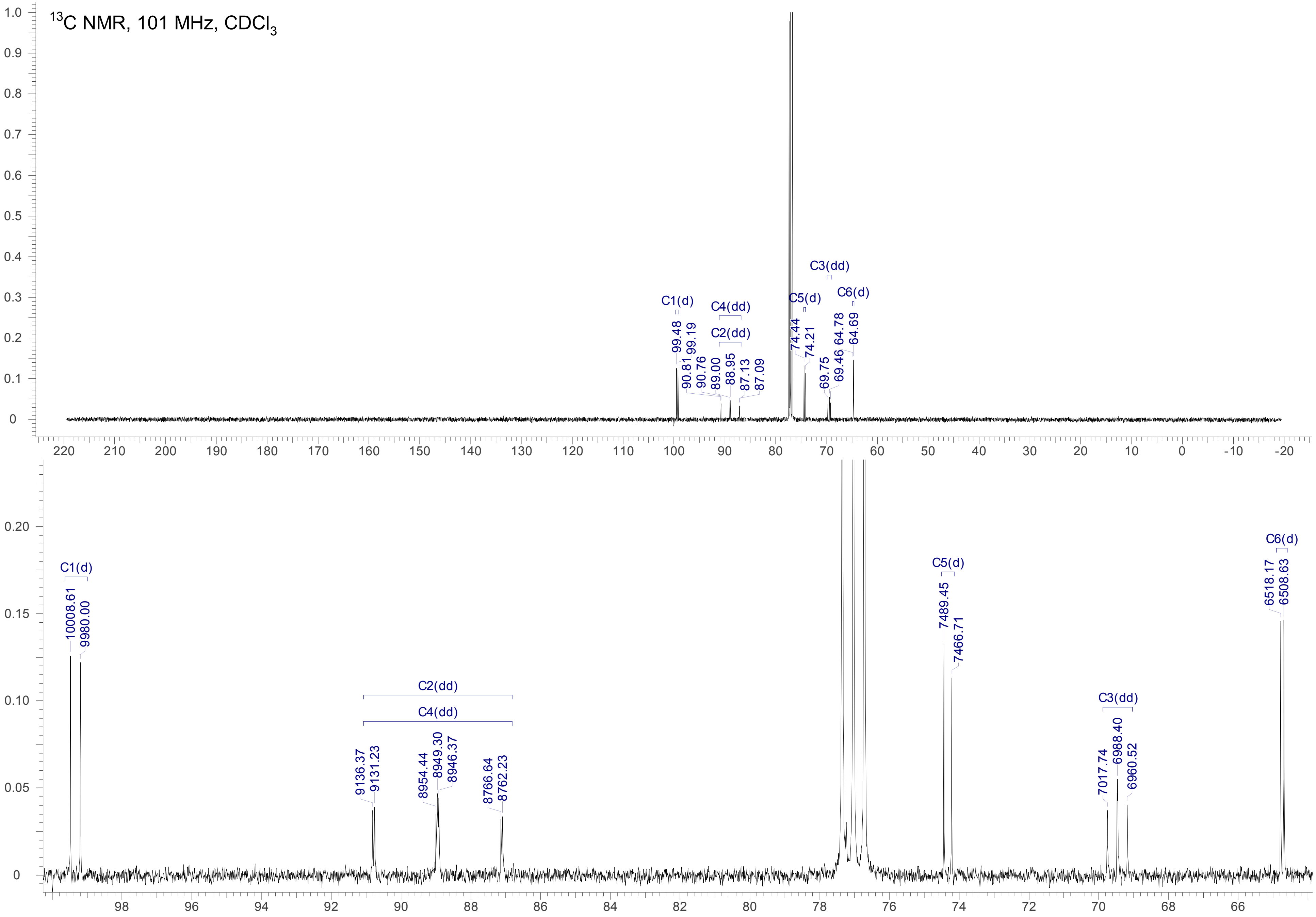

^{13}C NMR, 101 MHz, $CDCl_3$
C1(d)
99.48
99.19
C4(dd)
C2(dd)
90.81
90.76
89.00
88.95
87.13
87.09
C5(d)
74.44
74.21
C3(dd)
69.75
69.46
C6(d)
64.78
64.69
10008.61
9980.00
9136.37
9131.23
8954.44
8949.30
8946.37
8766.64
8762.23
7489.45
7466.71
7017.74
6988.40
6960.52
6518.17
6508.63

4.7 Compound 10

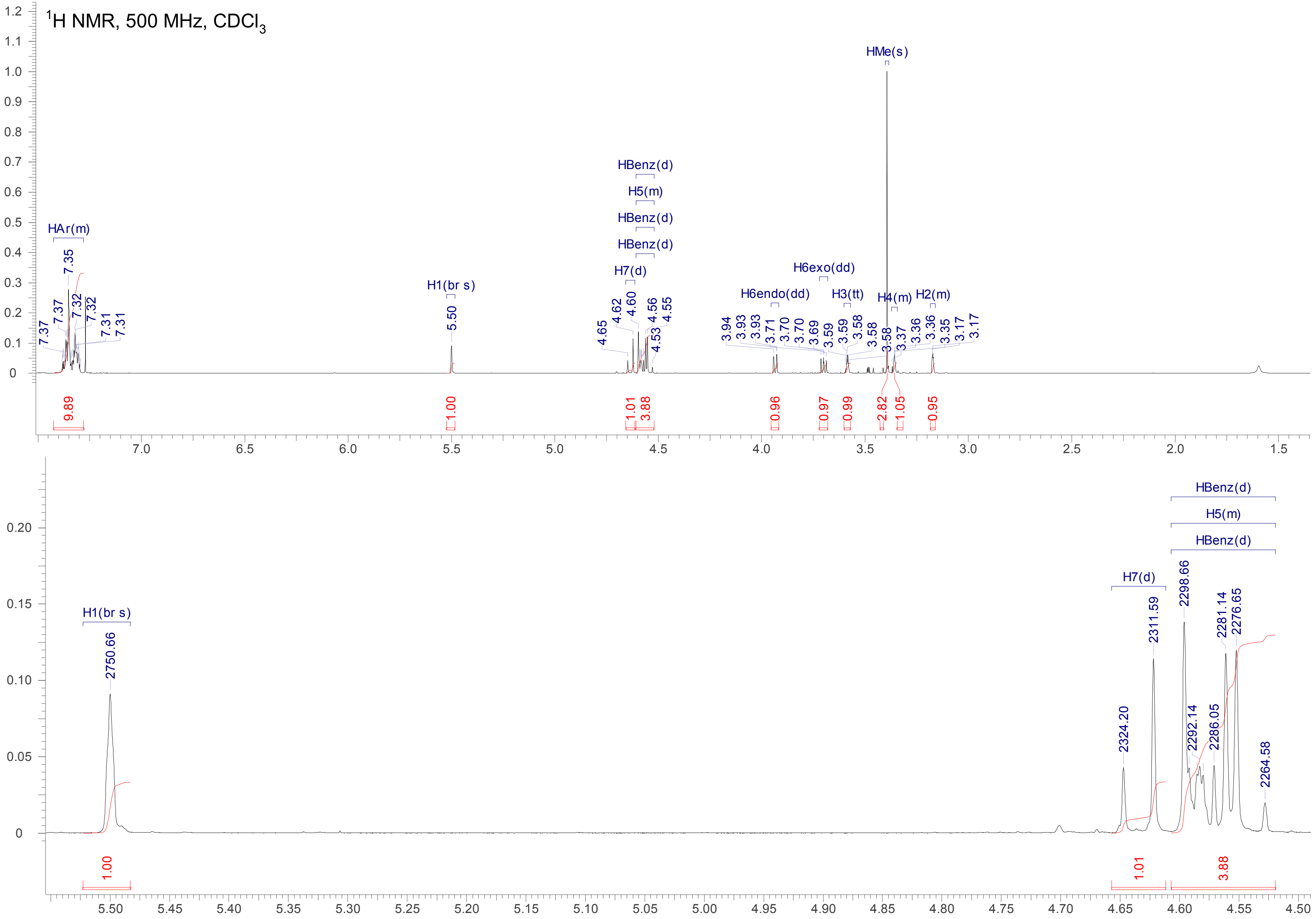

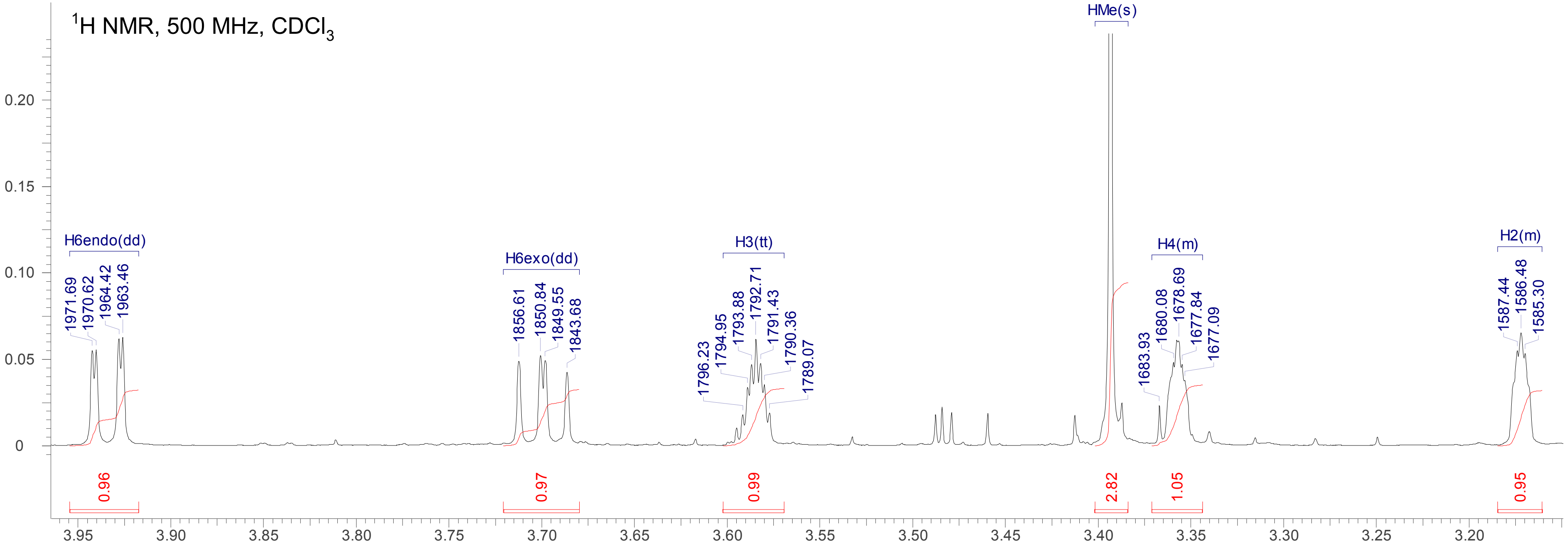

^{1}H NMR, 500 MHz, CDCl$_3$
H6endo(dd)
H6exo(dd)
H3(tt)
HMe(s)
H4(m)
H2(m)
1971.69
1970.62
1964.42
1963.46
1856.61
1850.84
1849.55
1843.68
1796.23
1794.95
1793.88
1792.71
1791.43
1790.36
1789.07
1683.93
1680.08
1678.69
1677.84
1677.09
1587.44
1586.48
1585.30
0.96
0.97
0.99
2.82
1.05
0.95

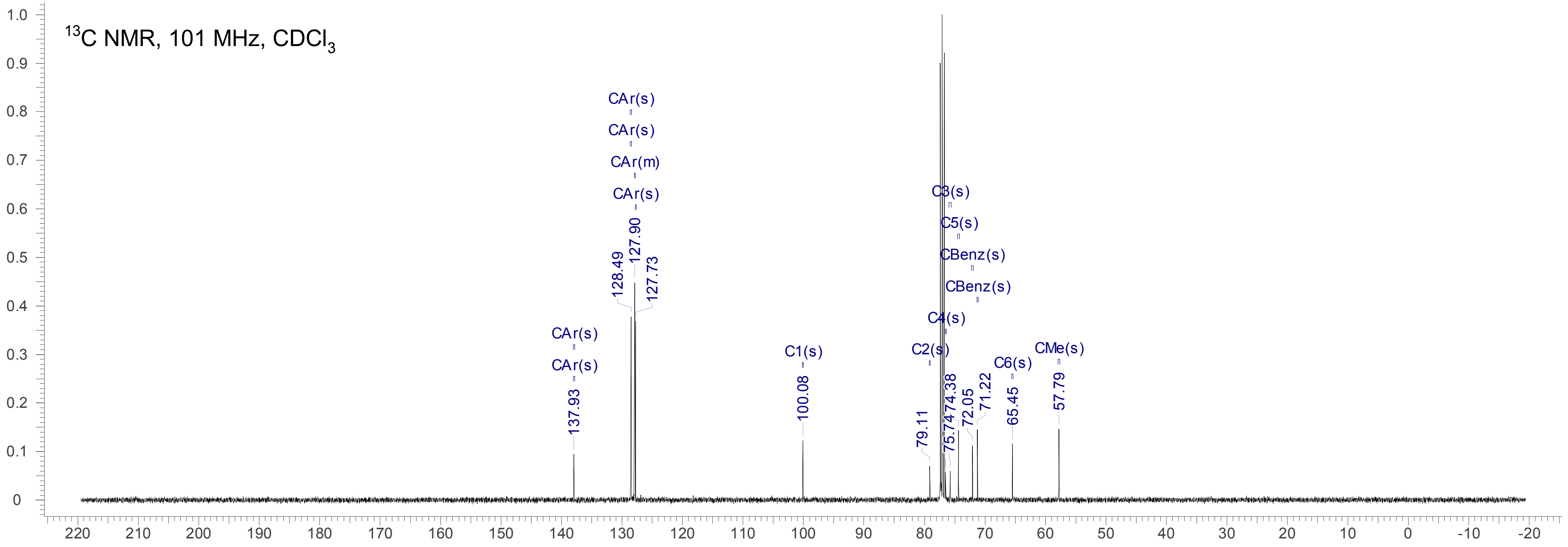

^{13}C NMR, 101 MHz, CDCl$_3$
CAr(s)
CAr(s)
CAr(m)
CAr(s)
CAr(s)
CAr(s)
C1(s)
C3(s)
C5(s)
CBenz(s)
CBenz(s)
C4(s)
C2(s)
C6(s)
CMe(s)
137.93
128.49
127.90
127.73
100.08
79.11
75.74
74.38
72.05
71.22
65.45
57.79

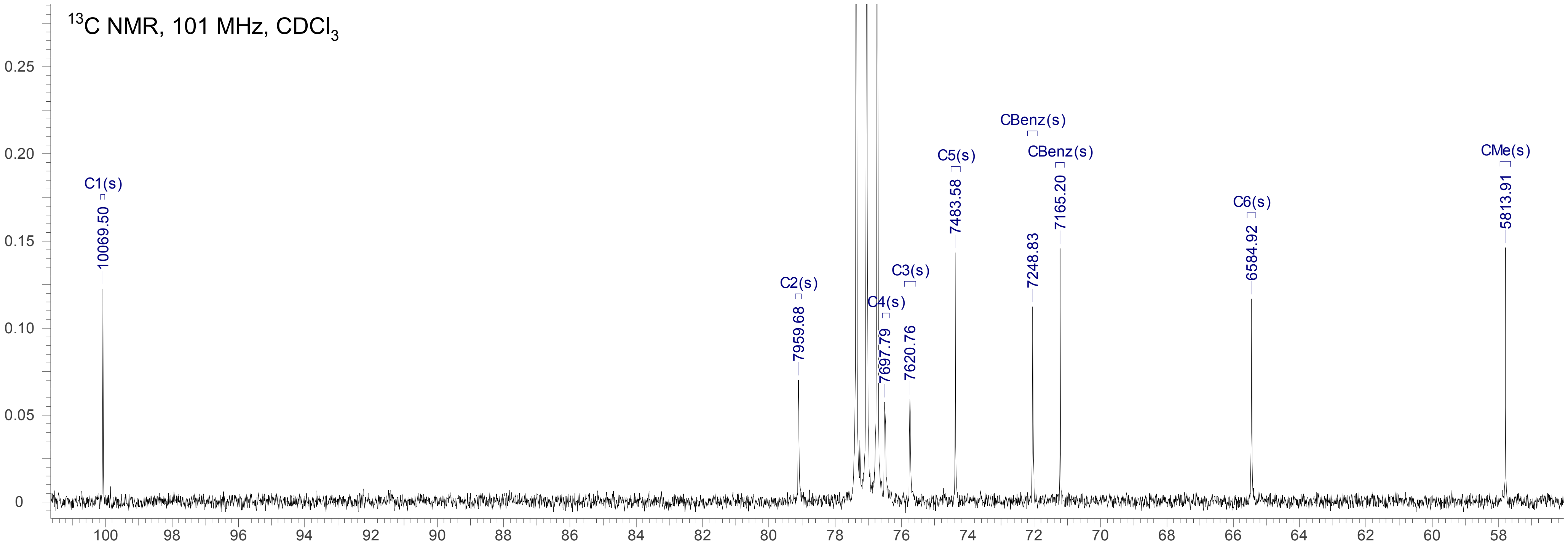

(1) Pacak, J. P., J.; Tocik, Z.; Cerny, M. . *Collect. Czech. Chem. Commun.* **1972**, *37*, 2589.

(2) Ronnols, J.; Manner, S.; Siegbahn, A.; Ellervik, U.; Widmalm, G. *Org. Biomol. Chem.* **2013**, *11*, 5465.

(3) Mtashobya, L.; Quiquempoix, L.; Linclau, B. *J. Fluorine Chem.* **2015**, *171*, 92.

(4) Van Rijsbergen, R.; Anteunis, M. J. O.; De Bruyn, A. *Carbohydr. Res.* **1982**, *105*, 269.

(5) Hori, H.; Nishida, Y.; Ohrui, H.; Meguro, H. *J. Org. Chem.* **1989**, *54*, 1346.

(6) Wollwage, P. C.; Seib, P. A. *Carbohydr. Res.* **1969**, *10*, 589.

(7) Sarda, P.; Escribano, F. C.; José Alves, R.; Olesker, A.; Lukacs, G. *J. Carbohydr. Chem.* **1989**, *8*, 115.
